# OPRK1 drives SLC9A3R1 progression to neuroendocrine prostate cancer

**DOI:** 10.1038/s41419-025-08279-4

**Published:** 2025-12-07

**Authors:** Linghui Liang, Zhiyi Shen, Yuwei Zhang, Yifei Cheng, Bing Yao, Ninghan Feng, Ruizhe Zhao

**Affiliations:** 1 Department of Urology, Affiliated Wuxi No.2 Hospital, Nanjing Medical University & Jiangnan University Medical Center, Wuxi, Jiangsu China; 2 Department of Urology, the First Affiliated Hospital of Nanjing Medical University, Nanjing, Jiangsu China; 3 Nantong University Medical School, Nantong, China; 4 Department of Urology, School of Medicine, Affiliated ZhongDa Hospital of Southeast University, Nanjing, Jiangsu China; 5 National Experimental Teaching Center of Basic Medical Science, School of Basic Medical Sciences, Nanjing Medical University, Nanjing, China; 6 Department of Medical Genetics, School of Basic Medical Sciences, Nanjing Medical University, Nanjing, China

**Keywords:** Prostate cancer, Oncogenes

## Abstract

Neuroendocrine differentiation (NED) plays a critical role in endocrine therapy resistance and dismal outcomes among prostate cancer (PCa) patients. The emergence of treatment-induced neuroendocrine prostate cancers (t-NEPCs) with the utilization of second-generation androgen receptor (AR) pathway inhibitors (ARPIs) poses a significant challenge, as the molecular underpinnings remain elusive. Here, our investigation unveils a close correlation between heightened levels of opioid receptor membrane protein OPRK1 and treatment-induced NED (t-NED), alongside an adverse prognosis in PCa cohorts. Our findings illuminate that AR represses OPRK1 transcription by binding to its promoter, a regulation amenable to reversal via ARPI administration. Further exploration reveals that OPRK1 stimulation triggers autophagic degradation of REST upon up-regulation and interaction with SLC9A3R1, thereby instigating NED. In essence, OPRK1 experiences negative control by AR and emerges as a pivotal instigator of t-NED. Combining JTC-801 with CQ successfully impedes NEPC progression by impacting the OPRK1/SLC9A3R1/autophagy/REST axis. Our study accentuates OPRK1 as a novel therapeutic target for PCa management and furnishes profound insights into the pathogenesis of t-NEPC.

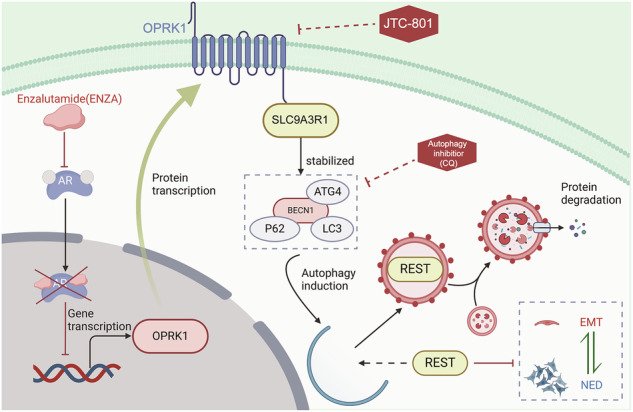

## Introduction

Prostate cancer (PCa) is one of the most prevalent malignancies and represents the second leading cause of cancer-related mortality among men worldwide [1]. Androgen deprivation therapy (ADT) remains the cornerstone treatment for recurrent disease, achieving substantial initial responses and prolonging survival [2, 3]. The advent of next-generation androgen receptor pathway inhibitors (ARPIs), including abiraterone and enzalutamide (ENZA), has further improved outcomes for patients with castration-resistant PCa (CRPC). However, the widespread use of ARPIs has been accompanied by the emergence of a more aggressive subtype, treatment-induced neuroendocrine PCa (t-NEPC). t-NEPC is characterized by small-cell carcinoma–like morphology, including indistinct cell borders, a high nuclear-to-cytoplasmic ratio, hyperchromatic nuclei, and elevated expression of neuroendocrine markers. While de novo NEPC is exceedingly rare, t-NEPC accounts for approximately 20–25% of advanced CRPC cases. Clinically, t-NEPC is highly proliferative, rapidly progressive, and largely independent of AR signaling, leaving patients with limited treatment options. Platinum-based chemotherapy remains the mainstay, yet prognosis is dismal, with median survival often less than one year. These challenges underscore the urgent need to elucidate the molecular mechanisms underlying treatment-induced neuroendocrine differentiation (t-NED), with the ultimate goal of identifying actionable targets and developing more effective therapeutic strategies.

Histologically, small-cell neuroendocrine carcinoma represents a distinct subset of clinical CRPC and belongs to the histological variant forms of PCa, characterized by the loss of luminal differentiation [4–6]. The cellular origin of NEPC remains debated: one hypothesis posits expansion of pre-existing normal NE cells under ADT, whereas an alternative model suggests that therapeutic pressure promotes lineage plasticity, whereby genetic and epigenetic alterations drive trans-differentiation of adenocarcinoma into NE carcinoma [7–9]. Genomic alterations commonly associated with NEPC include concurrent loss of TP53, RB1, and PTEN, which are also frequent in treatment-emergent cases [10–13]. In addition, oncogenic regulators such as MYCN, AURKA, ONECUT2, ASCL1, MUC1-C, and PEG10 have been identified as critical drivers of the NE phenotype, highlighting them as potential therapeutic targets across NEPC and other neuroendocrine cancers [14–18]. Importantly, three interconnected biological states—epithelial mesenchymal transition (EMT), stemness, and NED—converge through shared networks of transcription factors, epigenetic regulators, and cell surface receptors to orchestrate the progression of PCa toward a neuroendocrine lineage [14, 19, 20]. Despite these advances, the precise molecular circuitry governing the initiation and progression of NEPC remains incompletely defined, underscoring the urgent need for deeper mechanistic insight to inform therapeutic development.

OPRK1 encodes a G-protein-coupled opioid receptor broadly distributed in the nervous system and known to participate in arousal and neuroendocrine regulation [21–23]. Its expression has been reported to increase in CRPC [24]. In our study, we established cellular models recapitulating lineage plasticity and conducted transcriptomic profiling, through which OPRK1 emerged as one of the consistently upregulated genes during NED. These findings extend prior observations by implicating OPRK1 not only in CRPC but also in lineage switching processes. Importantly, the contribution of OPRK1 to the development of NEPC—a highly lethal variant of advanced disease with limited therapeutic options—remains largely unexplored. Clarifying how OPRK1 drives neuroendocrine features and therapy resistance is therefore both mechanistically plausible and of substantial clinical significance.

In this study, we combined bioinformatics, transcriptomic profiling, and both in vitro and in vivo functional assays to dissect the role of OPRK1 in driving lineage plasticity and NED of PCa cells. By integrating multi-level evidence, our work uncovers mechanistic insights into OPRK1-mediated reprogramming and provides a rationale for developing OPRK1-targeted strategies to overcome treatment-induced NED.

## Result

### OPRK1 expression is upregulated in t-NEPC

**Fig. 1:**
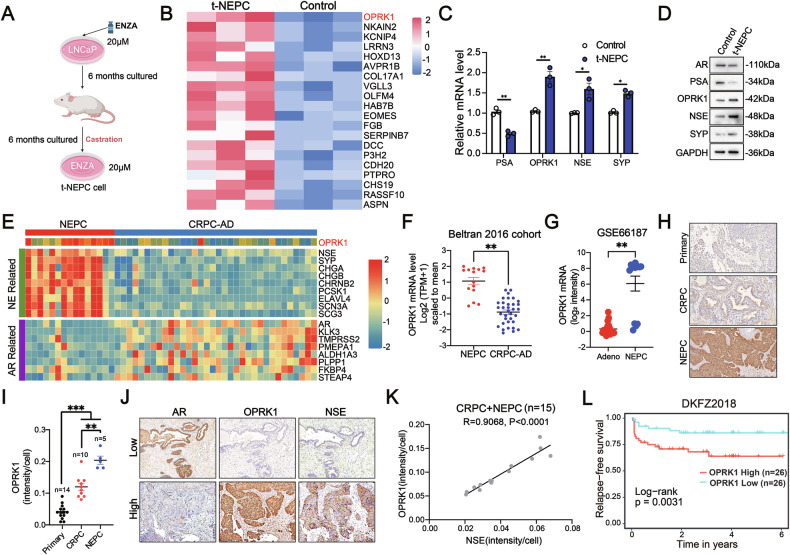
OPRK1 is highly upregulated in neuroendocrine prostate cancer (NEPC) and predicts poor clinical outcome. **A** Schematic showing establishment of treatment-induced NEPC (t-NEPC) from LNCaP cells through long-term ENZA exposure and castration. **B** Heatmap of the top 20 differentially expressed genes between t-NEPC (*n* = 3) and control LNCaP cells (*n* = 3). **C** qRT-PCR validation of *PSA*, *OPRK1, NSE* and *SYP* expression in t-NEPC and control LNCaP cells. *GAPDH* served as an internal control. **D** Immunoblots of AR, PSA, OPRK1, NSE and SYP in t-NEPC and control LNCaP cells, with GAPDH as a loading control. **E** Heatmap of OPRK1 and selected NE- and AR-associated genes in NEPC (*n* = 15) and CRPC-adenocarcinoma (CRPC-AD, *n* = 34) from the Beltran 2016 cohort. **F** *OPRK1* mRNA levels are significantly elevated in NEPC versus CRPC-AD (Beltran 2016). **G** *OPRK1* expression is upregulated in NEPC compared to adenocarcinoma samples from the GSE66187 dataset. **H**–**I** Representative immunohistochemistry (IHC) images and quantification of OPRK1 in primary PCa (*n* = 14), CRPC (*n* = 10), and NEPC (*n* = 5). Scale bar: 250 μm. **J**, **K** IHC staining of AR, OPRK1, and the NE marker NSE in CRPC (*n* = 10) and NEPC (*n* = 5), with Pearson’s correlation analysis between OPRK1 and NSE intensities. Scale bar: 50 μm. **L** Kaplan-Meier analysis showing high OPRK1 expression is associated with significantly shorter relapse-free survival in the DKFZ2018 cohort. Data are presented as mean ± SEM. **p < 0.01; ***p < 0.001.

t-NEPC represents a highly aggressive subtype of CRPC whose incidence is rising with the widespread clinical use of potent ARPIs such as ENZA. To model this process, we generated a t-NEPC cell line by chronically exposing AR⁺ LNCaP cells to ENZA (Fig. 1A). The resulting cells displayed AR downregulation, acquisition of NE-like features, and ENZA resistance (Fig. S1A–C). RNA-seq profiling of t-NEPC versus parental LNCaP cells revealed marked transcriptional reprogramming, with OPRK1 emerging as one of the most significantly upregulated genes (Fig. 1B). Both mRNA and protein analyses confirmed OPRK1 over-expression in t-NEPC cells (Fig. 1C, D), implicating it as a candidate driver of treatment-induced lineage plasticity.

We next interrogated clinical datasets to assess OPRK1 relevance in patient tumors. Analysis of the Beltran 2016 cohort [11] demonstrated significantly higher *OPRK1* mRNA in NEPC compared with CRPC-adenocarcinoma (CRPC-AD) (Fig. 1E, F). Independent validation using GSE66187 confirmed elevated *OPRK1* expression in NEPC relative to adenocarcinoma (Fig. 1G). Immunohistochemical staining of our CRPC tissue cohort further demonstrated progressively increased OPRK1 expression from primary PCa to CRPC and NEPC (Fig. 1H, I). Importantly, OPRK1 protein levels positively correlated with the NE marker NSE in CRPC samples (Fig. 1J, K), and *OPRK1* mRNA expression showed strong correlations with NE biomarkers (*NCAM2* and *CGA*) across five independent prostate cancer cohorts (SU2/PCF Dream Team, 2019 [25], SU2/PCF Dream Team, 2015 [26], MSK, 2010 [27], Fred Hutchinson CRC 2016 [28] and MSK 2022) (Supplementary Fig. 1D). Moreover, GSEA revealed that tumors with high *OPRK1* expression displayed transcriptomic signatures resembling NEPC (represented by 180 under-expressed genes and 285 over-expressed genes in NEPC) [11, 29, 30] (Supplementary Fig. 1E).

Consistent with these findings, *OPRK1* expression was lowest in AR⁺/NE^−^ PCa lines (LNCaP, VCaP, 22RV1), intermediate in AR⁻/NE-like cells (PC3, DU145), and highest in bona fide NEPC cells (NCI-H660) (Supplementary Fig. 1F). GSEA of the t-NEPC RNA-seq data further demonstrated enrichment of neurotrophin signaling, EMT, and OPRK1-related targets, suggesting a role for OPRK1 in regulating cellular plasticity (Supplementary Fig. 1G). The OPRK1 targets were defined as the top 100 differentially expressed genes in RNA seq data between the t-NEPC cell with OPRK1-knockdown and the control (Supplementary Table 1). In ENZA-resistant C4-2B cells (GSE159548), we also observed increased *OPRK1* expression together with down-regulation of luminal genes (*KLK2, KLK3*) and upregulation of NE markers (*NCAM1, NSE, CHGA*) (Supplementary Fig. 1H). Finally, meta-analysis of multiple external datasets (DKFZ2018, TCGA-PRAD, GSE21034, GSE116918) linked high *OPRK1* expression with adverse clinical features, including advanced T stage, higher Gleason scores, increased metastatic potential, and shorter survival (Fig. 1L, Supplementary Fig. 1I–K).

In summary, both experimental and clinical evidence converge to show that OPRK1 is consistently elevated in NEPC, correlates with NE features, and is associated with poor prognosis, highlighting its potential as a mechanistic driver and therapeutic target in t-NEPC.

### OPRK1 shows a negative correlation with AR signaling and is suppressed by AR in CRPC

**Fig. 2:**
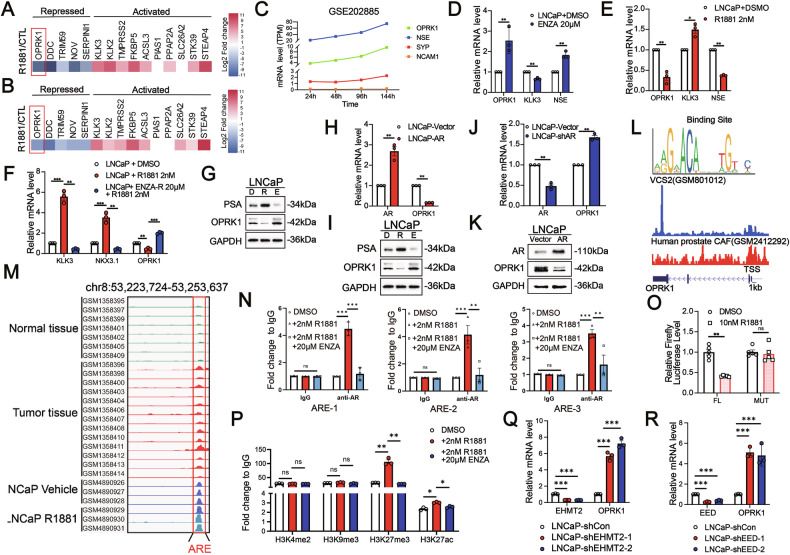
AR directly represses OPRK1 transcription in PCa cells. **A**, **B** Heatmaps of AR-regulated genes in LNCaP and VCaP cells following androgen (R1881) stimulation, analyzed from the GSE71797 dataset. **C** Time-course RNA-seq (GSE202885) showing increased OPRK1 and NE markers (NSE, SYP, NCAM1) in 16D-CRPC cells cultured with ENZA. **D**, **E** qPCR analysis of OPRK1, KLK3, and NSE after short-term ENZA (20 μM, 6 h) or R1881 (2 nM, 6 h) treatment in LNCaP cells. **F**, **G** q-PCR (left panel) and Immunoblots (right panel) of indicated genes (OPRK1, KLK3, NKX3.1 and PSA) in LNCaP cells treated with R1881(20 μM, 7days) with or without ENZA (10 nM, 7days). **H**, **I** q-PCR (upper panel) and immunoblots ((lower panel) of AR and OPRK1 in LNCaP cells stably AR over-expressing versus control. **J**, **K** q-PCR (upper panel) and immunoblots ((lower panel) of AR and OPRK1 in AR knockdown versus control LNCaP cells. **L** Schematic diagram represents AR binding motif (upper panel), AR binding peaks visualization from the CistromeDB database (middle panel), and androgen response elements (ARE) (lower panel) located in the OPRK1 promoter. **M** Genomic browser representation of AR binding in OPRK1 promoter encompassing AREs in the GSE161167 (LNCaP cells) and GSE70079 (a cohort of normal and tumor human prostate tissues) datasets. **N** ChIP-qPCR confirming AR enrichment at OPRK1 promoter AREs in LNCaP cells upon R1881 or ENZA treatment. **O** Luciferase reporter assays with wild-type or ARE-mutant OPRK1 promoter constructs in LNCaP cells, showing AR-dependent transcriptional repression. **P** ChIP-qPCR detecting changes of enrichment of H3K4me2, H3K9me3, H3K27me3, and H3K27ac on the OPRK1 promoter in androgen R1881 (2 nM) stimulated or ENZA (20 µM) treated LNCaP cells. **Q**, **R** qPCR showing OPRK1 up-regulation following shRNA knockdown of EHMT2 or EED in LNCaP cells. Data are presented ad mean ± SEM. **p < 0.01; ***p < 0.001.

Given that t-NEPC frequently emerges after potent AR pathway inhibition, we next explored the regulatory relationship between OPRK1 and AR signaling. Analysis of the GSE71797 dataset, in which LNCaP and VCaP cells were stimulated with synthetic androgen R1881, revealed a marked suppression of OPRK1 expression upon AR activation (Fig. 2A, B). Conversely, RNA-seq data from 16D-CRPC cells treated with ENZA (GSE202885) demonstrated a time-dependent increase in OPRK1 and NE marker transcripts (*NSE, SYP, NCAM1*) following AR blockade (Fig. 2C). These results were validated experimentally: short-term (12 h) ENZA treatment of AR⁺ LNCaP/VCaP cells significantly induced *OPRK1* and *NSE* while reducing *KLK3*, whereas R1881 stimulation had the opposite effect (Fig. 2D, E and Supplementary Fig. 2A, B). Prolonged AR activation (R1881 for 7 days) further reinforced this inverse relationship, with robust up-regulation of AR targets (KLK3, NKX3.1) and concomitant suppression of OPRK1 at both mRNA and protein levels. In contrast, sustained ENZA treatment (7 days) resulted in strong OPRK1 induction and down-regulation of AR-driven genes (Fig. 2F, G and Supplementary Fig. 2C, D). Direct genetic modulation of AR yielded consistent outcomes: forced AR expression decreased OPRK1, whereas AR knockdown significantly upregulated OPRK1 (Fig. 2H, K and Supplementary Fig. 2E–H).

To dissect the mechanism of repression, we examined the OPRK1 promoter for AR binding motifs using JASPAR and identified three putative androgen response elements (AREs) at +1879 to +1893 bp, −1821 to −1835 bp, and −1881 to −1895 bp. Public cistromeDB data confirmed AR occupancy at these sites in PCa cells and fibroblasts (Fig. 2L), and clinical ChIP-seq analyses showed higher AR binding at the OPRK1 promoter in tumors relative to normal prostate tissue (Fig. 2M). ChIP-qPCR validated these findings: AR recruitment to the three AREs was strongly enhanced by R1881 and diminished by ENZA in LNCaP cells (Fig. 2N). Promoter luciferase assays further demonstrated that AR activation significantly repressed OPRK1 promoter activity. We cloned the OPRK1 promoter region (−1987 to +1991 bp) into a luciferase reporter construct and found that R1881 stimulation markedly reduced reporter activity in LNCaP cells. This repression was completely abolished when ARE-directed mutations were introduced into the OPRK1 promoter, confirming that AR directly suppresses OPRK1 transcription through these cis-regulatory elements (Fig. 2O).

Because histone modifications often cooperate with AR in transcriptional regulation, we investigated epigenetic changes at the OPRK1 promoter [31, 32]. Among several histone marks, H3K27me3 (a repressive histone modification) showed the most prominent dynamic changes in response to AR signaling among these epigenetic modifications (Fig. 2P). To find which H3K27 methylation writer was involved in the *OPRK1* transcriptional regulation, we analyzed the expression of common histone methyltransferases at H3K27 in PCa by combining data from the Beltran cohort and CCLE. EHMT2 and PRC2 complex (EED, EZH2 and SUZ12) showed the highest expression in PCa cell lines (Supplementary Fig. 2I). They may be the potential roles in *OPRK1* transcriptional modulation by AR. Functional perturbation experiments revealed that knockdown of EHMT2 or EED, but not EZH2 or SUZ12, significantly increased OPRK1 transcription (Fig. 2Q, R and Supplementary Fig. 2J,K).

In summary, these results demonstrate that AR directly binds to the OPRK1 promoter and represses its transcription through AREs and H3K27me3 deposition, with EHMT2/EED contributing to this repression. Potent AR inhibition by ENZA disrupts this regulatory axis, relieving transcriptional suppression and driving OPRK1 up-regulation.

### OPRK1 is essential to maintain the NE characteristics and malignancy of NEPC cells

**Fig. 3:**
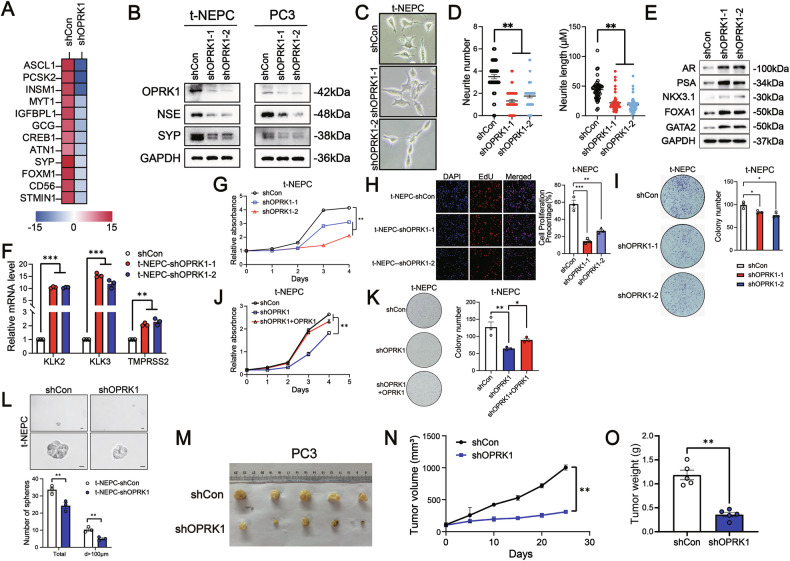
OPRK1 knockdown attenuates neuroendocrine features, proliferative capacity, and tumorigenicity of NEPC cells. **A** Heatmap showing NE-associated genes downregulated in t-NEPC cells upon OPRK1 silencing (RNA-seq). Expression changes are displayed on a log₂ fold-change scale. **B** Immunoblots showing efficient OPRK1 silencing and reduced expression of canonical NE markers (NSE, SYP) in t-NEPC and PC3 cells. **C**, **D** Representative morphology and quantification of neurite number and average neurite length in control versus OPRK1-depleted t-NEPC cells (≥0 cells/group), as a representative of three independent experiments. **E** Immunoblots of AR signaling and luminal lineage factors (AR, PSA, NKX3.1, FOXA1, GATA2) in t-NEPC cells after OPRK1 knockdown. **F** qPCR analysis of AR target genes (KLK2, KLK3, TMPRSS2) in OPRK1-silenced versus control t-NEPC cells. **G**, **H** CCK8 and EdU proliferation assays demonstrating impaired growth upon OPRK1 knockdown (*n* = 3). **I** Representative images and quantification of colony formation demonstrating reduced clonogenicity in OPRK1-silenced t-NEPC cells (*n* = 3). Rescue assays: re-expression of OPRK1 in knockdown t-NEPC cells restores proliferation (CCK-8, **J**) and colony formation capacity (**K**), confirming the specificity of OPRK1 function (*n* = 3). **L** Tumorsphere assays demonstrating reduced stem-like capacity in OPRK1-silenced t-NEPC cells (*n* = 3). Subcutaneous xenografts of PC3 cells stably expressing shOPRK1 or shControl: representative tumors (**M**), tumor growth curves (**N**), and final tumor weights (**O**) (*n* = 5 per group). Data are mean ± SEM; two-tailed tests unless indicated; *p < 0.05； **p < 0.01; ***p < 0.001.

To assess the role of OPRK1 in sustaining the NE phenotype of PCa cells, we generated stable OPRK1 knockdown lines using two independent shRNAs in t-NEPC cells and PC3 cells, the latter of which displays partial NE features [33]. RNA-seq analysis revealed that depletion of OPRK1 in t-NEPC cells led to a marked downregulation of NE-associated gene expression compared with control cells (Fig. 3A and Supplementary Table 2). Consistently, western blotting confirmed that OPRK1 knockdown reduced protein levels of canonical NE markers, including NSE and SYP, in both t-NEPC and PC3 cells (Fig. 3B). We next examined the impact of OPRK1 silencing on cell morphology. Loss of OPRK1 resulted in a de-differentiated phenotype characterized by fewer neurite extensions and a significant reduction in average neurite length per cell, indicating attenuation of NE-like morphology in t-NEPC cells (Fig. 3C, D). Notably, suppression of OPRK1 reprogrammed t-NEPC cells from an NE lineage back toward an AR+ luminal lineage. This phenotypic switch was supported by restoration of AR signaling, evidenced by increased expression of AR, PSA, and chromatin pioneer factors FOXA1 and GATA2, along with robust induction of classical AR target genes such as PSA, NKX3.1 and TMPRSS2 (Fig. 3E, F). Collectively, these findings demonstrate that OPRK1 is essential for maintaining NE differentiation in PCa cells, and its loss induces a lineage reversion toward the AR-driven luminal state.

NEPC is clinically defined by highly proliferative growth and rapid progression. Given this hallmark feature, we examined whether OPRK1 depletion alters the growth advantage of NEPC cells. Silencing OPRK1 significantly suppressed proliferation (CCK8 and EdU) and anchorage-independent colony formation in t-NEPC and PC3 cells (Fig. 3G-I and Supplementary Fig. 3A-C), whereas re-expression of OPRK1 restored these malignant phenotypes (Fig. 3J, K). Similar inhibitory effects were observed in additional NE-like cell lines (22RV1 and DU145) (Supplementary Fig. 3D-3E). Because NEPC often emerges through the acquisition of stem-like traits under androgen deprivation, we next tested the role of OPRK1 in stemness [34]. Knockdown markedly reduced tumor-sphere formation in t-NEPC and PC3 cells (Fig. 3L and Supplementary Fig. 3F). In vivo xenograft models, OPRK1 loss significantly impaired tumor growth, as reflected by reduced tumor volume and weight (Fig. 3M-O). Together, these findings demonstrate that OPRK1 is essential for sustaining NEPC proliferation, stemness, and malignant progression.

In summary, these findings identify OPRK1 as a critical regulator of NE differentiation, stemness, and tumor aggressiveness in PCa.

### OPRK1 represses AR signaling and significantly promotes t-NED in PCa

**Fig. 4:**
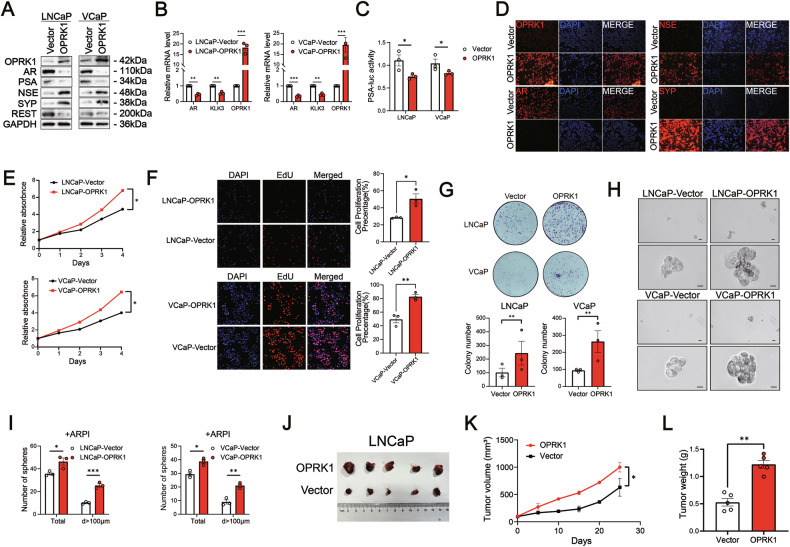
OPRK1 over-expression promotes NE plasticity and enhances proliferative and stem-like traits of PCa cells. **A** Immunoblot analysis showing decreased AR and PSA, and increased NSE, SYP, and REST down-regulation in OPRK1-overexpressing LNCaP and VCaP cells. **B** qPCR confirming suppression of AR and KLK3 expression upon OPRK1 overexpression (n = 3). **C** PSA-luciferase reporter assays showing reduced AR transcriptional activity in OPRK1-overexpressing cells (*n* = 3). **D** Immunofluorescence staining validating increased NE marker expression (NSE, SYP) and loss of AR in OPRK1-overexpressing LNCaP cells. **E**, **F** CCK8 and EdU proliferation assays showing accelerated growth of OPRK1-overexpressing LNCaP and VCaP cells compared with vector controls (*n* = 3). **G** Colony formation assays demonstrating enhanced clonogenicity upon OPRK1 over-expression (*n* = 3). **H**, **I** Tumorsphere assays indicating increased self-renewal capacity in OPRK1-overexpressing LNCaP and VCaP cells, particularly under ARPI conditions (*n* = 3). **J** Representative xenograft images from castrated NSG mice implanted with LNCaP-OPRK1 or vector control cells (*n* = 5). **K, L** Quantification of tumor growth curves and final tumor weights showing that OPRK1 promotes robust castration-resistant tumor growth in vivo. Data are presented as mean ± SEM. *p < 0.05; **p < 0.01; ***p < 0.001.

To determine whether OPRK1 can actively induce a NE phenotype in PCa cells, we established stable OPRK1-overexpressing lines in AR+ LNCaP and VCaP cells. Compared with controls, OPRK1 over-expression markedly suppressed AR and PSA protein levels in both lines (Fig. 4A). Consistent with this effect, OPRK1 over-expression reduced AR and AR target gene (*KLK3*) expression, and significantly diminished AR-dependent transcriptional activity as measured by a PSA-luciferase reporter assay (Fig. 4B, C). Concomitantly, OPRK1 over-expression increased the protein levels of canonical NE markers, including NSE, SYP, and REST, as shown by western blotting (Fig. 4A). Immunofluorescence staining further confirmed elevated NSE and SYP expression in OPRK1-overexpressing LNCaP cells compared with controls (Fig. 4D). Similar results were obtained in C4-2 cells, where OPRK1 over-expression repressed AR signaling while concomitantly enhancing NE marker expression (Supplementary Fig. 4A, B). Collectively, these findings indicate that OPRK1 is sufficient to repress AR signaling and drive NE-like differentiation in PCa cells.

To evaluate the effect of OPRK1 activation on the proliferative capacity and stem-like properties of PCa cells, we over-expressed OPRK1 in LNCaP, VCaP and C4-2 lines. Compared with controls, enforced OPRK1 expression markedly enhanced cell proliferation (CCK8 and EdU), colony-forming ability, and tumor-sphere formation (Fig. 4E, I and Supplementary Fig. 4C-4E). To further assess its role in castration resistance in vivo, OPRK1-overexpressing or control LNCaP cells were subcutaneously implanted into castrated NSG mice (Fig. 4J). Consistent with in vitro findings, OPRK1-overexpressing xenografts displayed robust castration-independent growth, in stark contrast to the minimal tumor development observed in the control group (Fig. 4K, L). These results demonstrate that OPRK1 promotes proliferative and stemness-associated traits in PCa cells and drives lineage plasticity, thereby facilitating the transition from AR⁺ PCa toward an NEPC-like state.

**Fig. 5:**
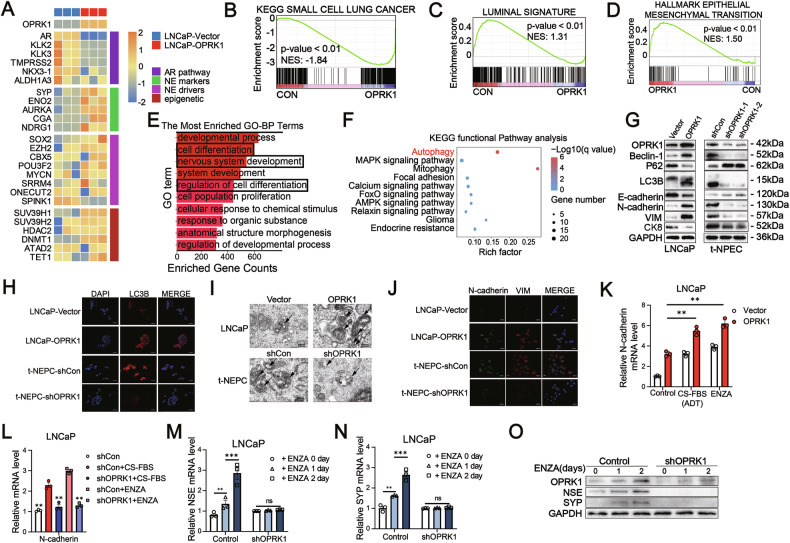
OPRK1 drives the neuroendocrine differentiation of PCa. **A** Heatmap depicting OPRK1 and selected genes expression in stable OPRK1 over-expressing LNCaP (*n* = 3) and control cells (*n* = 3) (mRNA expression value=log_2_(TPM + 1)). **B**–**D** Gene Set Enrichment Analysis (GSEA) of small cell lung cancer signature, luminal cell gene signature and epithelial-mesenchymal transition. **E** The top enriched processes in gene ontology (GO) enrichment analysis of RNA-Seq data from LNCaP-OPRK1 and control cells. The length of bar represents the number of enriched genes. **F** KEGG functional pathway analyses of OPRK1 target genes. **G** Immunoblots of OPRK1, autophagy markers (Beclin-1, P62 and LC3B) andEMT markers (E-cadherin, N-cadherin, VIM and CK8) in OPRK1 over-expressing LNCaP/control cells and OPRK1 knockdown t-NEPC/control cell. **H** The expression and location of LC3B markers in LNCaP-Vector/OPRK1 and t-NEPC-shCon/shOPRK1 cells were analyzed by immunofluorescence staining. **I** Electron microscopy images showing lysosomes and autophagosomes within LNCaP-Vector/OPRK1 and t-NEPC-shCon/shOPRK1 cells. **J** The expression and location of vimentin (Vim) and N-cadherin (N-cad) markers in LNCaP-Vector/OPRK1 and t-NEPC-shCon/shOPRK1 cells were analyzed by immunofluorescence staining. **K** qPCR analyses were used to examine N-cadherin expression in LNCaP cells with and without over-expression of OPRK1 under ADT (CS-FBS) or ENZA treatment. **L** qPCR analyses were used to examine N-cadherin expression in LNCaP cells with and without knockout of OPRK1 under CS-FBS or ENZA treatment. **M**, **N** qPCR analysis of NSE and SYP mRNA levels of shOPRK1 and control LNCaP cells cultured with 40 μM ENZA for indicated time. **O** Immunoblots of NSE, SYP and OPRK1 in samples described in (**M**, **N**). Data represent the mean ± SEM. *p < 0.05; **p < 0.01; ***p < 0.001.

We next performed RNA sequencing to compare transcriptomic profiles between LNCaP-OPRK1 and control LNCaP cells. In line with our phenotypic observations, enforced OPRK1 expression led to a robust up-regulation of NE-associated genes (SYP, NSE, AURKA, CGA, NDRG1), while genes associated with AR signaling (AR, KLK2, KLK3, TMPRSS2, NKX3-1, ALDH1A3) were significantly down-regulated (Fig. 5A). GSEA further revealed enrichment of neuronal differentiation–related signatures (e.g., neuron fate commitment, neuropilin binding) and the small cell lung cancer gene set in OPRK1-overexpressing cells (Supplementary Fig. 5A, B and Fig. 5B). Conversely, luminal lineage and epithelial–mesenchymal transition (EMT) gene signatures were suppressed (Fig. 5C, D). Functional annotation of differentially expressed genes (DEGs) using GO and KEGG confirmed significant enrichment in pathways linked to “cell differentiation”, “nervous system development”, “regulation of cell fate” and “autophagy” (Fig. 5E, F). At the protein level, western blot and immunostaining validated increased NE markers (NSE, SYP), altered autophagy markers (Beclin-1, p62 and LC3B), EMT-associated shifts (decreased E-cadherin, increased N-cadherin and Vimentin), and down-regulation of the luminal marker CK8 in both LNCaP and VCaP OPRK1-overexpressing cells (Figs. 4A, 5G and Supplementary Fig. 5C, D, G).

Silencing OPRK1 in t-NEPC cells led to a marked reduction in the expression of classical NE markers, inhibition of autophagy and reversal of EMT (Figs. 3A, 5G and Supplementary Fig. 5E, F, G). These molecular changes were corroborated by immunofluorescence and electron microscopy, which provided direct visualization consistent with the qPCR and western blot results (Fig. 5H-J and Supplementary Fig. 5H-I). Collectively, these findings implicate OPRK1 as a key regulator of autophagy and cellular plasticity, thereby facilitating NEPC development. To further establish the role of OPRK1 in treatment-induced lineage plasticity, we examined its interaction with AR pathway inhibition. Over-expression of OPRK1 potentiated ARPI-induced EMT in LNCaP and VCaP cells (Fig. 5K and Supplementary Fig. 5J), whereas OPRK1 knockdown blunted this effect (Fig. 5L and Supplementary Fig. 5K). Moreover, shRNA-mediated depletion of OPRK1 abrogated the ENZA-induced upregulation of NE markers in LNCaP cells (Fig. 5M-O). Transcriptomic analysis supported these observations: qPCR and GSEA of RNA-seq data demonstrated a significant down-regulation of AR signaling in OPRK1-overexpressing LNCaP cells (Supplementary Fig. 5L-N), highlighting a reciprocal antagonism between OPRK1 and AR signaling. Together, these results indicate that OPRK1 drives lineage plasticity by coupling EMT and autophagy activation, thereby enabling PCa cells to transition toward an NEPC state under AR pathway suppression.

### OPRK1–SLC9A3R1 signaling promotes autophagy-mediated REST degradation to drive NEPC plasticity

**Fig. 6:**
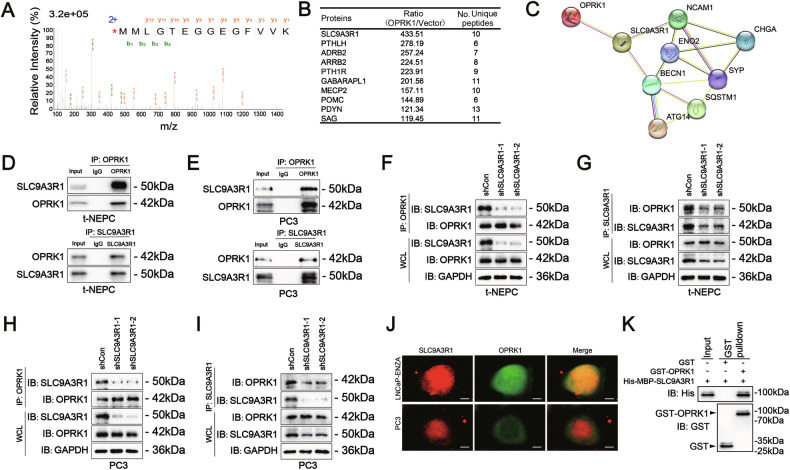
OPRK1 interacts with SLC9A3R1 in PCa cells. **A**, **B** Mass spectrometry and proteomic profiling identified SLC9A3R1 as the top-ranking OPRK1-interacting protein. **C** STRING analysis revealed SLC9A3R1 as a central hub linking OPRK1 to NE and autophagy-associated markers. **D**, **E** Reciprocal co-immunoprecipitation (co-IP) confirmed the endogenous interaction between OPRK1 and SLC9A3R1 in t-NEPC and PC3 cells. **F, G** Knockdown of SLC9A3R1 disrupted the OPRK1-SLC9A3R1 interaction as shown by co-IP assays. **H**, **I** Silencing SLC9A3R1 suppressed OPRK1-induced NE marker expression, autophagy activation, and EMT signatures in both t-NEPC cells and OPRK1-overexpressing LNCaP cells. **J** Immunofluorescence staining demonstrated co-localization of OPRK1 and SLC9A3R1 in t-NEPC and PC3 cells. **K** GST pull-down assay using purified proteins verified a direct physical interaction between OPRK1 and SLC9A3R1.

Accumulating evidence indicates that NEPC homeostasis critically depends on autophagy [35–39]. Building on our findings that OPRK1 induces lineage plasticity and NED through autophagy activation, we next sought to identify the molecular intermediates. Label-free quantitative proteomics of OPRK1-overexpressing cells identified SLC9A3R1 as the top-ranking OPRK1-interacting candidate (Fig. 6A, B). STRING network analysis placed SLC9A3R1 at the center of an interaction hub connecting OPRK1 with multiple NE and autophagy-associated markers (Fig. 6C). Reciprocal co-IP assays confirmed the endogenous interaction between OPRK1 and SLC9A3R1 in t-NEPC and PC3 cells, which was abolished upon SLC9A3R1 knockdown (Fig. 6D-I). Immunofluorescence further demonstrated co-localization of OPRK1 with SLC9A3R1 (Fig. 6J). GST pull-down assays using purified recombinant proteins verified a direct physical interaction (Fig. 6K).

**Fig. 7:**
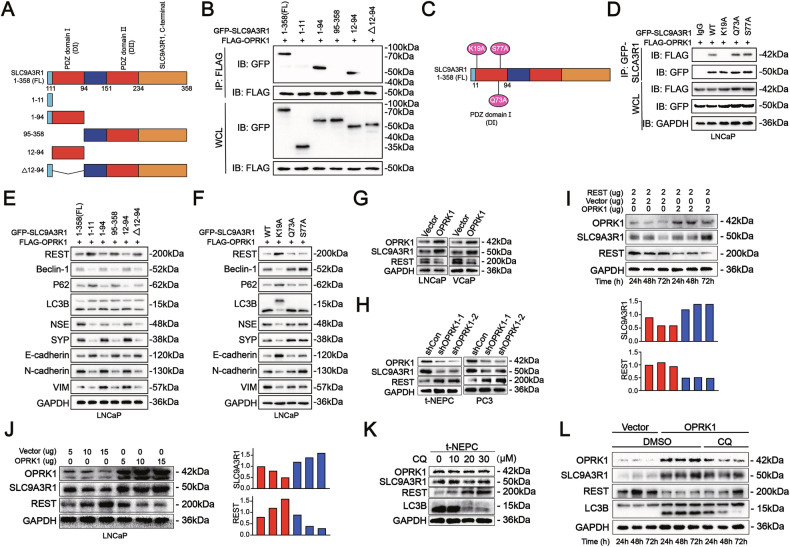
OPRK1-SLC9A3R1 interaction drives REST degradation and NE plasticity in PCa. **A** Schematic representation of full-length (FL) SLC9A3R1 and truncation mutants used for domain mapping. **B** Co-IP assays in HEK293T cells co-expressing FLAG-OPRK1 and different GFP-SLC9A3R1 truncations revealed that the PDZ I domain (12-94aa) is essential for OPRK1-SLC9A3R1 binding. **C** Structural modeling identified three potential interacting residues (K19, Q73, S77) in the PDZ I domain. **D** Co-IP validation confirmed that point mutation K19A disrupted OPRK1–SLC9A3R1 interaction, whereas Q73A and S77A retained binding. **E** Immunoblots showed that only SLC9A3R1 constructs containing the PDZ I domain enabled OPRK1-mediated REST degradation, autophagy activation (Beclin-1, p62, LC3B), induction of NE markers (NSE, SYP), and EMT markers (N-cadherin, Vimentin), with concomitant loss of epithelial marker E-cadherin. **F** Similarly, PDZ I point-mutation analysis demonstrated that K19A abolished OPRK1-driven REST degradation and associated NE/EMT phenotypes. **G**, **H** Immunoblots in LNCaP, VCaP, t-NEPC, and PC3 cells confirmed OPRK1-SLC9A3R1 interaction is required for REST destabilization and downstream reprogramming. **I** Cycloheximide-chase assays showed accelerated degradation of REST in the presence of OPRK1, whereas SLC9A3R1 depletion prevented this effect. **J** Dose-dependent increase of OPRK1 in LNCaP cells progressively reduced REST protein levels, correlating with up-regulation of SLC9A3R1. **K**, **L** CQ-mediated autophagy inhibition restored REST stability in both t-NEPC and OPRK1-overexpressing LNCaP cells, confirming that OPRK1-SLC9A3R1 promotes REST degradation through an autophagy-dependent mechanism.

To delineate the binding interface, we combined molecular docking with 3D structural modeling from the RCSB database (https://www.rcsb.org/), which predicted a highly stable interaction (binding energy: −296.41 kcal/mol) mediated through the PDZ I domain (12-94 aa) of SLC9A3R1 (Supplementary Fig. 6A). Truncation and point-mutation analysis further confirmed that residue K19 within the PDZ I domain is indispensable for OPRK1-SLC9A3R1 binding (Fig. 7A-D and Supplementary Fig. 6B). Consistently, functional assays showed that only SLC9A3R1 truncation mutants retaining the PDZ I domain supported OPRK1-driven phenotypes. Co-expression of Flag-OPRK1 with GFP-SLC9A3R1 constructs in LNCaP/VCaP cells demonstrated that PDZ I-containing mutants enabled REST autophagic degradation and promoted NED/EMT phenotypes, whereas mutants lacking PDZ I failed to do so (Fig. 7E, Supplementary Fig. 6C). Moreover, the K19A mutation abolished OPRK1-SLC9A3R1 binding and blocked REST degradation, thereby suppressing OPRK1-induced lineage plasticity (Fig. 7F, Supplementary Fig. 6D). Together, these data establish that the PDZ I domain of SLC9A3R1 is essential for mediating OPRK1-driven autophagy and phenotypic reprogramming.

Having established that OPRK1 directly engages SLC9A3R1 to activate autophagy, we next examined the downstream consequence of this signaling axis. REST downregulation is a molecular hallmark of NEPC [40–42]. Although REST mRNA levels remained unchanged upon OPRK1 over-expression or knockdown, REST protein was markedly reduced in LNCaP/VCaP-OPRK1 cells and restored in t-NEPC/PC3 cells following OPRK1 silencing (Fig. 7G, H, Supplementary Fig. 6E, F), indicating post-translational regulation. OPRK1 overexpression accelerated REST protein turnover in 293 T cells (Fig. 7I) and promoted a dose-dependent reduction of endogenous REST levels in LNCaP cells (Fig. 7J). Importantly, inhibition of autophagy with chloroquine (CQ) blocked OPRK1-induced REST degradation without affecting OPRK1 or SLC9A3R1 levels (Fig. 7K, L, Supplementary Fig. 6G). Moreover, SLC9A3R1 knockdown phenocopied the restoration of REST protein (Supplementary Fig. 6H, I).

Collectively, these findings define a mechanistic pathway in which OPRK1 binds SLC9A3R1 via its PDZ I domain to activate autophagy, which destabilizes REST protein and relieves its repressive function. The resulting loss of REST drives neuroendocrine trans-differentiation and EMT, thereby fueling lineage plasticity and NEPC progression.

### JTC-801 combined with CQ inhibits OPRK1 to suppress cellular autophagy, NED, and EMT in NEPC

**Fig. 8:**
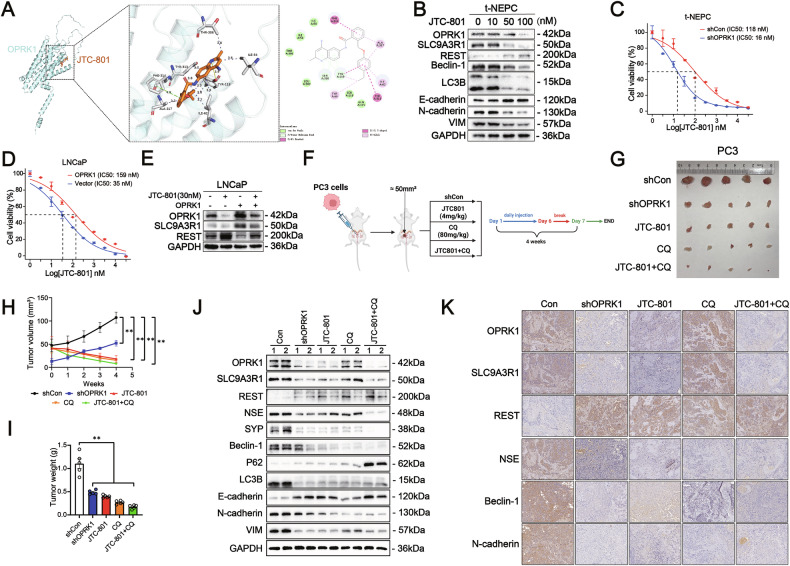
JTC-801 combined with CQ suppresses cellular autophagy, NED, and EMT ofNEPC via inhibiting OPRK1/autophagy axis. **A** The 3D and 2D docking diagram of the interaction between OPRK1 protein and JTC-801. **B** t-NEPC Cells treated with JTC-801 (0 nM, 10 nM, 50 nM, 100 nM) and collected 48 h later for detection of OPRK1 by immunoblotting analysis. **C** t-NEPC Cells were treated with JTC-801 alone or along with OPRK1 knockdown plasmids for 48 h. The IC50 of JTC-801 was detected by CCK-8 assay. **D** t-NEPC Cells were treated with JTC-801 alone or along with OPRK1 over-expressing plasmids for 48 h. The IC50 of JTC-801 was detected by CCK-8 assay. **E** LNCaP transfected with JTC-801 alone or along with OPRK1 over-expression plasmid were collected 48 h later for detection of OPRK1, SLC9A3R1 and REST by immunoblotting assay. **F** Schematic illustration of the in vivo JTC-801 experimental design. Male BALB/c nude mice (*n* = 5) bearing PC3 or PC3 transfected with OPRK1 knockdown cells were intraperitoneally treated with JTC801 (4 mg/kg) or CQ (80 mg/kg) or JTC801(4 mg/kg)+CQ (80 mg/kg) daily for 6 days followed by a one-day break for a period of 4 weeks, then tumors were harvested for analysis. **G** Photos of the formed tumors at the end of experiments. **H** Tumor growth curve. **I** Tumor weight at the end of experiments. **J** The formed tumors were collected for detection of OPRK1, SLC9A3R1, REST, NE markers, autophagy markers and EMT markers by immunoblotting assay. **K** Representative IHC staining of OPRK1, SLC9A3R1, REST, NSE, SYP, Beclin-1, P62, LC3B, E-cadherin, N-cadherin and VIM in tumor samples. Scale bars: 200 μm.

The above findings highlight the pivotal role of OPRK1 in NEPC progression and suggest that pharmacologic inhibition of this pathway could provide a promising therapeutic strategy. JTC-801, a selective opioid receptor-like 1 (ORL1) antagonist [43], has been reported to weakly interfere with ligand binding to human κ-opioid receptors (IC50 > 10 μM) [44]. Intriguingly, our analyses indicated a close association between JTC-801 activity and OPRK1 expression in NEPC. To assess its potential as an OPRK1 inhibitor, we performed molecular docking, which revealed a highly favorable binding affinity between JTC-801 and OPRK1 (binding energy = −9.3 kcal/mol) (Fig. 8A). This suggested that JTC-801 could directly interact with OPRK1 and potentially suppress its function. We next evaluated the biological effects of JTC-801 in NEPC cell models. t-NEPC and PC3 cells were treated with increasing concentrations of JTC-801 (0–100 nM) for 48 h. Western blot analysis demonstrated that JTC-801 dose-dependently reduced OPRK1 protein levels, with concomitant decreases in its downstream effector SLC9A3R1 as well as autophagy- and EMT-related markers. Importantly, inhibition of OPRK1 by JTC-801 restored REST protein expression, reversing the molecular changes associated with NE trans-differentiation (Fig. 8B and Supplementary Fig. 7A). Together, these findings provide pharmacologic validation that JTC-801 suppresses OPRK1 signaling, thereby impairing the autophagy-EMT-NED axis and restoring REST stability. This positions JTC-801 as a potential small-molecule therapeutic candidate for the treatment of NEPC.

The above results prompted us to further evaluate the effect of pharmacologic OPRK1 inhibition on NEPC cell growth. Treatment of t-NEPC and PC3 cells with JTC-801 significantly reduced proliferation in a dose-dependent manner, with IC50 values of 16 nM and 25 nM, respectively (Fig. 8C and Supplementary Fig. 7B). Notably, enforced OPRK1 over-expression largely abrogated this inhibitory effect in LNCaP and VCaP cells, increasing IC50 values to 159 nM and 125 nM, respectively (Fig. 8D and Supplementary Fig. 7C). In parallel, JTC-801 suppressed OPRK1 and SLC9A3R1 protein levels while restoring REST expression, whereas OPRK1 over-expression reversed these effects (Fig. 8E and Supplementary Fig. 7D). Consistent with EMT suppression, combined JTC-801 and CQ treatment also impaired migration and invasion of PCa cells (Supplementary Fig. 7E, F).

To assess the therapeutic potential in vivo, a NEPC-like orthotopic xenograft model was established with PC3 cells in castrated nude mice, followed by administration of CQ, JTC-801, or their combination (Fig. 8F). Combination therapy produced the most pronounced inhibition of tumor growth, as reflected by reductions in tumor volume and weight (Fig. 8G-I). Further Western blotting and IHC of tumor tissues further confirmed suppression of OPRK1, SLC9A3R1, NE, autophagy, and EMT markers, together with restoration of REST protein levels (Fig. 8J-K). The above data demonstrate that pharmacologic blockade of OPRK1 with JTC-801, particularly in combination with autophagy inhibition, effectively suppresses NEPC progression by targeting the OPRK1/SLC9A3R1/REST signaling axis.

**Fig. 9:**
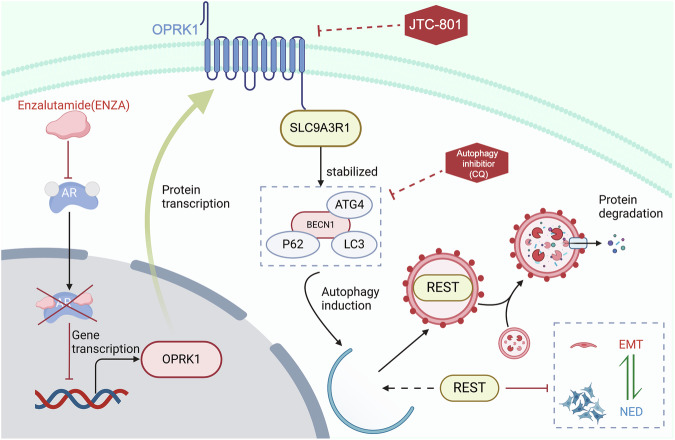
Schema illustrating OPRK1 as an AR-suppressed driver in the neuroendocrine differentiation of PCa. Transcription of OPRK1 is suppressed by AR. Androgen deprivation therapy releases the restrain of OPRK1 by AR. Upregulated OPRK1 promotes lineage plasticity and treatment-induced neuroendocrine differentiation in prostate cancer by upregulating and binding to SLC9A3R1 to activate the autophagy pathway and degrade REST. And JTC-801 combined with CQ represses OPRK1 to suppress cellular autophagy, NED, and EMT in NEPC.

These findings establish REST as a pivotal effector of OPRK1/SLC9A3R1 signaling, mediating NE trans-differentiation in PCa. By directly engaging SLC9A3R1, OPRK1 activates autophagy, which destabilizes REST at the protein level and thereby relieves its transcriptional repression of neuroendocrine programs. This mechanism positions OPRK1 as a central driver of AR-suppressed plasticity and lineage switching toward NEPC. Importantly, pharmacologic inhibition of this axis using the OPRK1 antagonist JTC-801, particularly in combination with the autophagy blocker chloroquine, effectively suppresses NEPC progression by restoring REST function and disrupting the OPRK1/SLC9A3R1/autophagy cascade (Fig. 9A).

## Discussion

The advent of next-generation ARPIs such as ENZA has extended the survival of patients with CRPC by nearly one year [45, 46]. However, persistent AR signaling through splice variants, gain-of-function mutations, or gene amplification often drives therapeutic resistance [34, 47, 48]. More concerning, long-term ARPI treatment is now recognized as a major contributor to t-NEPC, a highly aggressive disease subtype with a median survival of less than 12 months [49]. Despite increasing recognition, the molecular mechanisms underlying ARPI-driven lineage plasticity remain poorly defined, hindering the development of effective therapeutic strategies.

Our study identifies OPRK1, a G protein-coupled receptor, as a critical mediator of lineage switching from AR⁺ prostate adenocarcinoma to NEPC. Integrative bioinformatics analysis of published sequencing datasets and validation in patient-derived CRPC samples revealed robust OPRK1 upregulation in t-NEPC. Functional assays demonstrated that enforced OPRK1 expression repressed AR signaling and induced a NE program characterized by elevated NE markers and EMT-associated genes, whereas OPRK1 silencing suppressed NE differentiation and EMT, particularly under ARPI treatment. Importantly, we uncovered a reciprocal antagonism between AR and OPRK1: AR binds the OPRK1 promoter to inhibit its transcription, while OPRK1 suppresses AR activity and downstream targets, establishing a feedback loop that facilitates ARPI resistance.

We further delineated the downstream signaling cascade of OPRK1. Proteomic profiling identified SLC9A3R1, a PDZ-domain scaffold protein that regulates autophagosome formation [50–53], as the top OPRK1 interactor. Biochemical and structural analyses demonstrated direct OPRK1-SLC9A3R1 binding via the PDZ I domain, which was indispensable for mediating OPRK1-driven autophagy and phenotypic reprogramming. Functionally, this axis destabilized REST, a transcriptional repressor that suppresses neuronal differentiation programs and whose downregulation is a hallmark of NEPC [38, 40–42, 54–58]. Notably, REST repression occurred at the protein stability level rather than transcriptional regulation, as shown by protein stability assays and rescue with autophagy inhibition. Thus, the OPRK1-SLC9A3R1-autophagy axis represents a mechanistic driver of REST loss and NE trans-differentiation.

Given its central role, OPRK1 represents a tractable therapeutic target in NEPC. We evaluated JTC-801, a small-molecule OPRK1 antagonist originally developed for neurological disorders [59, 60]. Molecular docking predicted high-affinity binding between JTC-801 and OPRK1, and in vitro studies confirmed that JTC-801 suppressed OPRK1 and SLC9A3R1 expression while restoring REST protein levels. Functionally, JTC-801 markedly inhibited proliferation and sphere formation of NEPC-like cells, effects that were further potentiated by the autophagy inhibitor CQ. In vivo, combined JTC-801 and CQ treatment significantly reduced tumor growth and restored REST expression in PCa xenografts, validating the therapeutic potential of this combinatorial strategy.

Taken together, our findings establish OPRK1 as a central molecular driver of AR-inhibited plasticity and therapy-induced NEPC. By binding SLC9A3R1 to activate autophagy and destabilize REST, OPRK1 reprograms prostate adenocarcinoma cells toward a NE lineage, thereby fueling ARPI resistance. Pharmacologic inhibition of this pathway with JTC-801, particularly in combination with autophagy blockade, offers a rational therapeutic approach for NEPC. These results not only clarify the mechanistic underpinnings of NEPC emergence but also nominate OPRK1 as a promising therapeutic target to improve outcomes in this lethal subset of advanced PCa.

## Materials and methods

### Cell lines and cell culture

Human PCa cell lines (LNCaP, VCaP, DU145, PC3, 22Rv1, C4-2, and the LNCaP-derived resistant subline t-NEPC), as well as the NEPC line NCI-H660 and HEK293T cells, were obtained from the American Type Culture Collection (ATCC). LNCaP, DU145, PC3, 22Rv1, C4-2, and t-NEPC cells were maintained in RPMI-1640 medium (Gibco) supplemented with 10% fetal bovine serum (FBS; LONSERA) and 80 U/ml penicillin-streptomycin (Gibco). VCaP, NCI-H660, and HEK293T cells were cultured in DMEM (Gibco) containing 10% FBS (LONSERA) and 80 U/ml penicillin-streptomycin. All cell lines were grown in a humidified atmosphere at 37 °C with 5% CO₂. The identity of each cell line was authenticated by short tandem repeat (STR) profiling, and cells were routinely tested to exclude mycoplasma contamination.

### Androgen modulation and establishment of ENZA-resistant t-NEPC cells

For androgen deprivation or stimulation assays, LNCaP or VCaP cells were incubated with 2 nM R1881 (MCE) in the presence or absence of 40 μM enzalutamide (ENZA; MCE) for the indicated durations prior to subsequent experiments. Vehicle-treated cells were used as controls. To generate ENZA-resistant sublines with NED features, LNCaP cells were first xenografted into castrated mice and maintained under continuous ENZA (20 μM) treatment for six months. The resulting tumors were dissociated into single cells, which were subsequently cultured in vitro in ENZA-containing medium (20 μM) for an additional six months. The established resistant cells, exhibiting NED phenotypes, were designated as t-NEPC cells.

### Patients

All procedures involving patient samples and clinical information were approved by the Medical Ethics Committee of the First Affiliated Hospital of Nanjing Medical University. Fresh tumor specimens were collected, pathologically classified, and processed for paraffin embedding. Sections of 5 μm thickness were prepared for hematoxylin and eosin (HE) staining to evaluate histomorphology. When characteristic features of NEPC were observed, additional immunohistochemical (IHC) staining was performed to assess the expression of NED markers. Diagnosis of NEPC was rendered by a board-certified pathologist from the Department of Pathology, based on both histological morphology and elevated NED marker expression. For IHC analysis, paraffin sections from NEPC samples were stained for AR, OPRK1, and synaptophysin (SYP), whereas all other PCa samples were examined for OPRK1 expression. The following antibodies were used: OPRK1 (ab113533, Abcam), AR (5153, Cell Signaling Technology), neuron-specific enolase (NSE, 10149-1-AP, Pråoteintech), and SYP (17785-1-AP, Proteintech).

### Bioinformatics analysis

Transcriptomic datasets relevant to prostate cancer were obtained from two major repositories: the cBioPortal for Cancer Genomics and the NCBI Gene Expression Omnibus. The datasets covered a spectrum of disease states, including primary adenocarcinoma, CRPC, NEPC, and, where available, metastatic tumors (e.g., Beltran 2016, GSE66187, GSE107299, GSE202885). Expression profiles were processed following the normalization pipelines reported in the original publications to ensure data consistency.

Comparative analyses were performed to assess OPRK1 expression across subtypes. Correlation tests were applied to explore the association of OPRK1 with AR activity and NED marker expression. Pathway-level alterations linked to OPRK1 were interrogated using gene set enrichment analysis (GSEA), and survival outcomes were evaluated in TCGA-PRAD, DKFZ2018, GSE107299, GSE21034 and GSE116918 cohorts.

All statistical analyses were conducted in R (v4.2.0) with Bioconductor packages, adopting a significance threshold of *p* < 0.05 unless otherwise specified. Cross-validation across multiple cohorts confirmed consistent upregulation of OPRK1 in NEPC and highlighted its strong association with lineage plasticity, neuroendocrine differentiation, and unfavorable prognosis.

### RNA-seq and RT-PCR

We used TRIZOL to extract total RNA from cells according to the manufacturer’s instructions (Invitrogen, CA, USA). Then we used oligo-dT primers with adapter attached to reverse transcribe the mRNA to barcoded cDNA fragments. Barcoded cDNA libraries were sequenced using an Illumina Novaseq 6000 platform. After quality assessment, RNA-seq reads were compared with the reference genome (GRCh37/hg19) using HISAT 2. Subsequently, StringTie was used for assembling and quantifying the abundance of transcripts. Standardized data was analyzed for differential gene expression using DESeq2.

All RT-PCR in this experiment were performed using HiScript III RT SuperMix for qPCR (R333-01, Vazyme) according to the manufacturer’s instructions for reverse transcription. Then we used ChamQ Universal SYBR qPCR Master Mix (Q341-01, Vazyme) in triplicate for real-time PCR. The primers used in this study are listed in Supplementary Table 3.

### Immunoblotting

We prepared whole cell lysates in cell lysis buffer (P0013, Beyotime). Approximately 107 cells were cleaved using a 500 μl lysis buffer containing a mixture of protease inhibitors (P1006, Beyotime). Then we conducted SDS-PAGE and immunoblotting experiments using conventional methods. The antibodies used for immunoblotting include GAPDH (60004-1-Ig, Proteintech), OPRK1 (ab113533, abcam), AR (5153, Cell Signaling Technologies), PSA/KLK3 (5365, Cell Signaling Technologies), NKX3.1 (83700, Cell Signaling Technologies), NSE (10149-1-AP, Proteintech), SYP (17785-1-AP, Proteintech), HCG/CgA (25014-1-AP, Proteintech), CK8 (17514-1-AP, Proteintech), SLC9A3R1 (29771-1-AP), Beclin1 (11306-1-AP, Proteintech), REST (22242-1-AP, Proteintech), N-Cadherin (R380671, zenbio), E-Cadherin (R22490, zenbio), VIM/vimentin (R22775, zenbio), LC3B (14600-1-AP, Proteintech) and P62 (18420-1-AP, Proteintech). Using BIO-RAD GelDoc XR to detect the blot bands.

### Chromatin immunoprecipitation (ChIP) assay

48 h prior to the ChIP experiment, we treated LNCaP cells separately with 2 nM R1881, while also treating LNCaP cells with 2 nM R1881 and 20 μM ENZA or DMSO vehicle. Our ChIP was performed using the high-sensitivity ChIP Enzymatic Chromatin IP Kit (ab185913, abcam) following the manufacturer’s instructions. We fixed the cells (5 × 10^7^ per cell line) with 1% formaldehyde for 12 min. Then, the reaction was terminated by adding 1 M glycine to achieve a final concentration of 0.125 M. Finally, the sample chromatin was fragmented by nuclease digestion and ultrasound treatment. Use the following antibodies for CHIP: Rabbit anti-AR antibodies (5153, Cell Signaling Technologies), normal rabbit IgG (2729, Cell Signaling Technologies), rabbit anti-H3K4me3 (84908-2-RR, Proteintech), rabbit anti-H3K27ac (82902-1-RR, Proteintech), rabbit anti-H3K27me3 (9377, Cell Signaling Technologies), rabbit anti-H3K9me2/3 (5327, Cell Signaling Technologies) and rabbit anti-histone H3 antibodies (17168-1-AP, Proteintech). The final step was to use primers designed based on predicted androgen response elements to quantify immunoprecipitated DNA through RT-PCR.

### Plasmids

OPRK1 over-expression vector (LV-OPRK1-3X Flag-GFP-puromycin) and REST over-expression vector (pLenti-U6-suCMV-Rsv [RFP-Puro]) were constructed by Genechem (Shanghai, China). AR over-expression vector, pLKO.1-shAR vector, shOPRK1-1plasmids, shOPRK1-2 plasmids and shSLC9A3R1 plasmids were constructed by KeyFEN (Jiangsu, China). pLKO.1-shAR vector sequence is “AAGACGCUUCUACCAGCUCACTT” and “AUCCUGGAGUUGACAUUGGUGTT”. shOPRK1 sequence is “AUCUUGCACAGCACAUCCTT” and “GGAUGUGCUGUGCAAGAUATT”. shEHMT2 sequence is “cgAGAGAGTTCATGGCTCTTT” and “TGCGTGCTGTTATTCCTGTCA”. EED shRNAs (shEED-1: TRCN0000021205, shEED-2: TRCN0000021206), EZH2 shRNAs (shEZH2-1: TRCN0000018365, sh-EZH2-2: TRCN0000010475) and SLC9A3R1 shRNAs (shSLC9A3R1-1: TRCN0000068583, shSLC9A3R1-2: TRCN0000068584) were from MISSON shRNA Library (Sigma-Aldrich). SUZ12 shRNAs were from Santa Cruz (sc-45597-SH). The shSLC9A3R1 sequence (5’—3’) is subcloned into the pRS shRNA cloning plasmid (Origene, TR20003). By inserting the kpn I/XhoI fragment containing the OPRK1 promoter (-1987bp ~ +1991bp) cloned from LNCaP cells along with luciferase CDS in pGL4.17 vector, a pGL4.17-OPRK1-FL-Lluciferase plasmid was constructed. Next, we used the Vazyme ClonExpressTM II One Step Cloning Kit to generate the OPRK1 promoter plasmid with three ARE mutations. Overlapping PCR introduced site-specific point mutations.

The ARE primers:

ARE1-F: CAGTCTGGGGACAGTTCCAC

ARE1-R: GCAGACTTCAGGGGCTCATT

ARE2-F: CAGGTGATGCCAAGAGCTGA

ARE2-R: AAACTGCAGACTTCAGGGGC

ARE3-F: AAGCTTTGGGCTTTCGAGGT

ARE3-R: AACTGCAGACTTCAGGGGC

### Lentiviral transfection

We infected LNCaP and VCaP with lentiviral particles and 8ug/ml of Sigma Aldrich at 60% confluence to produce stable transgenic lines. Then, positive transfected cells were selected and enriched by applying puromycin (5 μg/ml) to the culture medium for 2 weeks.

### Luciferase assay

We used OPRK1 promoter luciferase lentivirus to transfect LNCaP cells. Then we inoculated stable transfected LNCaP cells into a 12 well plate and used vehicle (DMSO), 2 nM R1881 or 40 μM ENZA processing. Finally, according to the manufacturer’s protocol, we used the Dual Luciferase Reporter Assay System (Promega, E2920) to detect the firefly luciferase activity after 24 h of treatment. The final step is to standardize the luciferase activity of fireflies relative to the number of cells.

### Experiments and analysis of cell proliferation (CCK8 and EdU), colony formation, migration/invasion and tumorsphere formation

To determine cell proliferation, we inoculated the cells onto 96-well plates (1000 cells/well) and grew them in medium with or without ENZA for up to 7 days. Finally, we measured cell viability using CCK8 (Beyotime, C0038) according to the manufacturer’s instructions.

For EdU assay, we seeded cells in triplicate into six-well plates (6 × 10^4^ cells/well), cultured for 24 h, and then Incubated with 200 μM EdU for 1.5 h. Finally, the samples were stained and visualized.

For the colony formation experiment, we evaluated colony formation by dividing cells into triplicate and inoculating them in six-well plates (1000 cells/well) for 10 days. Afterwards, we fixed the cell colonies, stained them, and counted them.

For the cell migration/invasion assay, 2 × 10^4^ cells were plated in upper chamber with serum-free medium, separated from 20% FBS-containing medium containing or not matrix adhesive in lower chamber. After 24 h incubation at 37 °C in 5% CO_2_, non-migrated cells were removed. The number of migrated cells was stained with crystal violet (Beyotime, C0121) and then counted using microscope in four randomly chosen fields.

For tumorsphere formation, we inoculated a single-cell suspension of 10^4^ cells/ml into 6-well low-adhesion plates with DMEM-F12 containing 20 μM ENZA. Then we count the Spheroid (>100 μm) under a light microscope.

### Immunofluorescence (IF)

We conducted IF assays to determine the expression of relevant genes and cellular co localization. Firstly, we incubate the cells with primary antibodies at 4 °C overnight, followed by incubation with corresponding secondary antibodies and DAPI. Finally, we used Stellaris STED laser confocal microscopy (LEICA, German) to capture images.

### Tumor xenograft experiment

All animal experiments were conducted under protocols approved by the Institutional Animal Care and Use Committee of Nanjing Medical University (IACUC-2308027) and complied with ARRIVE guidelines. Mice were housed in specific pathogen-free (SPF) facilities under controlled temperature (22 ± 2°C), humidity (50–60%), and a 12-h light/dark cycle, with ad libitum access to food and water. For tumor xenograft assays, male BALB/c nude or NSG mice (6–8 weeks old, 18–22 g) were used. A total of 6 × 10^6^ cells suspended in 100 μl phosphate-buffered saline (PBS) containing 50% Matrigel were subcutaneously injected into the right flank. To assess castration resistance in vivo, NSG mice were surgically castrated two weeks prior to tumor cell implantation. Animals were randomly allocated into treatment or control groups using a computer-generated randomization sequence. Investigators performing tumor measurements and endpoint analyses were blinded to group allocation. No statistical methods were used to predetermine sample size; group sizes (*n* = 5–8 per group) were chosen based on prior studies and pilot experiments to ensure reproducible tumor growth differences. Exclusion criteria (unexpected death unrelated to treatment, injection failure, or technical errors in sample processing) were defined a priori, but no animals met these criteria. At the study endpoint, animals were humanely euthanized by CO_2_inhalation followed by cervical dislocation. Tumors were excised, photographed, weighed, and processed for subsequent molecular and histological analyses.

### Immunoprecipitation

For immunoprecipitation, NP-40 buffer containing protease inhibitor cocktail and phosphatase inhibitors (Beyotime, P1045) was used for cell lysis. Protein lysates were incubated with protein A/G magnetic beads (Beyotime, P2108) conjugated with primary antibody or FLAG beads (Beyotime, P2181M) at 4 °C for 3 h. Then the beads were washed with NP-40 buffer and analyzed by immunoblotting protocol.

### Protein stability assay

HEK293T or LNCaP cells were transfected with SLC9A3R1 knockdown plasmids, REST over-expression plasmids, and OPRK1-Flag plasmids or empty vector, as described earlier. For dose-dependent protein stability determination, LNCaP cells were transfected with 5 μg, 10 μg, I5 μg OPRK1-Flag or empty vector. 48 h after transfection, all specimens were lysed with cell lysate to detect SLC9A3R1 and REST protein levels. For time-dependent protein stability determination, we use 2 μg OPRK1-Flag vector and 2 μg REST expression vector to co-transfected 10^6^ 293 T cells, while 10^6^ LNCaP cells were co-transfected with 10μ OPRK1-Flag or empty vector. Then, the cell samples were lysed with cell lysate at 24, 48 and 72 h after transfection. For samples treated with MG132, after 48 h of transfection, we used 10 μg OPRK1-Flag or empty vector to transfect LNCaP, which were cultured in medium containing 10 μg/ml cycloheximide (HY-12320, MCE). After washing with NP-40 buffer, we boiled the beads together with SDS loading buffer for 10 min for immunoblotting analysis.

### Label-free quantitative proteomics analysis

For label-free quantitative proteomics, LNCaP cells stably overexpressing FLAG-tagged OPRK1 and corresponding FLAG-vector controls were lysed under denaturing conditions, and equal protein amounts from biological triplicates were immunoprecipitated with anti-FLAG magnetic beads to enrich OPRK1-associated complexes. After extensive washing, bound proteins were eluted, digested with trypsin, and subjected to liquid chromatography–tandem mass spectrometry (LC–MS/MS) analysis in label-free quantification mode. The acquired spectra were processed with MaxQuant and searched against the UniProt human database using the Andromeda engine, and relative protein abundance was calculated from normalized spectral intensities. Differentially enriched proteins between OPRK1-overexpressing and control groups were determined using a fold-change cutoff of >1.5 and adjusted *p* < 0.05, thereby identifying candidate OPRK1-interacting proteins for downstream functional characterization.

### GST pull-down assay

Recombinant human GST-OPRK1 protein or GST protein was mixed with recombinant human His-MBP-SLC9A3R1 protein in NP-40 buffer at 4 °C overnight. Then we incubated the protein mixture with GST beads (Beyotime, P2138) at 4 °C for 4 h. Then we digested and desalinated the immunoprecipitate using sequencing-grade trypsin. And the desalinated peptides were separated on the EASY-nLC 1200 UPLC system (Thermo Fisher Scientific), and finally analyzed by MS/MS using Orbitrap Exploris 480 (Thermo Fisher Scientific). The obtained MS/MS data were finally processed using the Proteome Discoverer search engine (v.2.4) and subjected to label-free quantitative analysis.

### Statistical analysis

All statistical analyses in this study were conducted using GraphPad 7.0 software and the specific statistical methods used are shown in the corresponding legend. *P* < 0.05 is considered to have significant differences.

## Supplementary information

Supplement figures and tables and Original lmages for Blots

## Data Availability

Datasets during this study can be accessed on reasonable request from the corresponding author.
