# Supplementary material for: OPRK1 drives SLC9A3R1 progression to neuroendocrine prostate cancer
Source: Cell Death Dis. 2025 Dec 7;17(1):68. doi: 10.1038/s41419-025-08279-4 (PMC12828004; doi:10.1038/s41419-025-08279-4)
Supplement: Supplementary file 1 — Supplement figures and tables and Original lmages for Blots [file 41419_2025_8279_MOESM1_ESM.pdf]

Supplementary Information

Supplementary Data

Supplementary Figure 1, related to Figure 1

Supplementary Figure 2, related to Figure 2

Supplementary Figure 3, related to Figure 3

Supplementary Figure 4, related to Figures 4

Supplementary Figure 5, related to Figure 5

Supplementary Figure 6, related to Figure 6

Supplementary Figure 7

Supplementary Figure 8, related to Figure 7

Supplementary Table 1

Supplementary Table 2

Supplementary Table 3

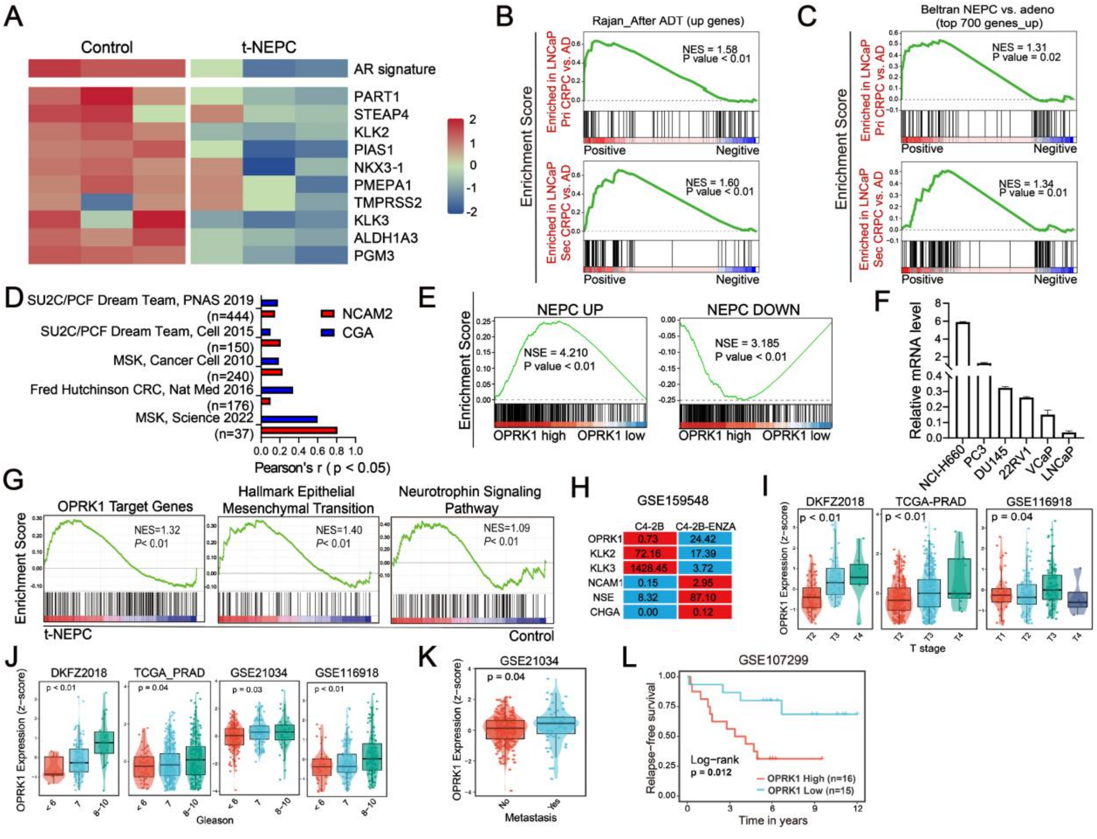

Supplementary Figure 1. OPRK1 is highly expressed and has poor prognosis in t-NEPC . A Heatmap depicting AR signature genes expression in t-NEPC cells (n=3) and LNCaP control cells (n=3). **B-C** The GSEA of Rajan_After ADT (up genes) and Beltran NEPC vs. Adeno (top 700 genes_up). **D** Pearson correlation analysis of OPRK1 versus NCAM2 and CGA mRNA in the indicated datasets from the cBioPortal database. **E** GSEA of NEPC gene signatures for the comparisons of OPRK1-high versus OPRK1-low patient samples in the TCGA-PRAD cohort. **F** qPCR of OPRK1 in a panel of indicated human PCa cell lines (n=3). **G** GSEA of the indicated gene signatures for the comparisons of LNCaP-ENZA vs. control LNCaP cells. **H** OPRK1, KLK2, KLK3, NCAM1, NSE and CHGA mRNA levels (unit: RPKM) in the RNA-seq dataset GSE159548. C4-2B: C4-2B before ENZA treatment; C4-2B-ENZA: C4-2B after ENZA treatment. **I** High expression level of OPRK1 is significantly associated with higher T stage in PCa patients in DKFZ2018, TCGA_PRAD, GSE21034 and GSE116918 database. **J** High expression level of OPRK1 is significantly associated with higher Gleason score in PCa patients in DKFZ2018, TCGA_PRAD and GSE116918 database. **K** High expression level of OPRK1 is significantly associated with easier metastasis in PCa patients in GSE21034 database. **L** High expression level of OPRK1 is significantly associated with worse survival in PCa patients in GSE107299 database.

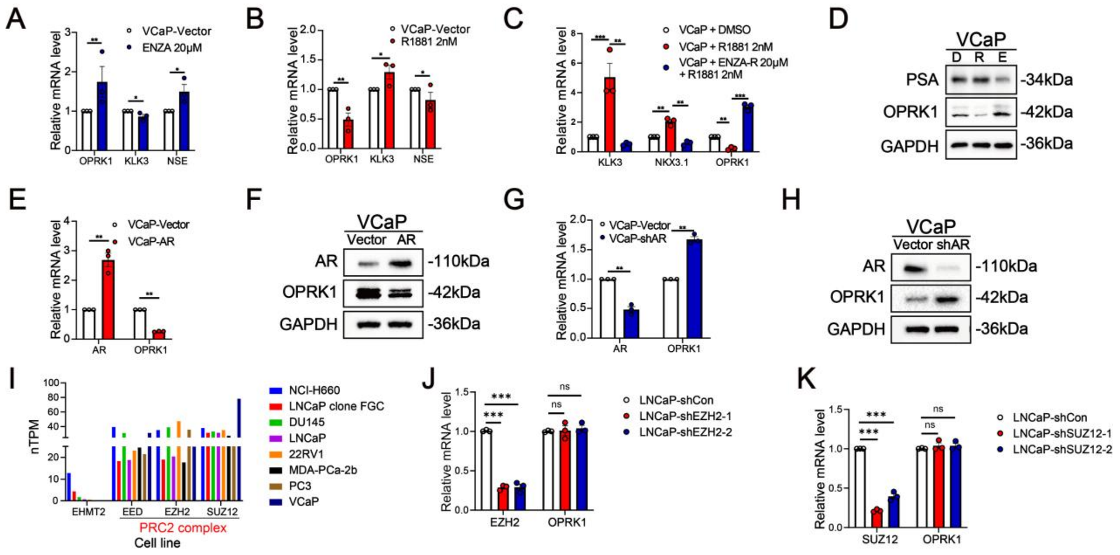

Supplementary Figure 2. AR suppresses the transcription of OPRK1 in PCa cells. A-B qPCR of OPRK1, KLK3 and NSE in VCaP cells treated with ENZA (20μM, 6h) or upon R1881 stimulation (10nM, 6h) (n=3). **C-D** q-PCR (left panel) and immunoblots (right panel) of indicated

genes in VCaP cells treated with R1881(20μM, 7days) with or without ENZA (10nM, 7days) (n=3). **E-F** q-PCR (left panel) and immunoblots (right panel) of AR and OPRK1 in stably AR over-expressing and control VCaP cells (n=3). **G-H** q-PCR (left panel) and immunoblots (right panel) of AR and OPRK1 in AR knockdown and control VCaP cells (n=3). **I** q-PCR analysis of EHMT2 and PRC2 (EED, EZH2 and SUZ12) in different human PCa cell lines. **J** q-PCR analysis of EZH2 and OPRK1 in shRNA mediated silencing of EZH2 in LNCaP (n=3). **K** q-PCR analysis of SUZ12 and OPRK1 in shRNA mediated silencing of SUZ12 in LNCaP (n=3).

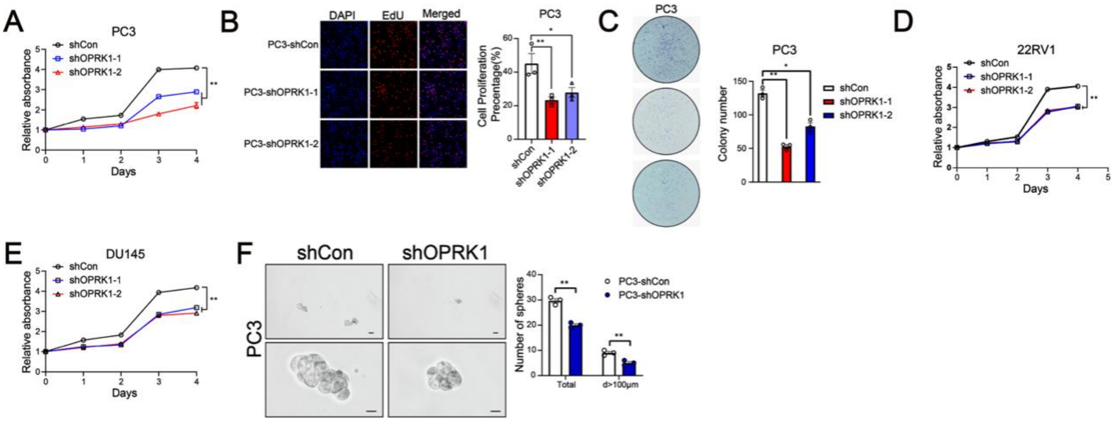

Supplementary Figure 3. OPRK1 knockdown attenuates proliferative and stem-like phenotypes of NEPC cells. **A** CCK-8 assays showing reduced proliferation of PC3 cells following OPRK1 knockdown (n=3). **B** Representative EdU staining and quantification in PC3-shCon and PC3-shOPRK1 cells (n=3). **C** Colony formation assays demonstrating impaired clonogenic growth upon OPRK1 depletion in PC3 cells (n=3). **D-E** Growth curves of OPRK1-silenced 22RV1 and DU145 cells assessed by CCK-8 assays (n=3). **E** Tumorsphere formation assays showing decreased number and size of spheres in PC3 cells after OPRK1 knockdown (n=3). Data represent the mean ± SEM. *$p < 0.05$; **$p < 0.01$.

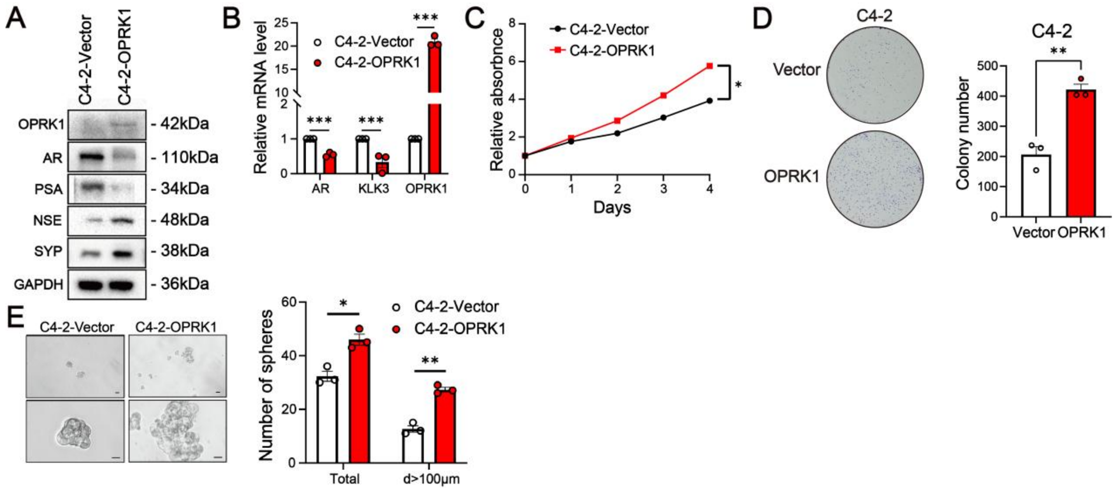

Supplementary Figure 4. OPRK1 over-expression suppresses AR signaling and enhances NE features in C4-2 cells. **A** Immunoblot analysis of AR, PSA, NSE, and SYP in C4-2 cells stably transfected with vector or OPRK1. GAPDH was used as loading control. **B** qPCR quantification of AR, KLK3, and OPRK1 mRNA levels in vector- and OPRK1-overexpressing C4-2 cells (n = 3). **C** CCK-8 proliferation assays showing enhanced growth of C4-2-OPRK1 cells compared with controls (n = 3). **D** Representative images and quantification of colony formation assays in vector- and OPRK1-overexpressing C4-2 cells (n = 3). **E** Representative images and quantification of tumorsphere formation, showing increased sphere numbers and size (>100 μm) upon OPRK1 over-expression in C4-2 cells (n = 3).. Data represent the mean ± SEM. *$p < 0.05$;**$p < 0.01$.

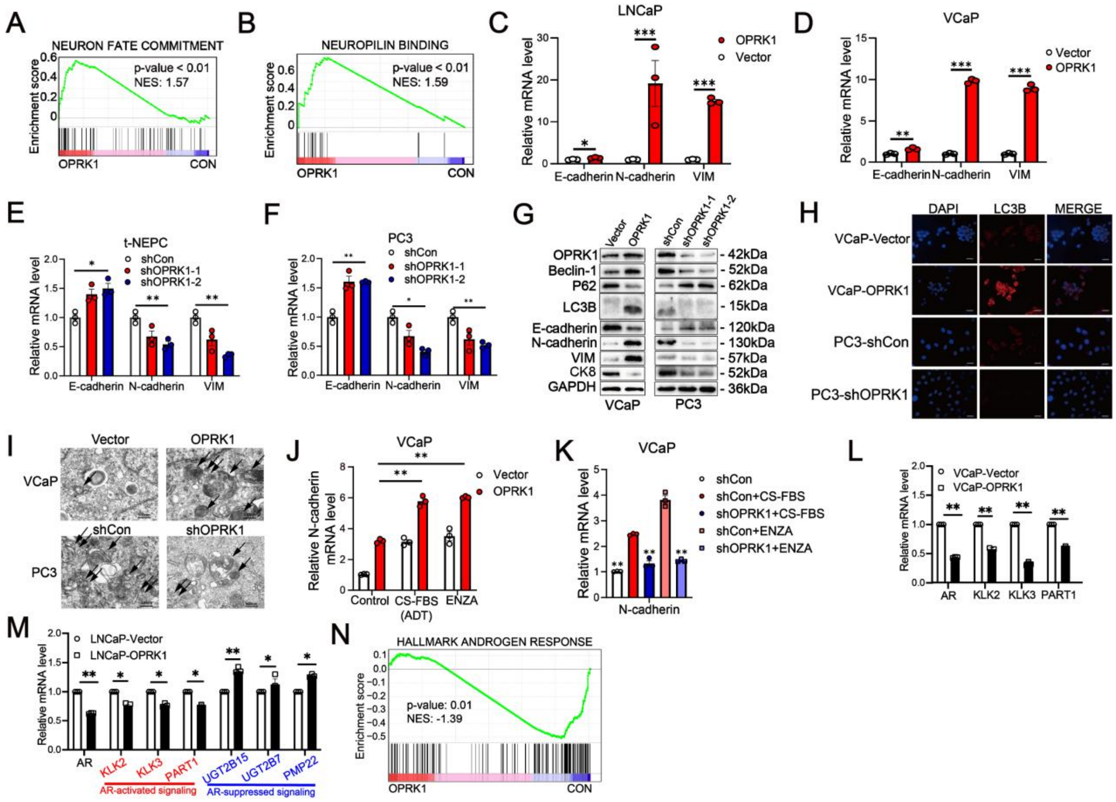

Supplementary Figure 5. OPRK1 drives the NED and down-regulates the AR signaling. A-B GSEA of nervous system related gene signature. **C** qPCR analysis of EMT markers (E-cadherin, N-cadherin and VIM) mRNA levels of OPRK1 over-expressing and control LNCaP/VCaP cells (n=3). **E-F** qPCR analysis of EMT markers mRNA levels of OPRK1 knockdown and control t-NEPC/PC3 cells (n=3). **G** Immunoblots of OPRK1, autophagy markers (Beclin-1, P62 and LC3B) and EMT markers in OPRK1 over-expressing VCaP/control cells and OPRK1 knockdown PC3/control cell. **H** The expression and location of LC3B markers in VCaP-Vector/OPRK1 and PC3-shCon/shOPRK1 cells were analyzed by immunofluorescence staining. **I** Electron microscopy images showing lysosomes and autophagosomes within VCaP-Vector/OPRK1 and PC3-shCon/shOPRK1 cells. **J** qPCR analyses were used to examine N-cadherin expression in VCaP cells with and without over-expression of OPRK1 under ADT (CS-FBS) or ENZA treatment. **K** qPCR analyses were used to examine N-cadherin expression in VCaP cells with and without knockout of OPRK1 under CS-FBS or ENZA treatment. **L** q-PCR analysis of AR, indicated AR-activated genes in stable OPRK1 over-expressing VCaP and control cells. **M** q-PCR analysis of AR, indicated AR-activated genes and AR-suppressed genes in stable OPRK1 over-expressing LNCaP and control cells. **N** GSEA analysis of "HALLMARK ANDROGEN

RESPONSE” of RNA-seq data from stable OPRK1 over-expressing LNCaP and control cells.

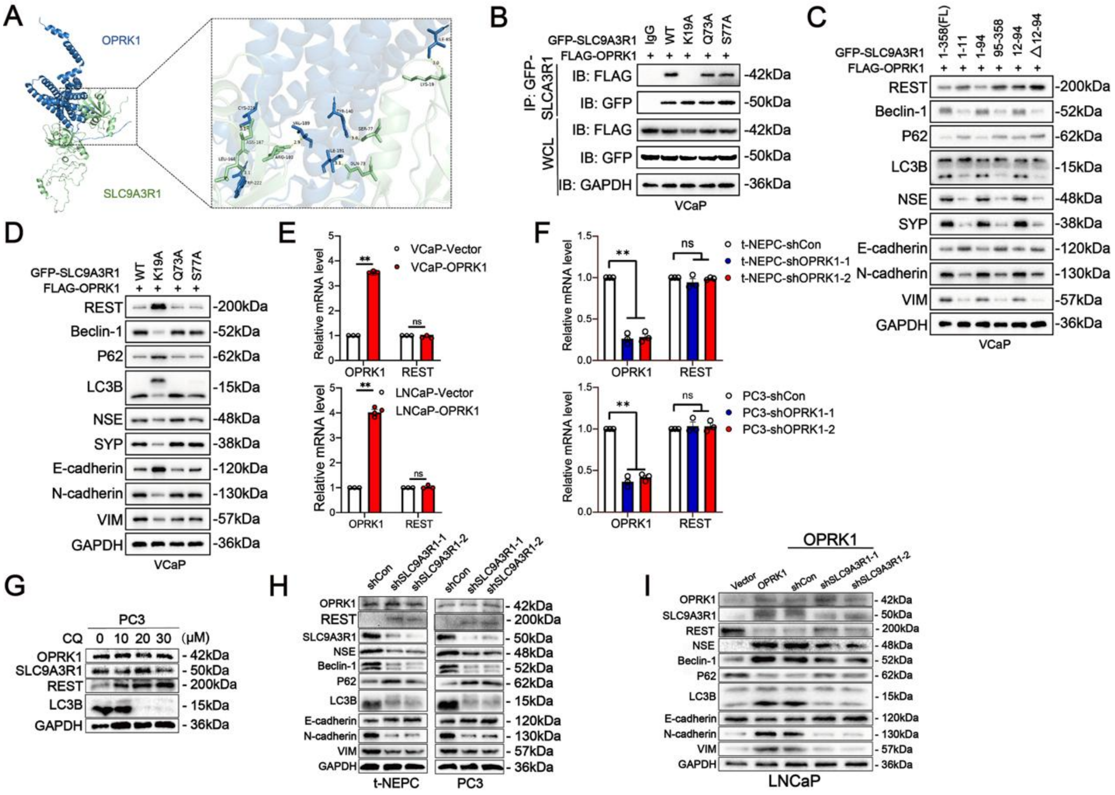

Supplementary Figure 6. SLC9A3R1 is a critical OPRK1-interacting partner mediating REST degradation via autophagy in PCa cells. A Molecular docking model showing predicted structural interaction between OPRK1 and SLC9A3R1, with detailed binding interfaces. **B** Co-IP analysis in VCaP cells co-expressing GFP-SLC9A3R1 and FLAG-OPRK1, confirming physical interaction. **C** Functional validation of SLC9A3R1 domains: immunoblotts analysis of REST, autophagy markers (Beclin-1, P62, LC3B), NE markers (NSE, SYP), and EMT markers (E-cadherin, N-cadherin, Vimentin) in LNCaP/VCaP cells co-transfected with FLAG-OPRK1 and full-length (FL) or truncation mutants of GFP-SLC9A3R1. **D** Point mutation analysis of the PDZ I domain (K19A, Q73A, S77A) demonstrates that residue K19 is indispensable for OPRK1-SLC9A3R1 binding, as shown by co-IP assays. **F** qPCR of OPRK1 and REST expression in OPRK1-knockdown versus control t-NEPC and PC3 cells (n=3). **G** Immunoblotts of OPRK1, SLC9A3R1, REST, and LC3B in PC3 cells treated with increasing concentrations of CQ (0-30 μM, 48h). **H** Immunoblot analysis of REST, SLC9A3R1, and autophagy/EMT markers in t-NEPC and PC3 cells with SLC9A3R1 knockdown. **I** Immunoblot validation of REST degradation in

LNCaP cells co-expressing OPRK1 and SLC9A3R1 mutants, confirming dependence on PDZ I－mediated interaction. Data represent the mean ± SEM. *$p < 0.05$; **$p < 0.01$; ***$p < 0.001$.

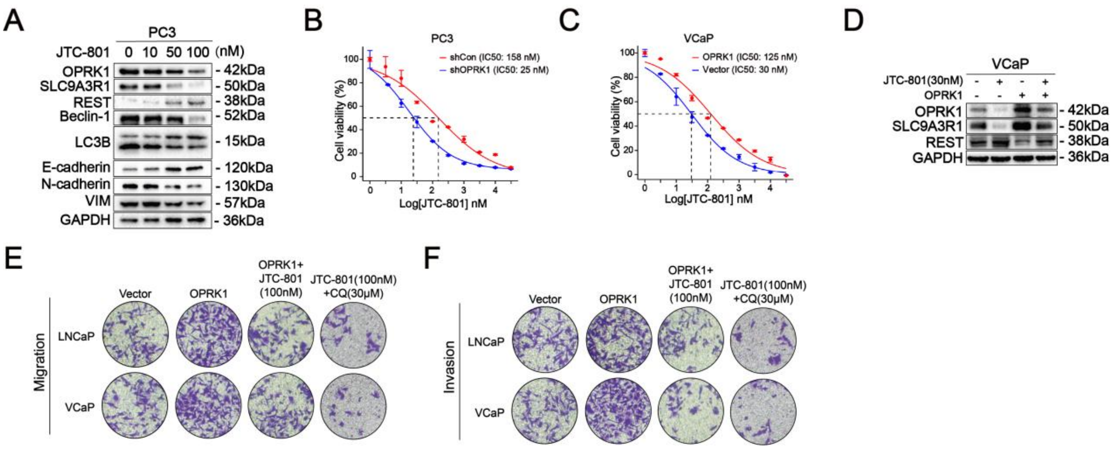

Supplementary Figure 7. JTC-801 combined with CQ inhibits OPRK1/autophagy axis. **A** PC3 Cells treated with JTC-801 (0nM, 10nM, 50nM, 100nM) and collected 48h later for detection of OPRK1 by immunoblotting analysis. **B** PC3 Cells were treated with JTC-801 alone or along with OPRK1 knockdown plasmids for 48h. The IC50 of JTC-801 was detected by CCK-8 assay. **C** VCaP Cells were treated with JTC-801 alone or along with OPRK1 over-expressing plasmids for 48h. The IC50 of JTC-801 was detected by CCK-8 assay. **D** VCaP transfected with JTC-801 alone or along with OPRK1 over-expression pla smid were collected 48h later for detection of OPRK1, SLC9A3R1 and REST by immuno blotting assay. **E-F** Representative images of transwell migration/invasion assay in control LNCaP/VCaP and over-expressing OPRK1 LNCaP/VCaP with or without JTC-801 or JTC-801 combined with CQ (n=3).

Supplementary Table 1. List of OPRK1 target genes defined as the top 100 differentially expressed genes by fold change in absolute value (p<0.001) in OPRK1-knockdown relative to control t-NEPC cells by RNA-seq.

**Gene Symbol**	**Log2 Fold Change**	***P* Value**
PLA2G4B	-1141.131	2.44E-43
ABHD14A	-948.797	1.05E-23
SULT1A3	-850.792	2.07E-25
RGPD5	-537.362	1.18E-23
HIST1H4J	-366.713	1.54E-13
SULT1A4	-343.096	2.22E-75
TBC1D3H	-325.155	2.78E-12
GTF2IP5	-241.908	2.55E-11
P2RY12	-210.358	3.16E-10
TXNDC4	-200.727	2.69E-09
CDH17	-11.328	2.70E-09
GALNT4	-7.727	4.47E-13
RAD51L3	-6.721	5.52E-09
SLC22A3	-5.164	6.13E-20
TEN1	-4.402	6.75E-12
WNT5A	-4.401	2.01E-21
NRP2	-4.114	1.84E-18
SOX4	-3.535	2.55E-14
CCL20	-3.381	3.56E-13
TNC	-3.073	7.24E-14
FAM198B	-2.891	8.06E-11
TNFSF15	-2.841	6.54E-09
JAG2	-2.838	3.08E-11
LEF1	-2.776	3.21E-11
VAC14	-2.754	5.48E-11
GPR161	-2.433	9.82E-09
LMLN	-2.432	9.62E-10
TUBA1A	-2.377	5.70E-09
LGALS3BP	2.486	9.65E-09
PGK1	2.528	1.26E-10
HPCAL4	2.552	5.29E-09
PPAP2A	2.569	1.11E-10
PDK1	2.609	1.13E-10
SRRM3	2.644	1.69E-09
GPRC5B	2.668	2.95E-10
FMNL1	2.673	1.99E-11
P4HA1	2.696	7.93E-09
THBS2	2.713	6.03E-11
CARNS1	2.863	6.80E-11

CAMK2A	3.013	9.71E-09
FN2	3.076	7.75E-12
PLA2G2A	3.097	2.05E-12
VEGFB	3.109	5.32E-14
SEMA6B	3.206	5.05E-10
EGFR	3.242	2.93E-09
STC1	3.254	1.07E-13
HMOX1	3.317	1.18E-14
FSD1	3.317	5.42E-10
SYT12	3.473	6.16E-09
NEURL1	3.495	1.84E-10
LOC101448202	3.578	3.37E-10
PFKFB4	3.637	3.47E-17
RND3	3.661	1.80E-09
RAB31	3.721	3.75E-15
DPYSL4	3.788	1.47E-13
TEX19	3.822	7.79E-19
LRG1	3.979	6.06E-10
BNIP3	4.152	5.37E-22
LGALS1	4.281	2.26E-09
RGPD5	4.405	6.54E-14
S1PR3	4.445	3.60E-13
TRPM8	4.668	4.61E-14
FGFR3	4.816	2.91E-26
NDRG1	5.005	3.23E-09
SLC16A3	5.033	1.13E-25
C3	5.485	1.55E-09
KLK3	5.574	2.15E-14
LOC642423	5.619	6.25E-27
ARHGAP23	5.799	2.33E-11
PGF	5.837	3.70E-17
NPTX1	5.981	4.51E-09
SOX8	6.173	6.83E-09
SUSD2	6.313	1.08E-14
SLPI	6.585	4.29E-34
GJB6	6.762	2.81E-11
LGI3	6.788	1.27E-16
PLA2G4D	6.826	4.59E-28
KLK2	7.179	3.47E-30
FAM43A	7.511	5.74E-18
CA6	8.332	2.03E-09
TGFB1	8.785	3.11E-20
C2orf54	9.189	1.17E-35

PCP4L1	9.247	3.54E-09
CCDC16	10.417	3.76E-10
OLFML2B	11.437	5.15E-12
MUC5A	11.625	4.39E-12
C10orf54	12.943	5.20E-34
HBA1	14.153	3.66E-10
QPRT	14.679	5.02E-09
PLOD1	18.811	5.03E-46
MYOD1	20.944	5.38E-13
HTR5A	31.071	4.75E-11
CTAGE4	50.158	7.25E-18
ZNF20	133.133	6.45E-09
KISS1R	141.138	2.38E-09
HIST1H4K	197.170	1.78E-11
RPP21	305.290	8.07E-17
GNG10	493.341	1.16E-19
NUDT4P2	537.539	4.05E-27
SERF1B	711.353	1.04E-31

Supplementary Table 2. List of additional NE genes downregulated in OPRK1-knockdown relative to control t-NEPC cells by RNA-seq.

**Gene Symbol**	**Log2 Fold Change**
ASCL1	-19.113
PCSK2	-18.623
INSM1	-12.123
MYT1	-1.623
IGFBPL1	-1.741
GCG	-1.921
CREB1	-1.811
ATN1	-1.242
SYP	-1.194
FOXM1	-1.176
CD56	-1.206
STMIN1	-1.211

Supplementary Table 3. primers for q-PCR in this study.

GAPDH(F)	CTGGGCTACACTGAGCACC
GAPDH(R)	AAGTGGTCGTTGAGGGCAATG
OPRK1(F)	TAGTGTTCGTCGTGGGCTTG
OPRK1(R)	TCTTCATCTTTGTGTATCGGATGA
EBP50/SLC9A3R1(F)	GGCTGGCAACGAAAATGAGC
EBP50/SLC9A3R1(R)	TGTCGCTGTGCAGGTTGAAG
ARfull(F)	CTGGGAAGGGTCTACCCAC
ARfull(R)	GGTGCTATGTTAGCGGCCTC
PSA/KLK3(F)	CACAGCCTGTTTCATCCTGA
PSA/KLK3(R)	AGGTCCATGACCTTCACAGC
KLK2(F)	TCAGAGCCTGCCAAGATCAC
KLK2(R)	CACAAGTGTCTTTACCACCTGT
NKX3.1(F)	CCCACACTCAGGTGATCGAG
NKX3.1(R)	GAGCTGCTTTCGCTTAGTCTT
TMPRSS2(F)	GTCCCCACTGTCTACGAGGT
TMPRSS2(R)	CAGACGACGGGGTTGGAAG
PART1(F)	AAGGCCGTGTCAGAACTCAA
PART1(R)	GTTTTCCATCTCAGCCTGGA
UGT2B15(F)	CCAACCAATGAAGCCCCTG
UGT2B15 (R)	GTTGTGAGCTGCGACTCGAA
UGT2B7(F)	GATCCCAACAACTCATCCGCT
UGT2B7 (R)	CAGCAGCTCACTACAGGGAA
PMP22(F)	ATCGTCAGCCAATGGATCGTG
PMP22(R)	AGAAACAGTGGTGGACATTTCC
NSE/ENO2(F)	GCCAAAGGTCTTTTCCGG
NSE/ENO2(R)	CCTTCAGGACACCTTTGC
CGA(F)	TCCAAGGCGCCAAGGA
CGA(R)	CATCTTCAAAACCGCTGTGTTTC
SYP(F)	TCAGTTCCGGGTGGTCAAG
SYP(R)	AAGACCCATTGCAGCACCTT
E-cadherin(F)	CGAGAGCTACACGTTCACGG
E-cadherin(R)	GGGTGTCGAGGGAAAAATAGG
N-cadherin(F)	TCAGGCGTCTGTAGAGGCTT
N-cadherin(R)	ATGCACATCCTTCGATAAGACTG
VIM(F)	GACGCCATCAACACCGAGTT
VIM(R)	CTTTGTCGTTGGTTAGCTGGT

Figure 1D

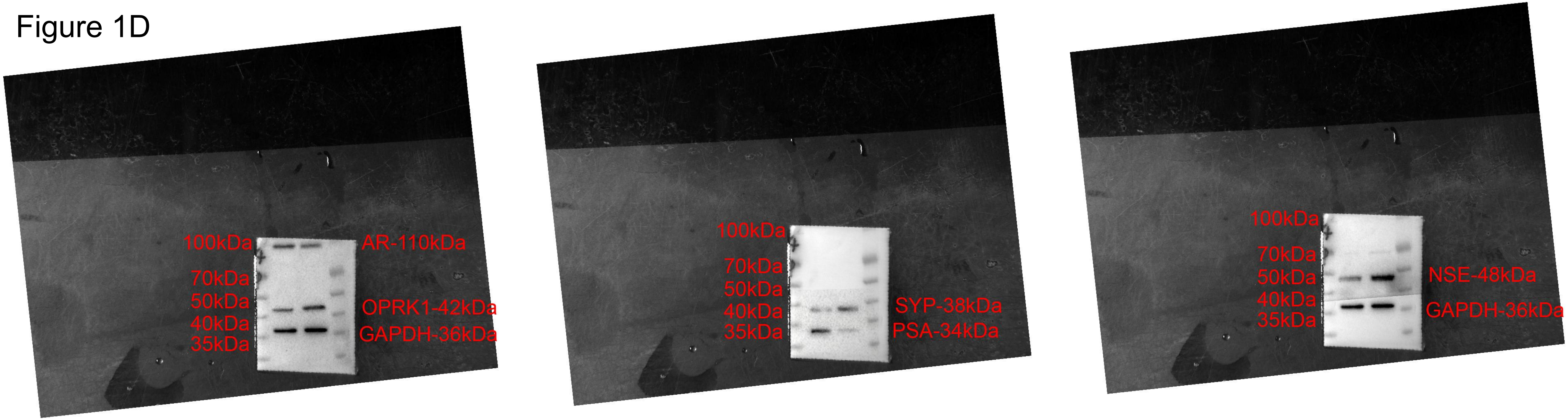

Figure 2F

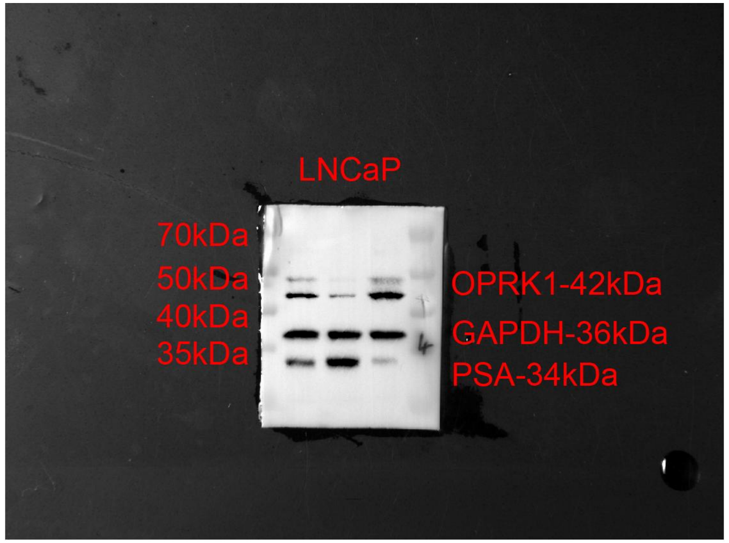

Figure 2H

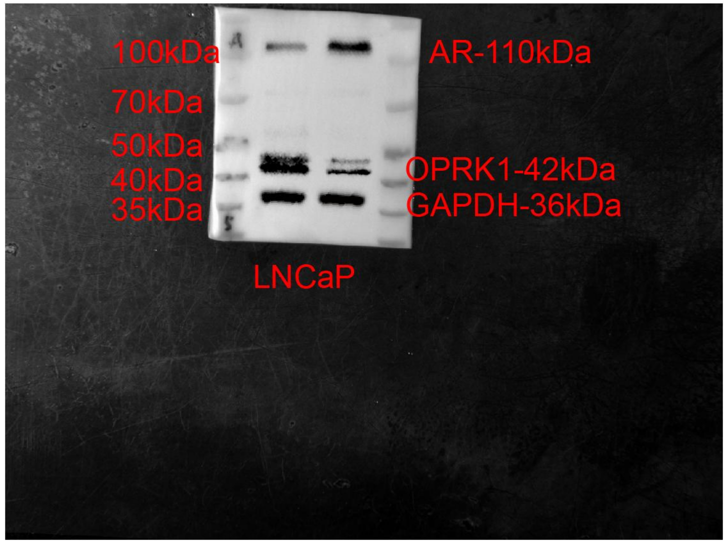

Figure 2J

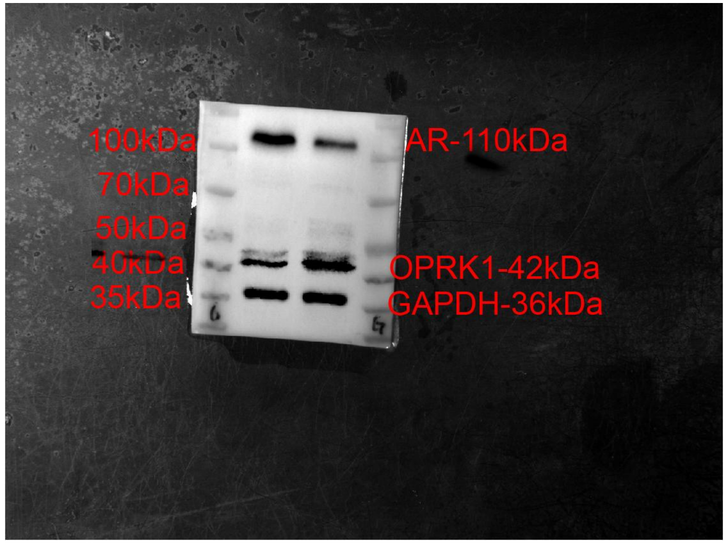

Figure 3A

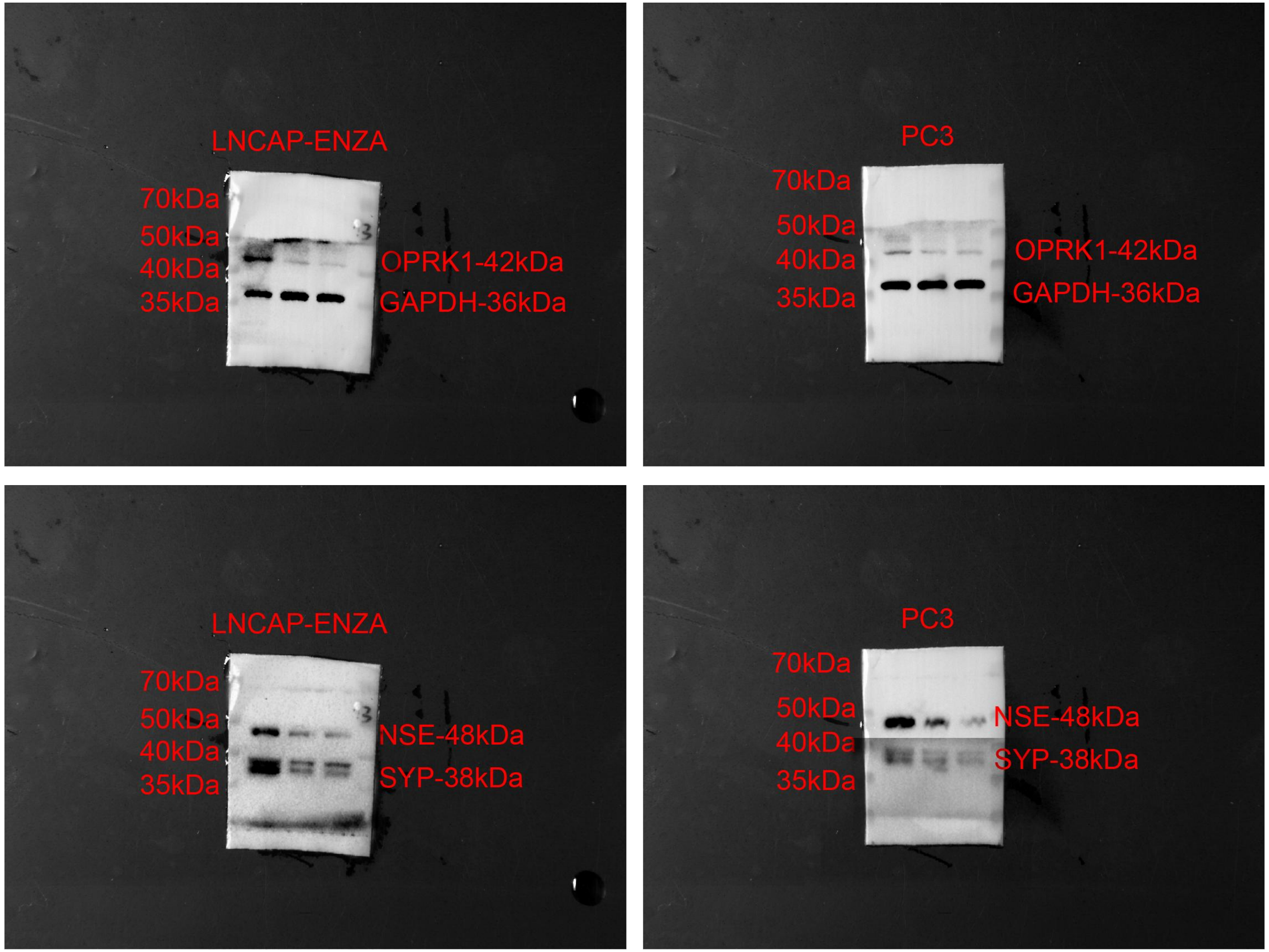

Figure 4A

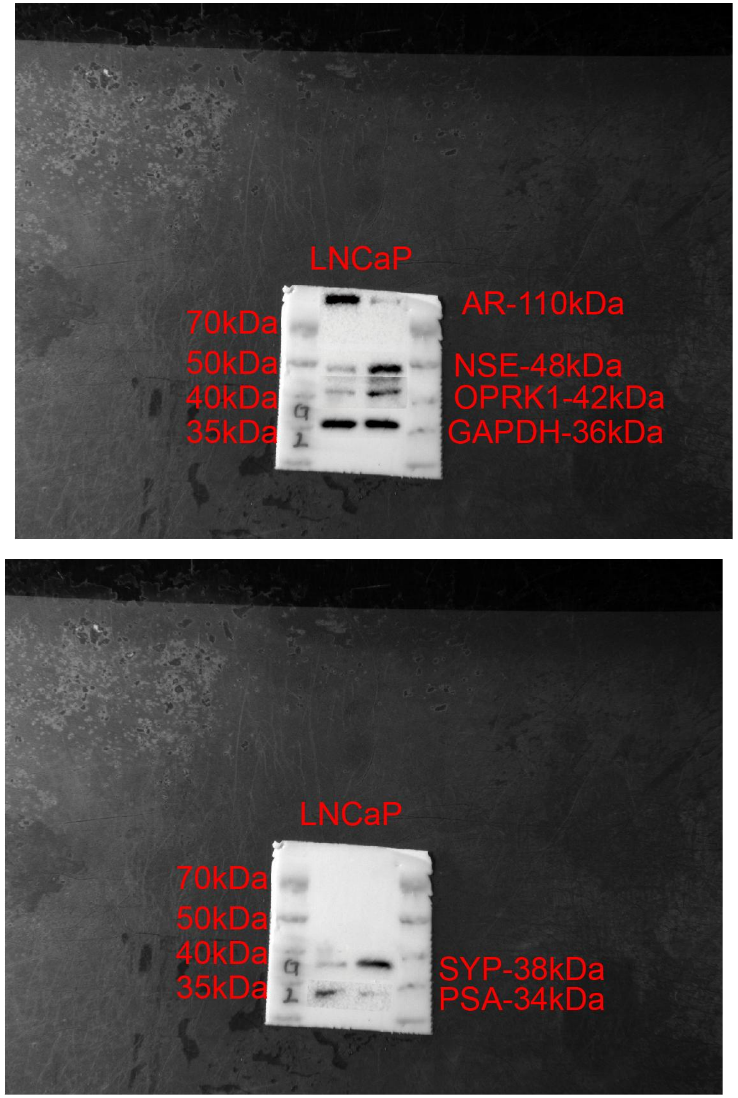

Figure 4A

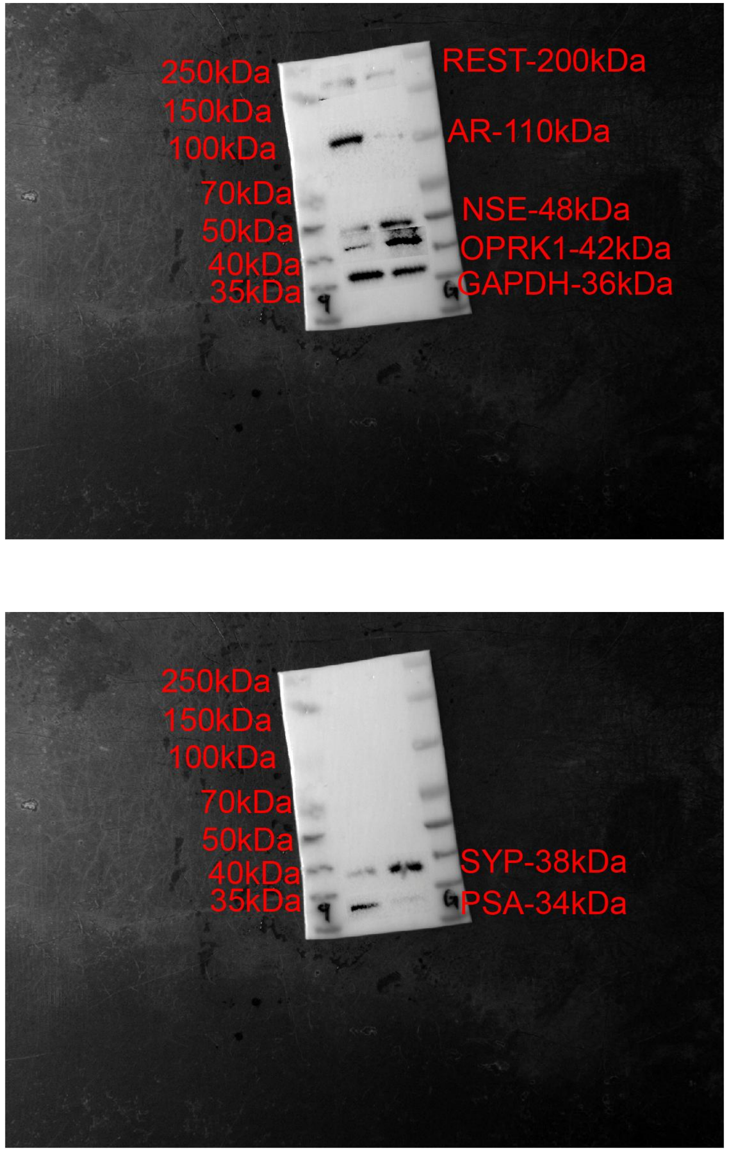

Figure 5B

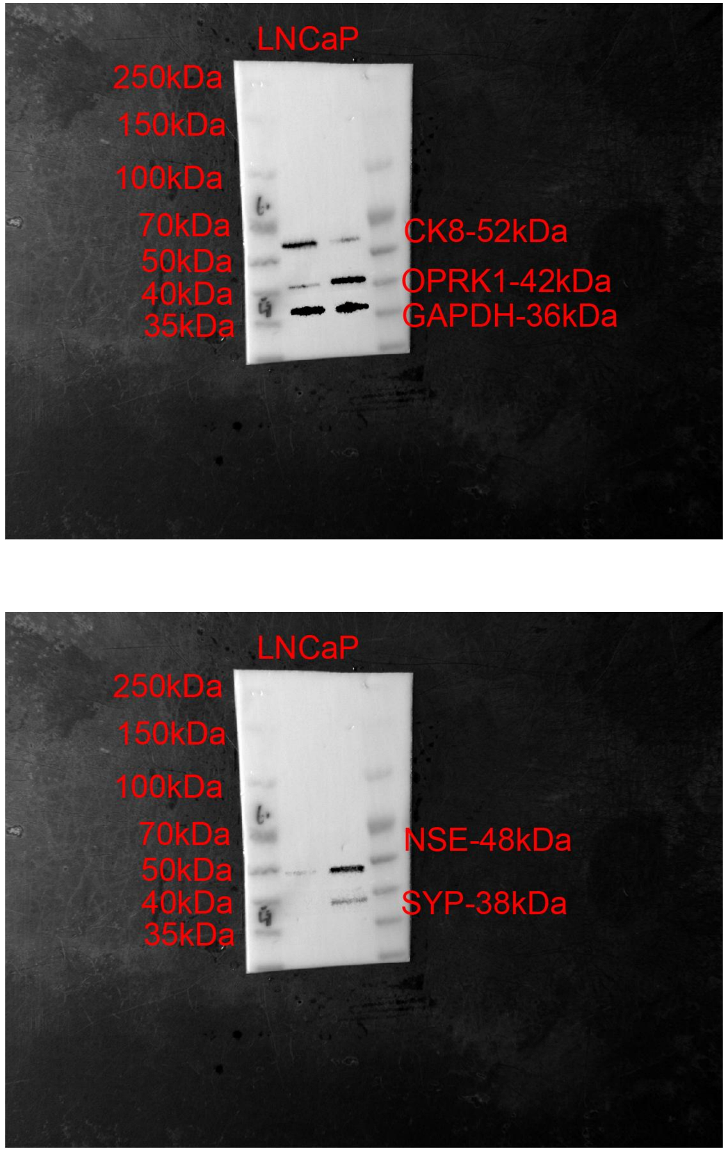

Figure 5C

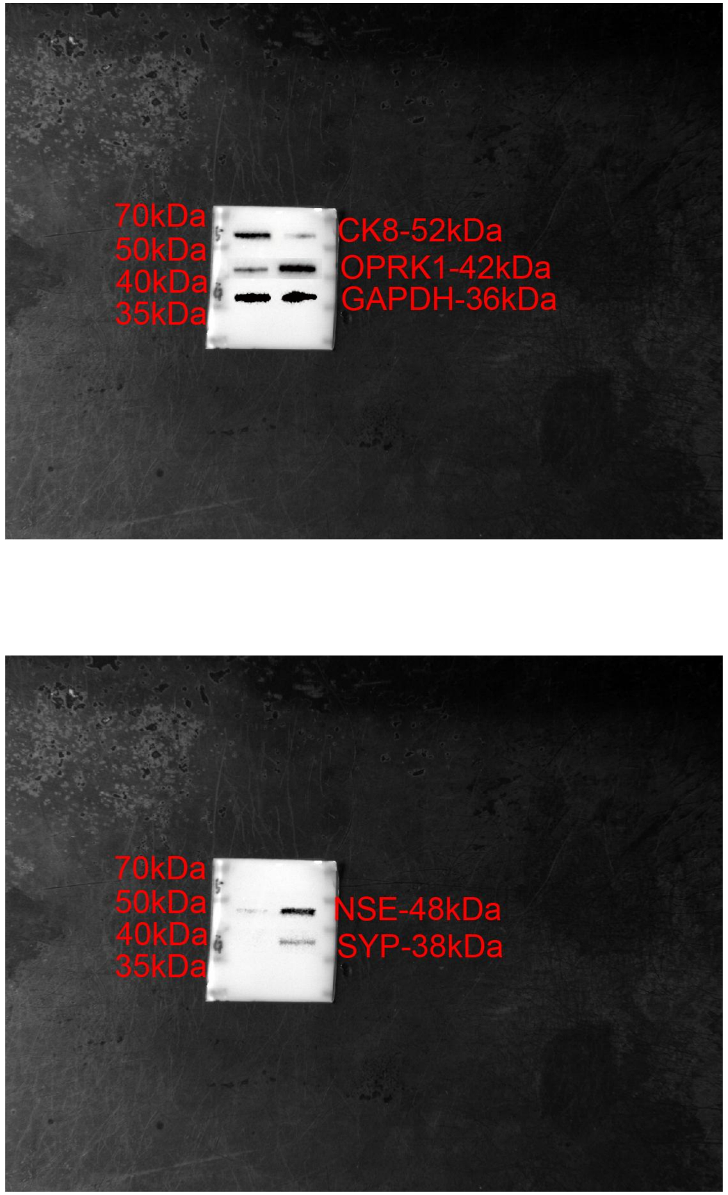

Figure 5D

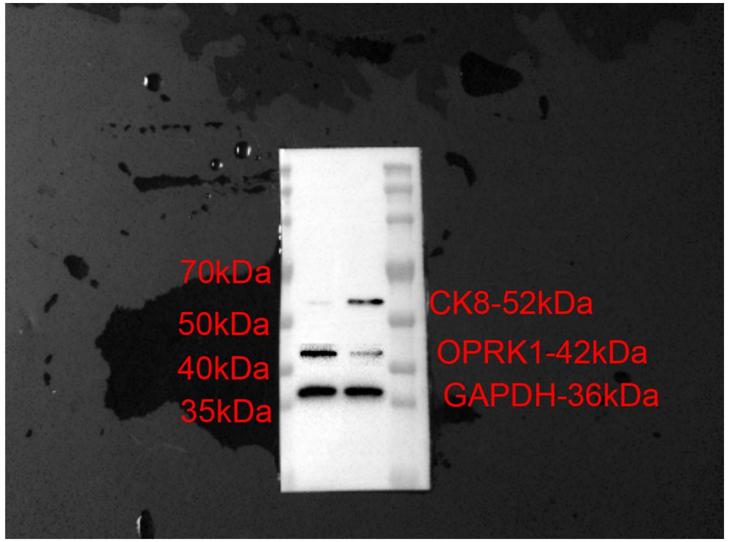

Figure 5E

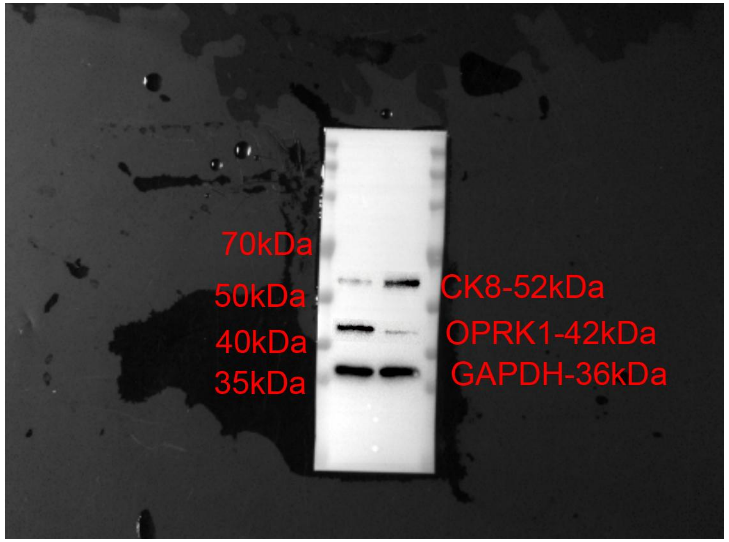

Figure 5L

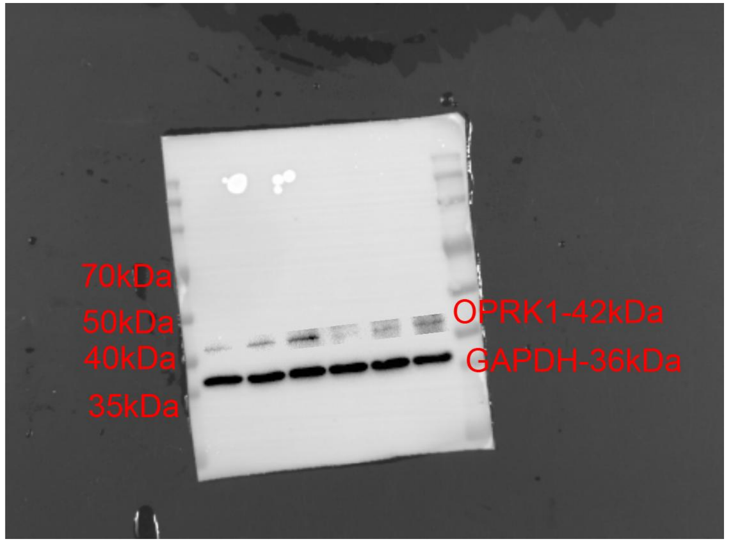

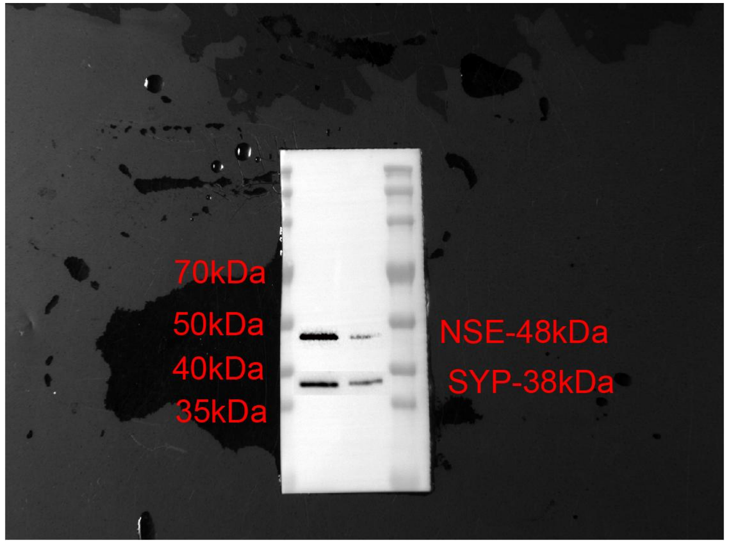

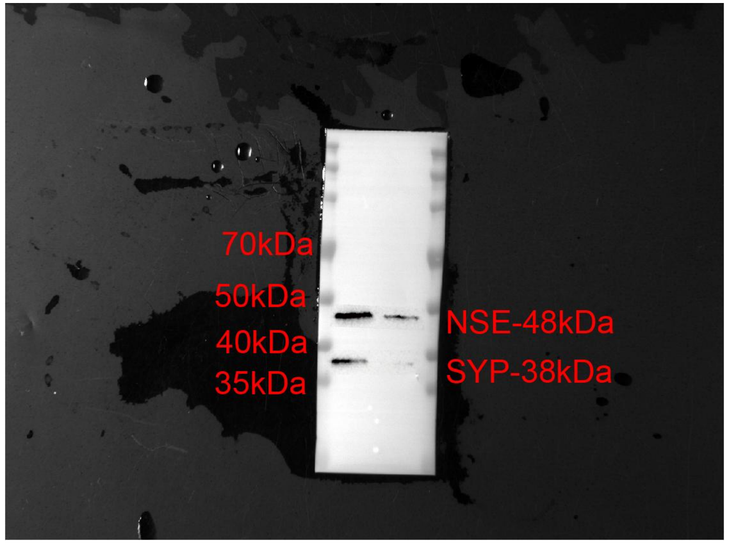

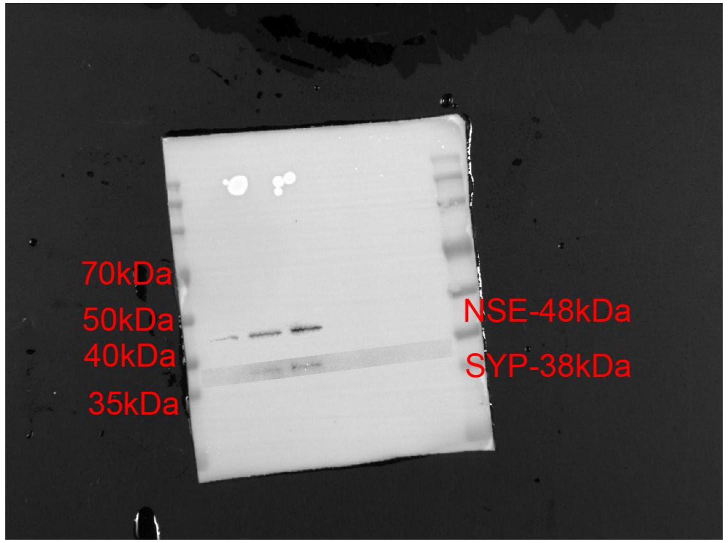

Figure 6C

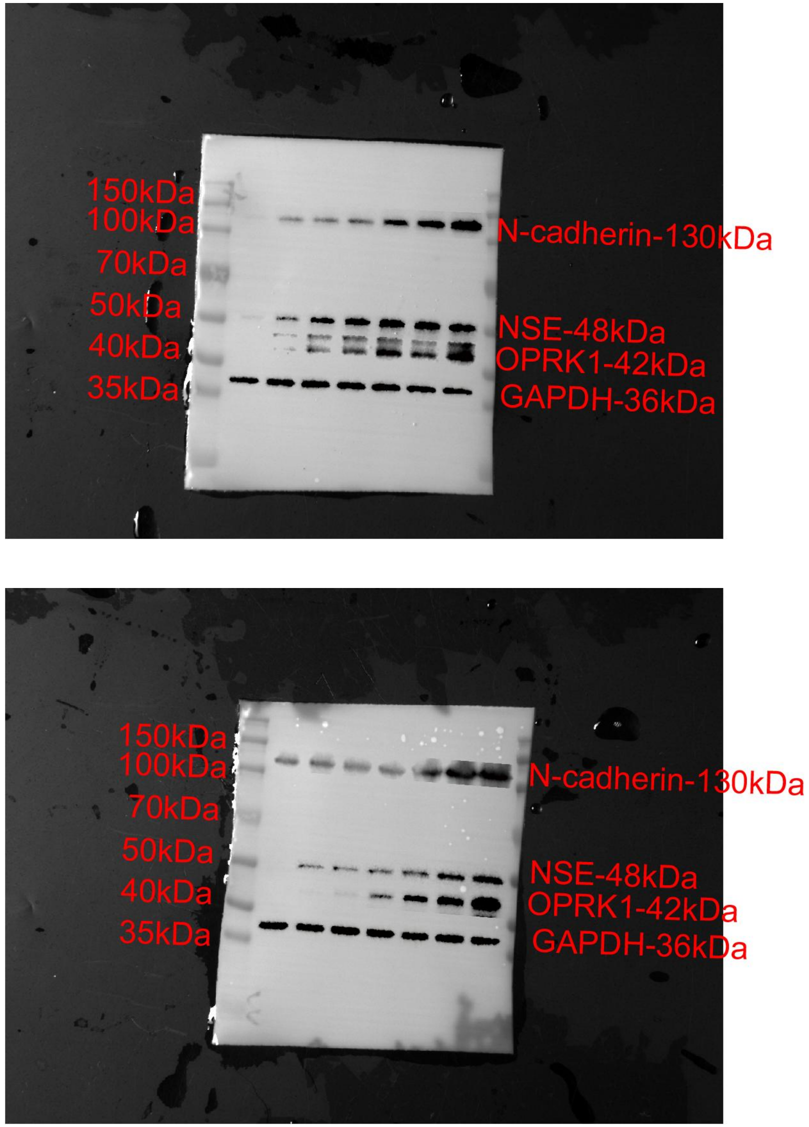

Figure 6D

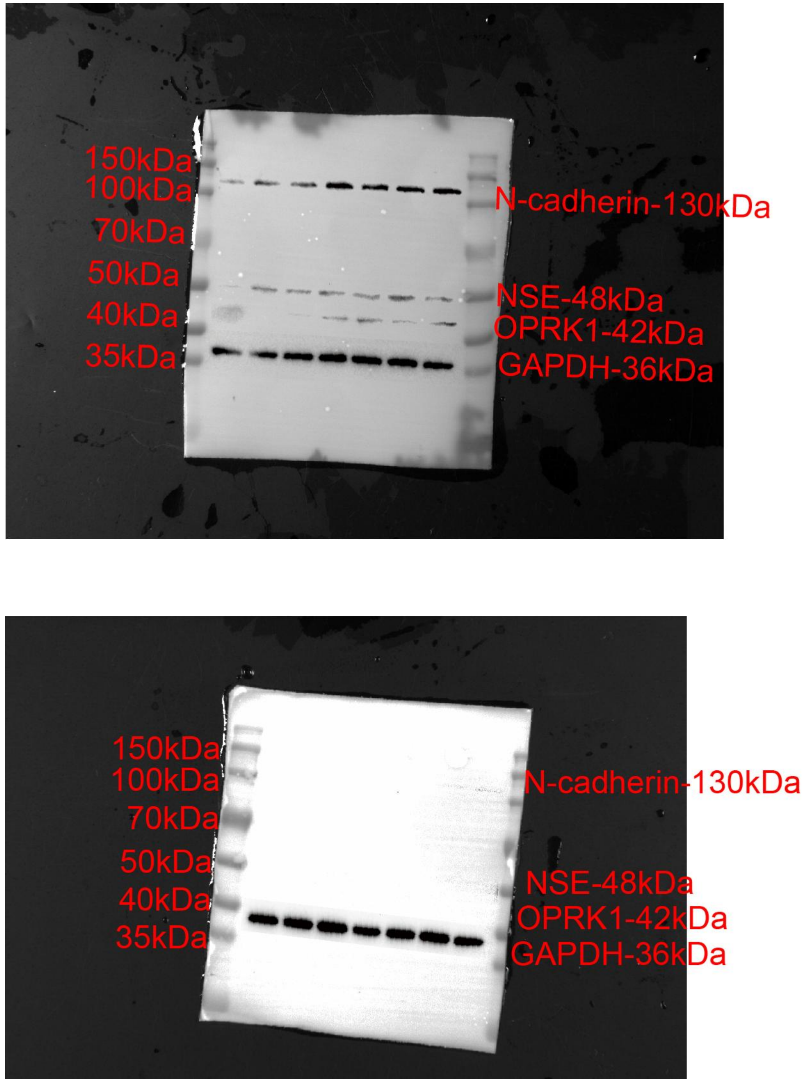

Figure 6H

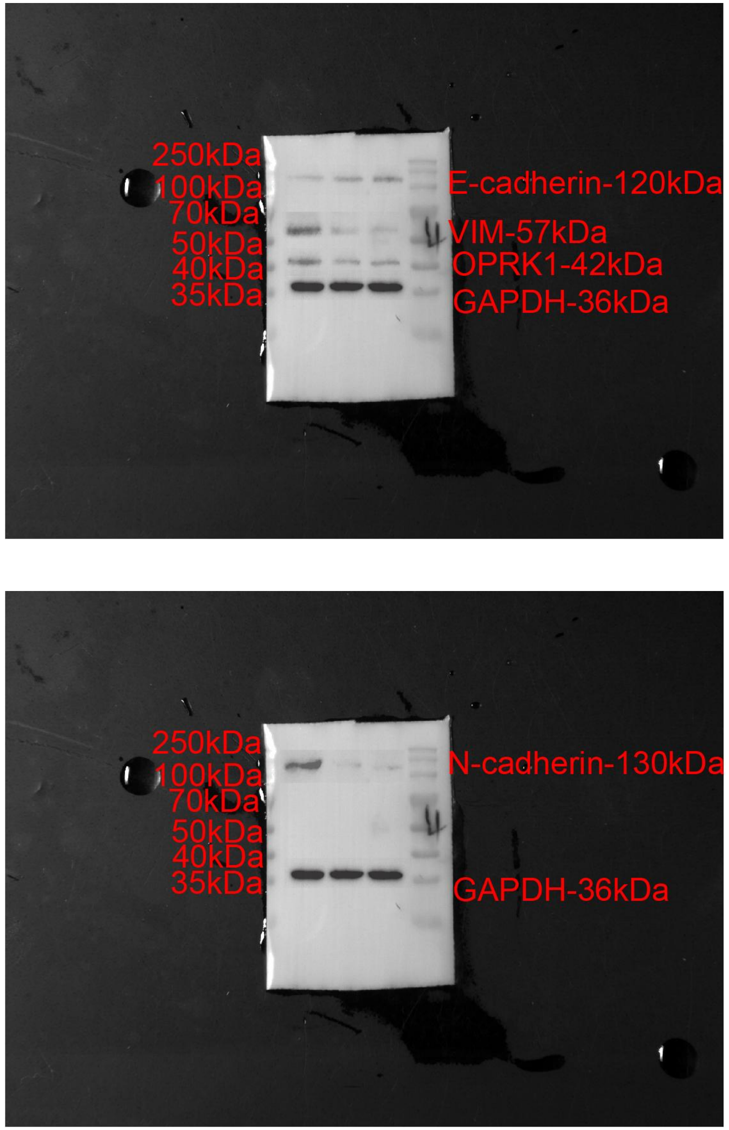

Figure 6H

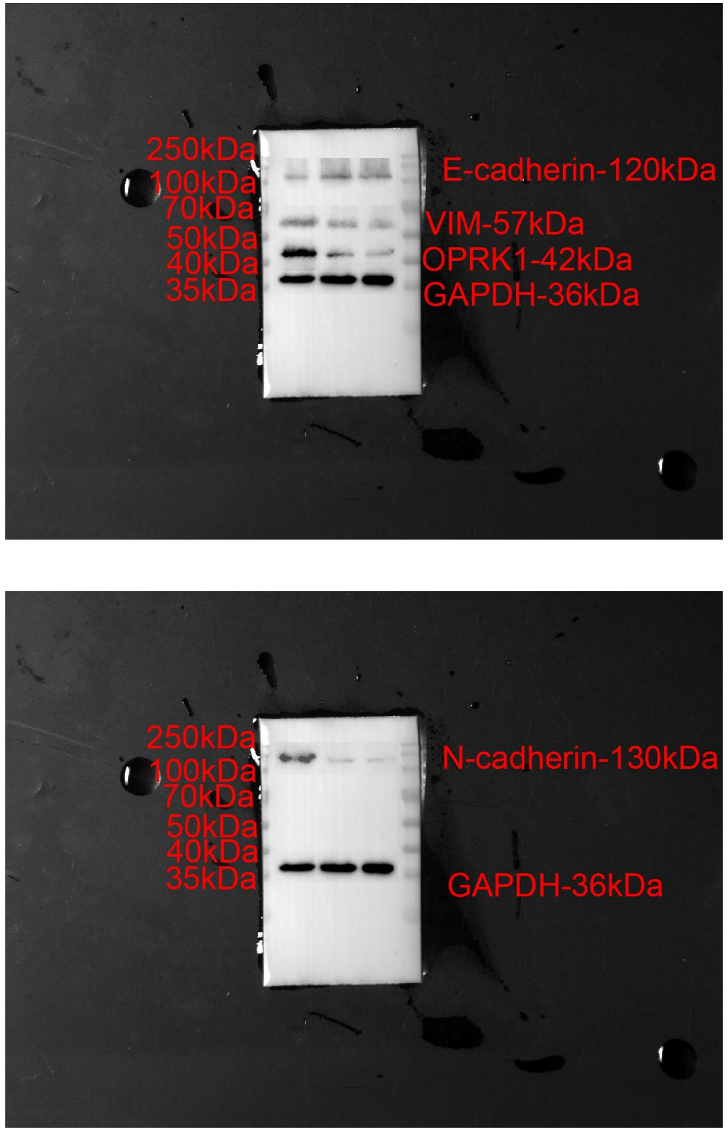

Figure 6J

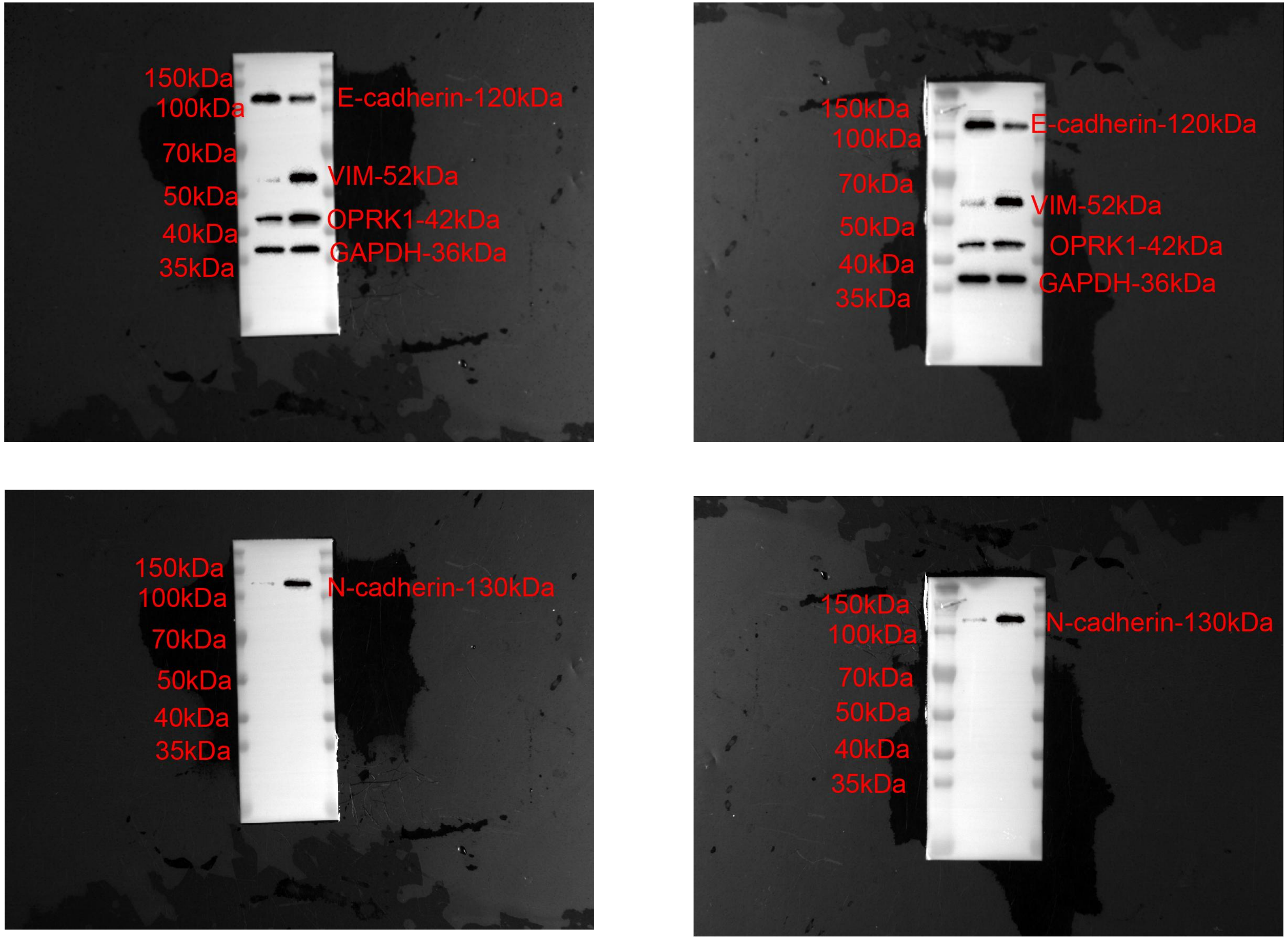

Figure 7E

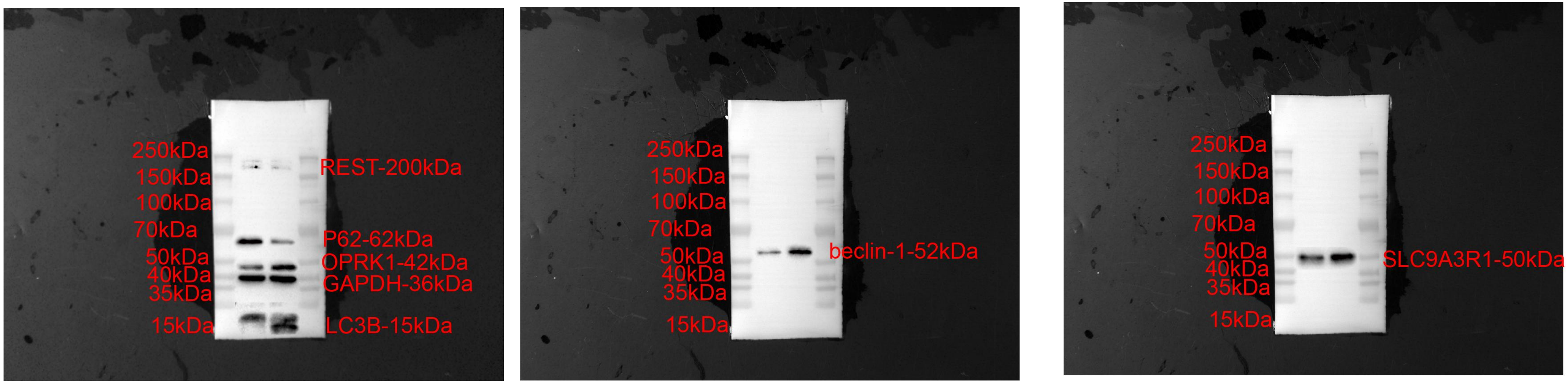

Figure 7F

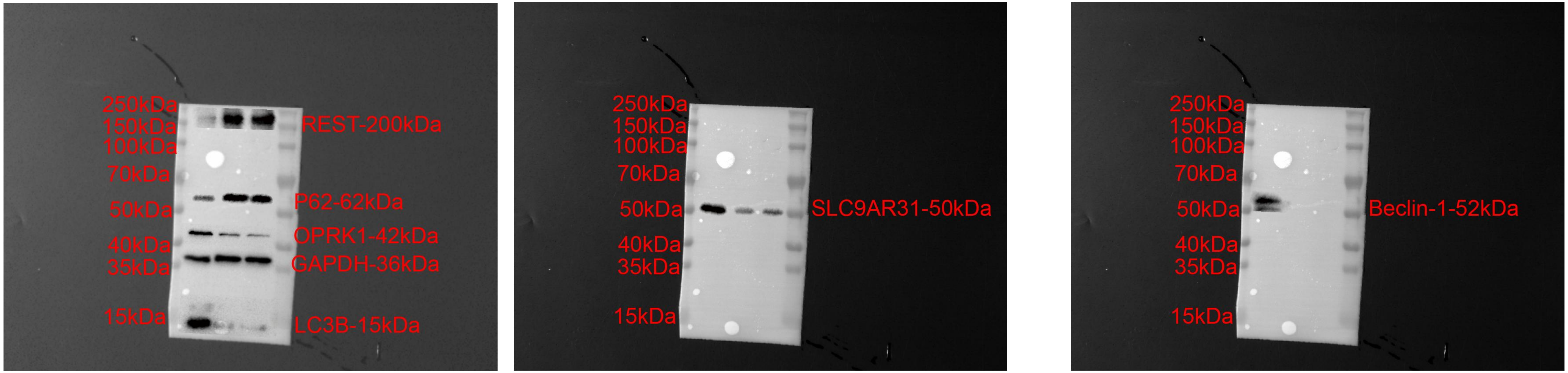

Figure 7I

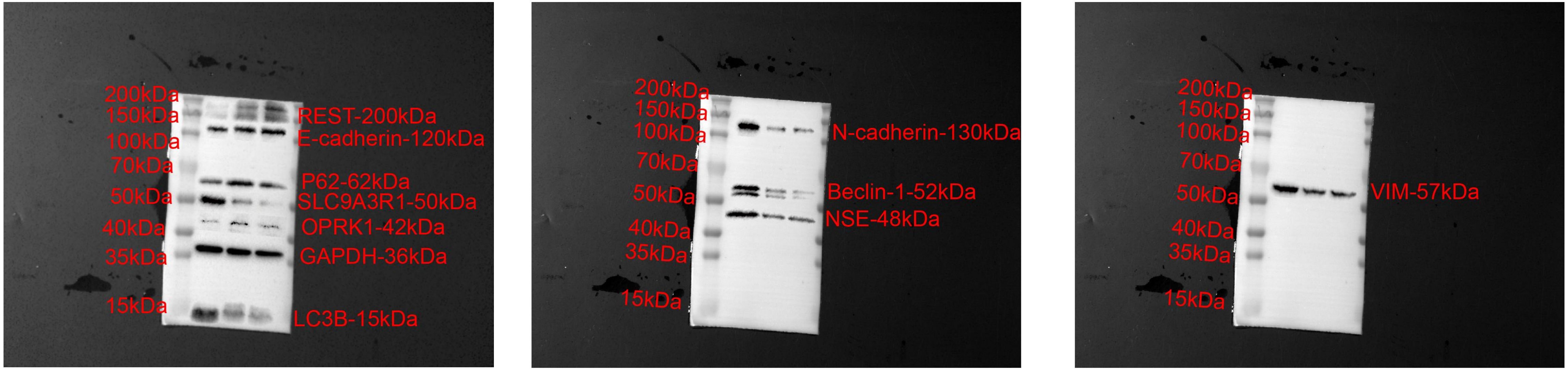

Figure 7J

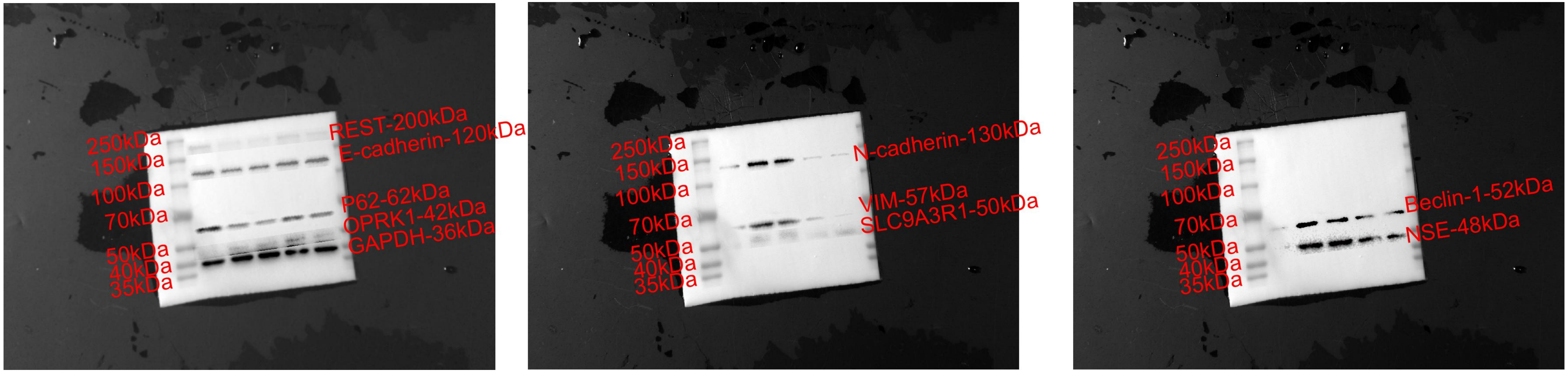

Figure 7L

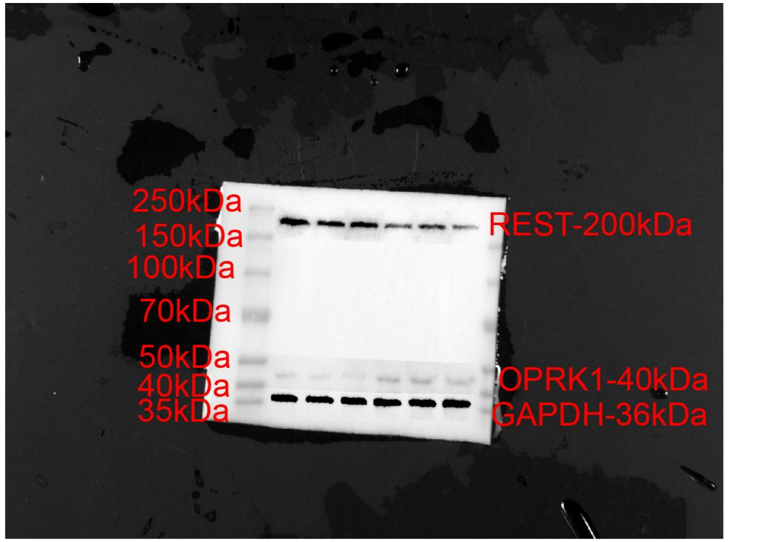

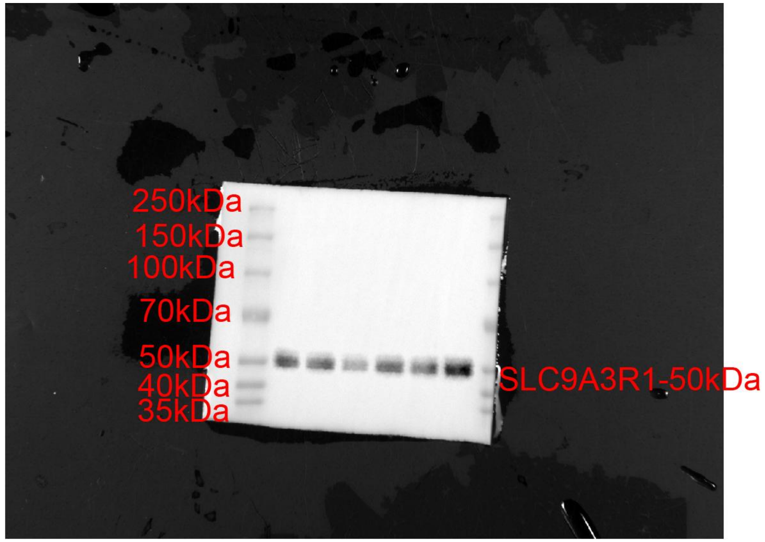

Figure 7M

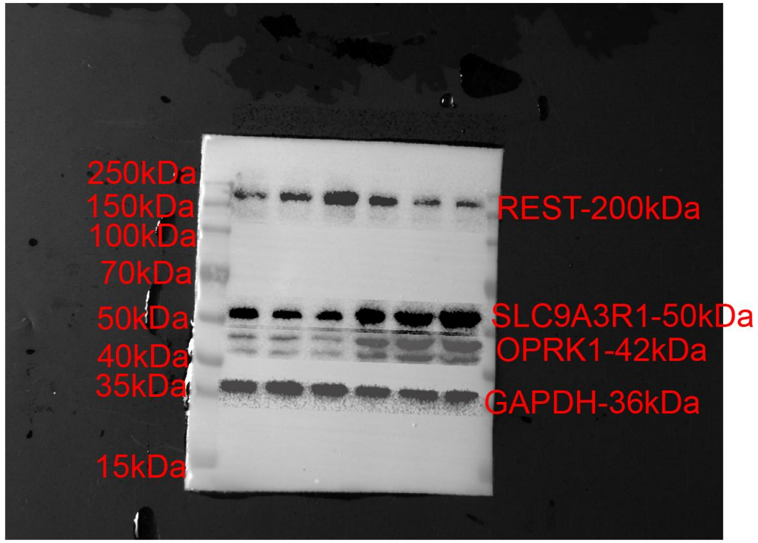

Figure 7N

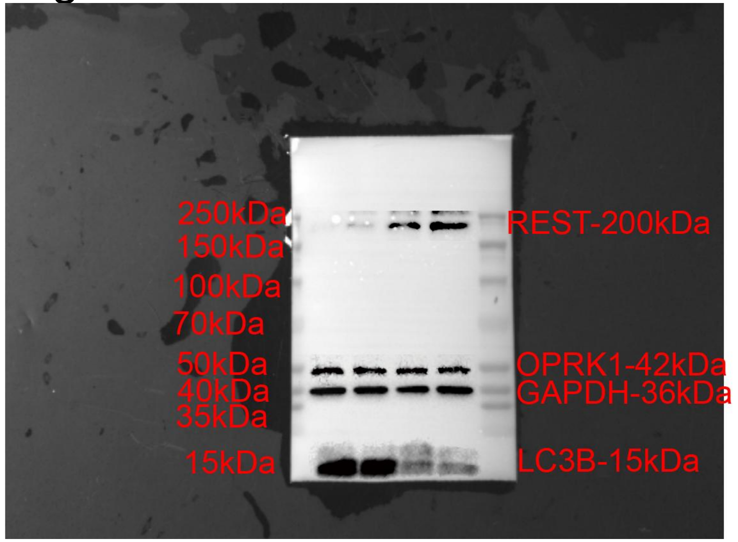

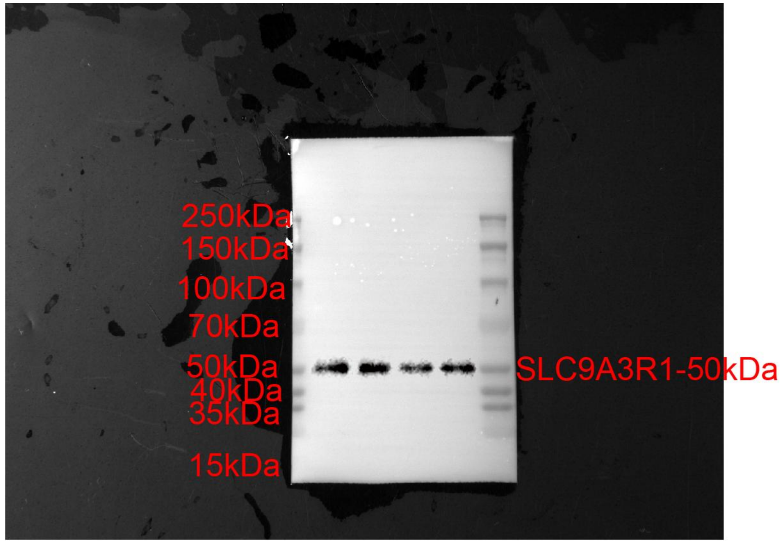

Figure 7O

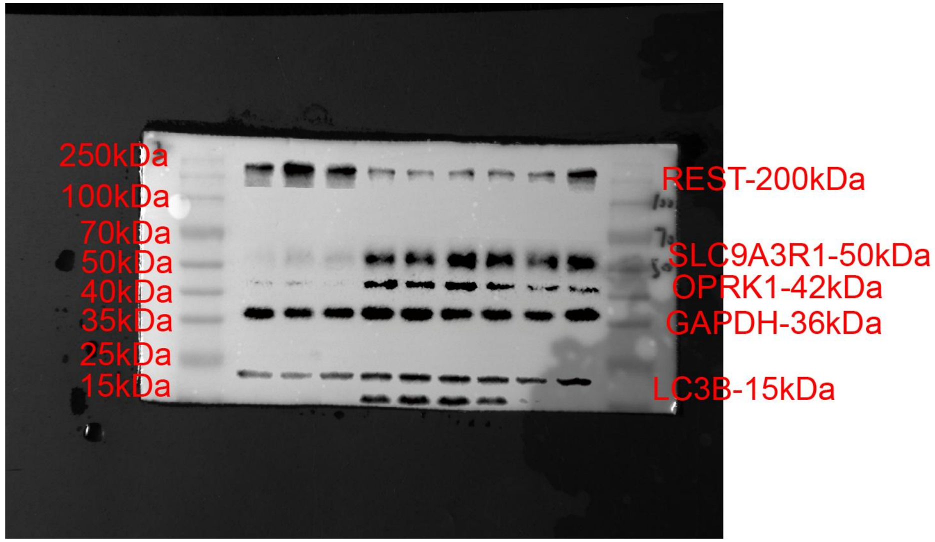

Figure 8C

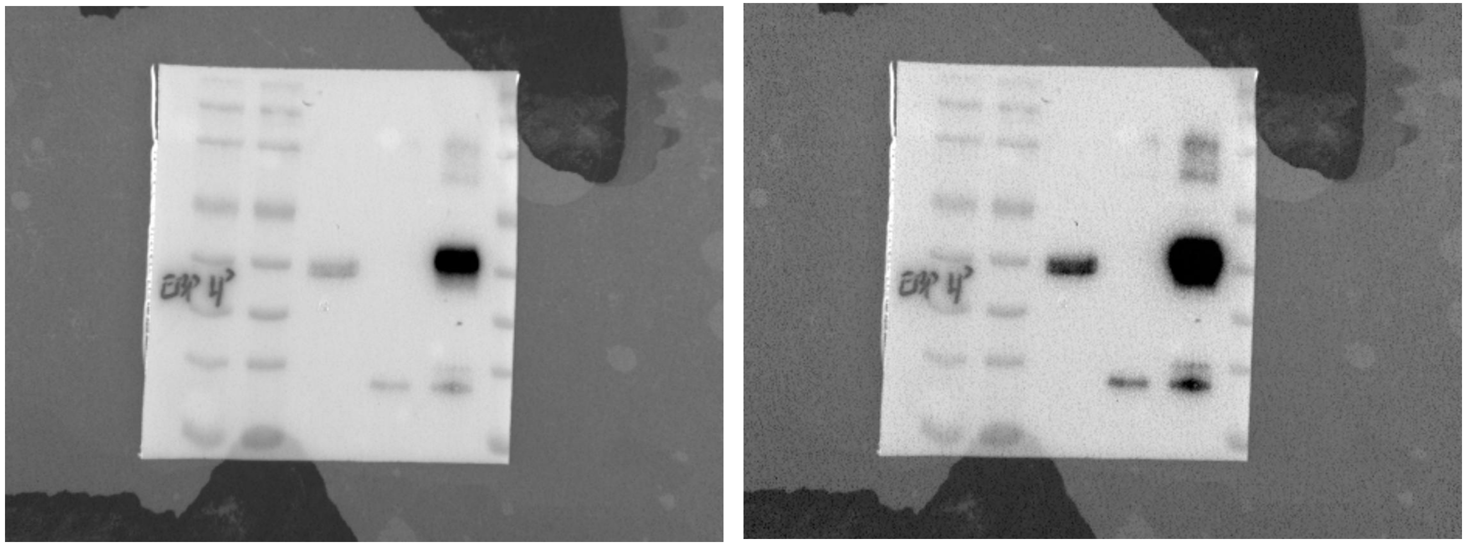

Figure 8D

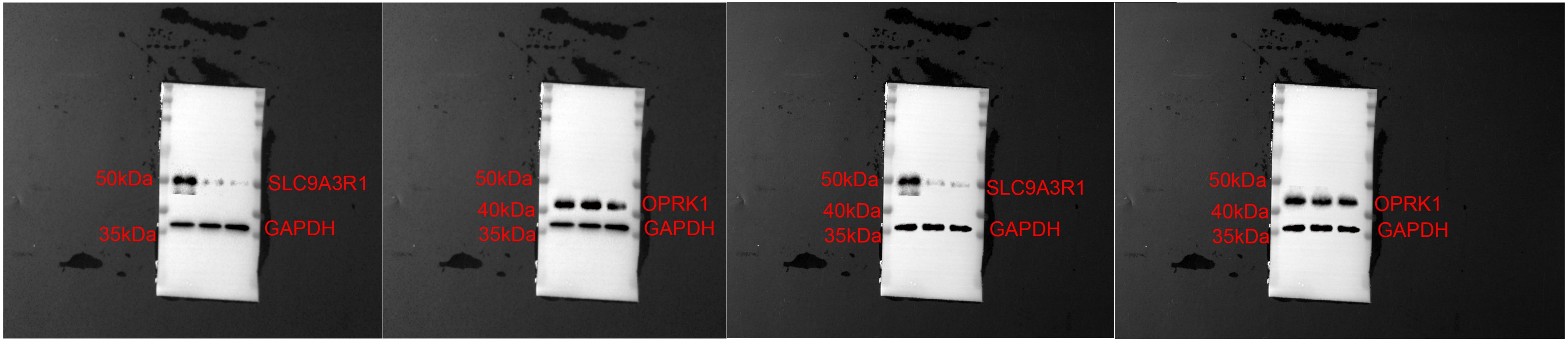

Figure 8E

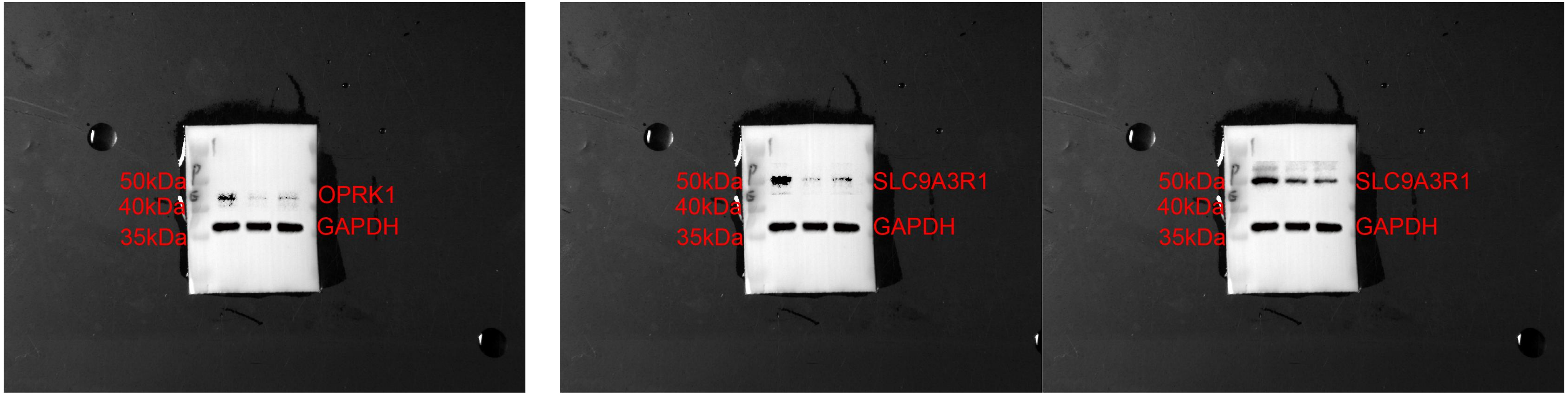

Figure 8G

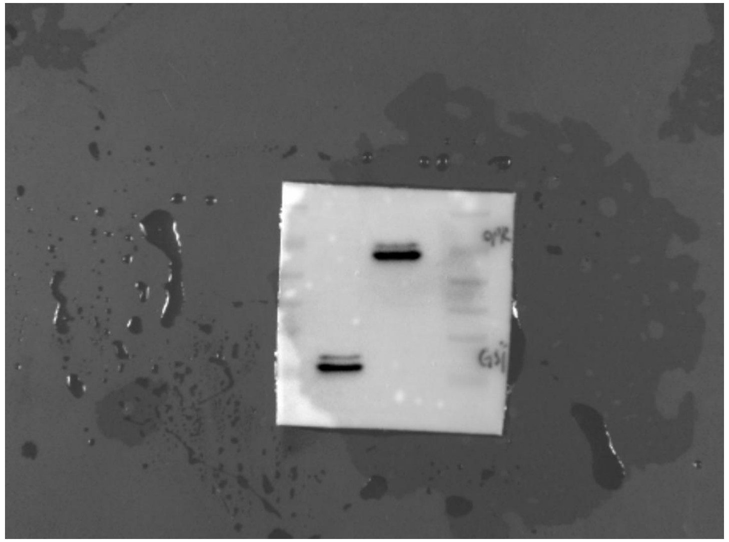

Figure 8I

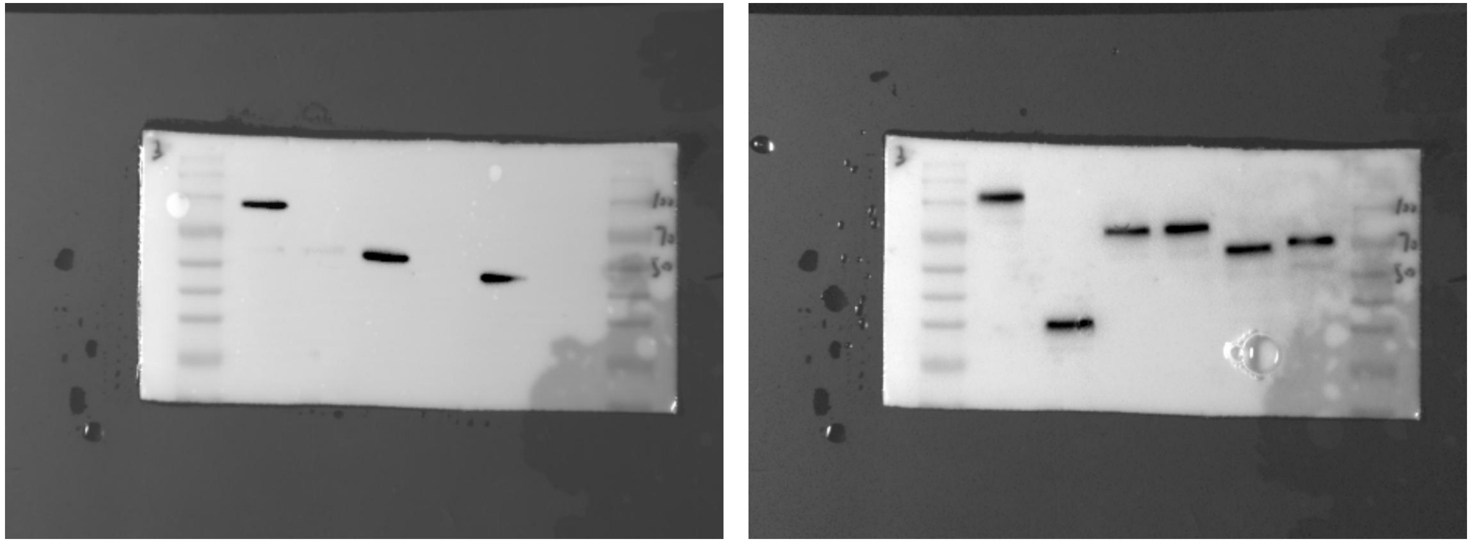

Figure 8J

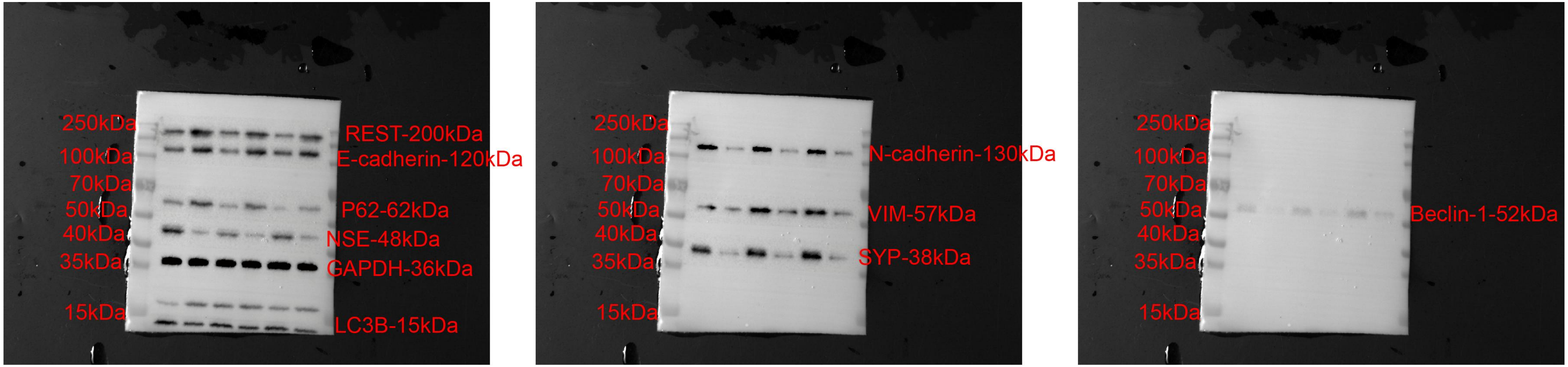

Figure 8L

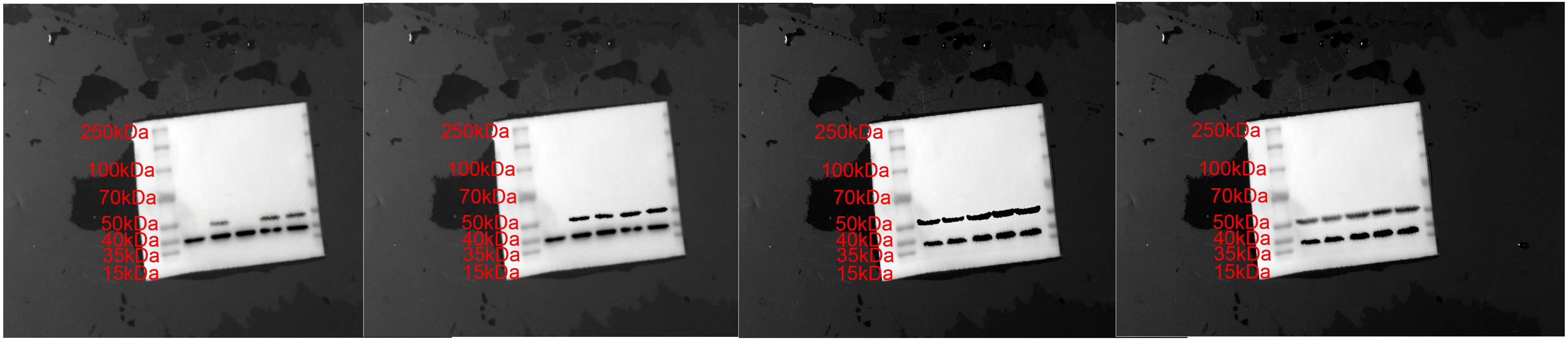

Figure 8M

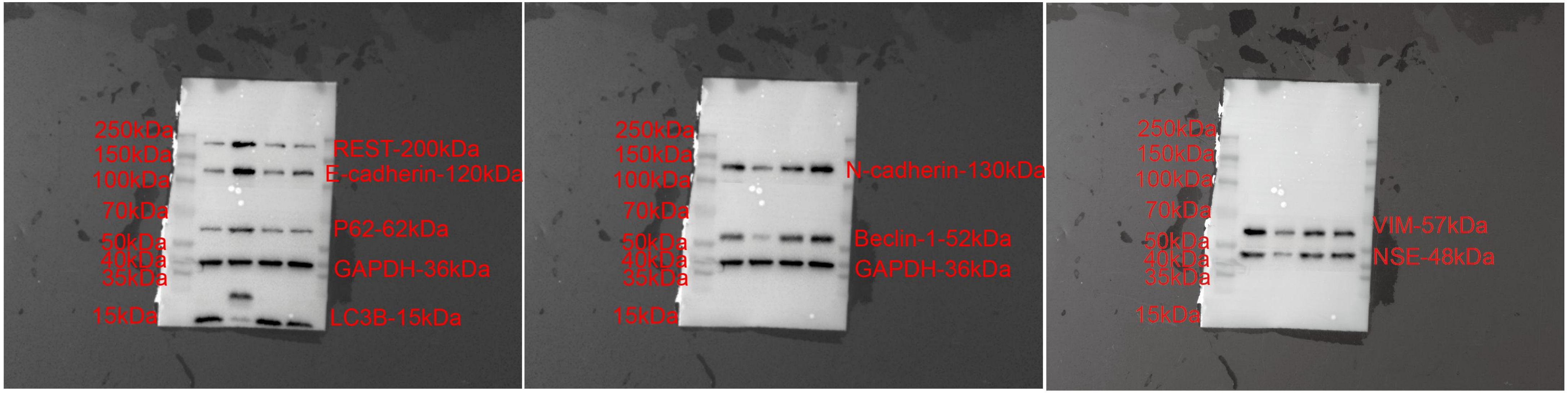

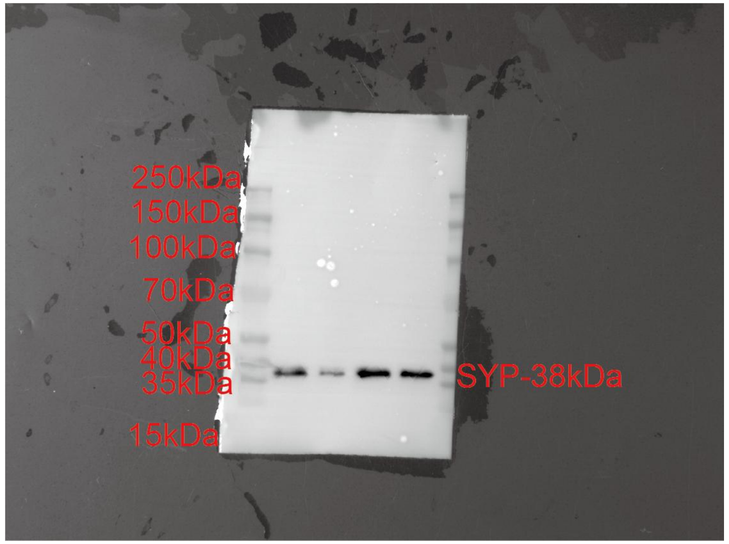

Figure 9B

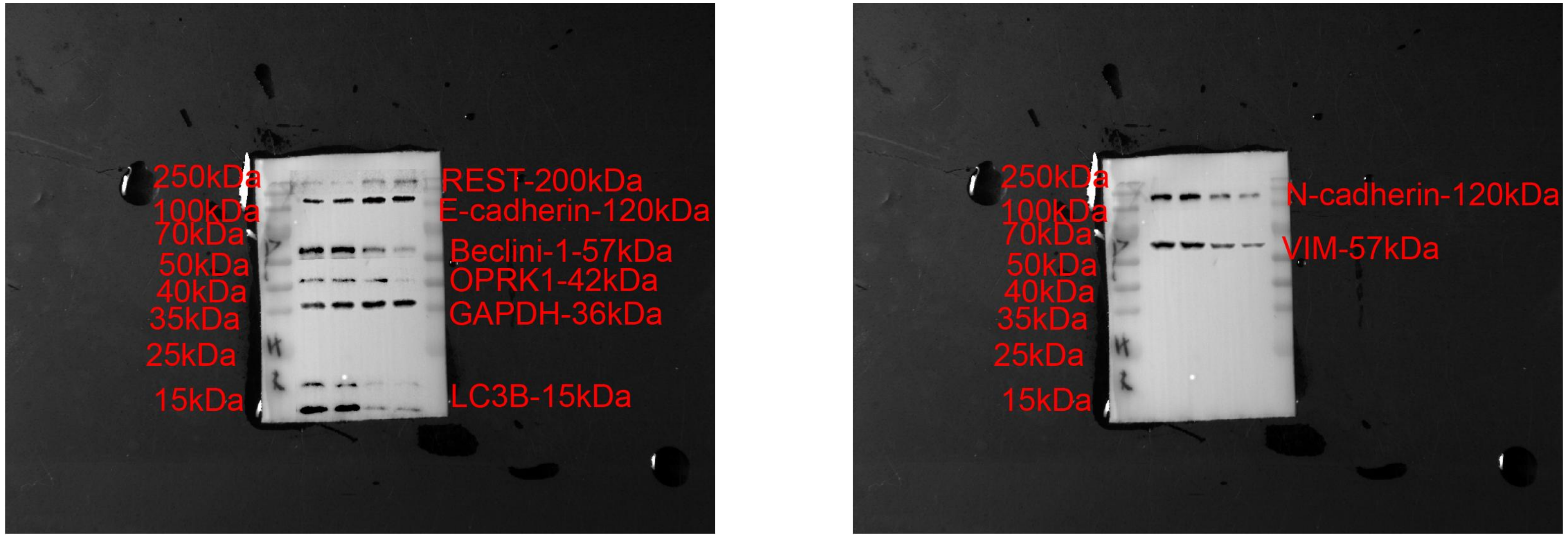

Figure 9E

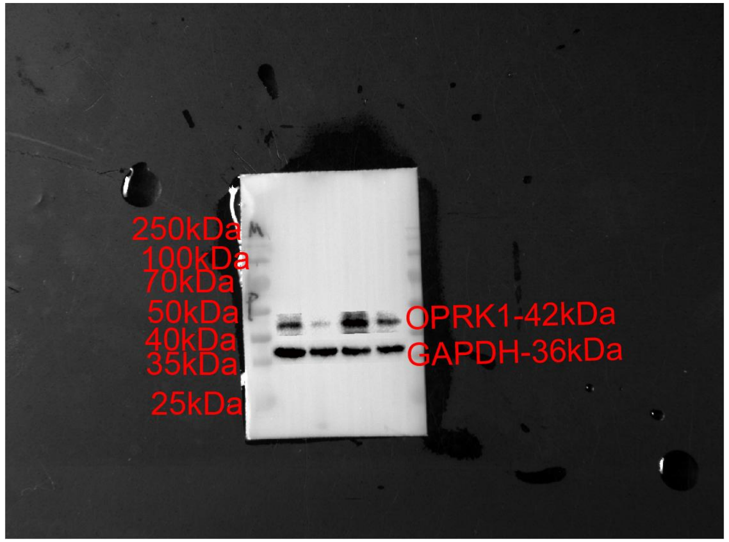

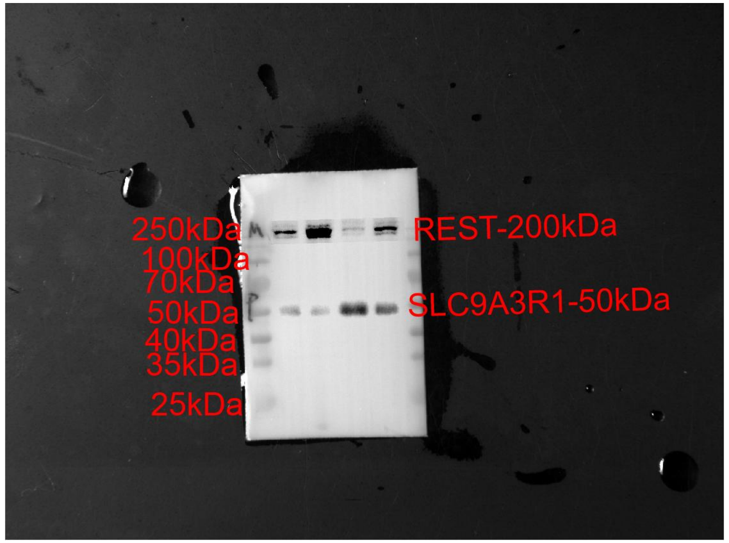

Figure 9J

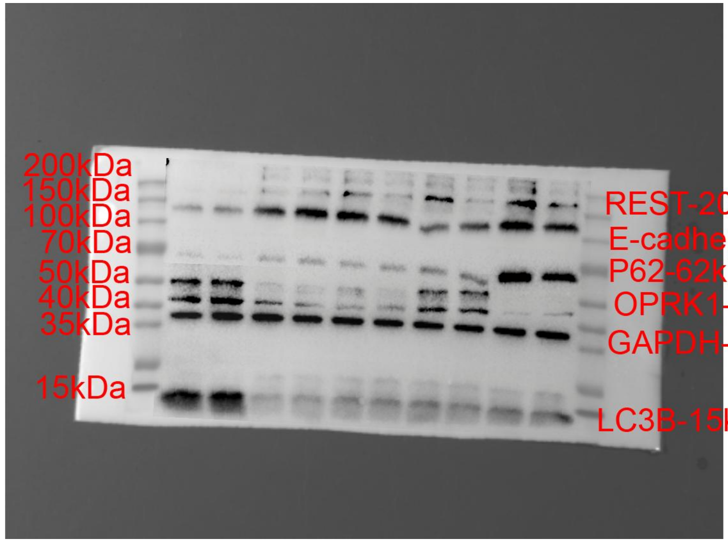

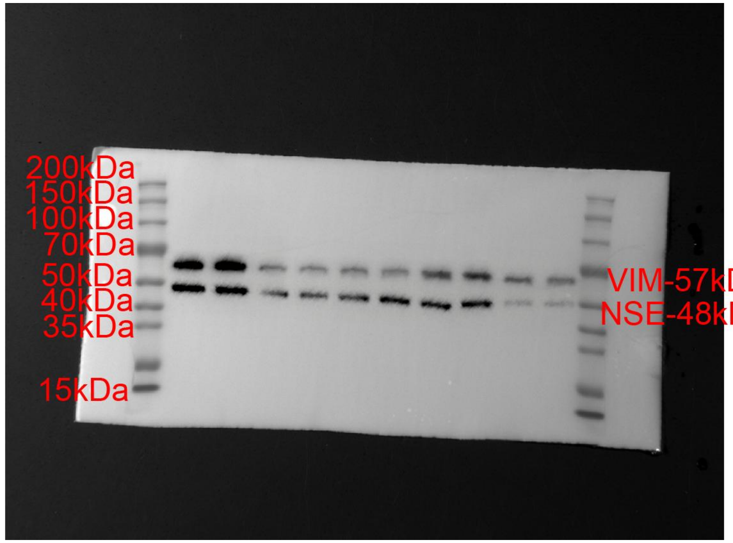

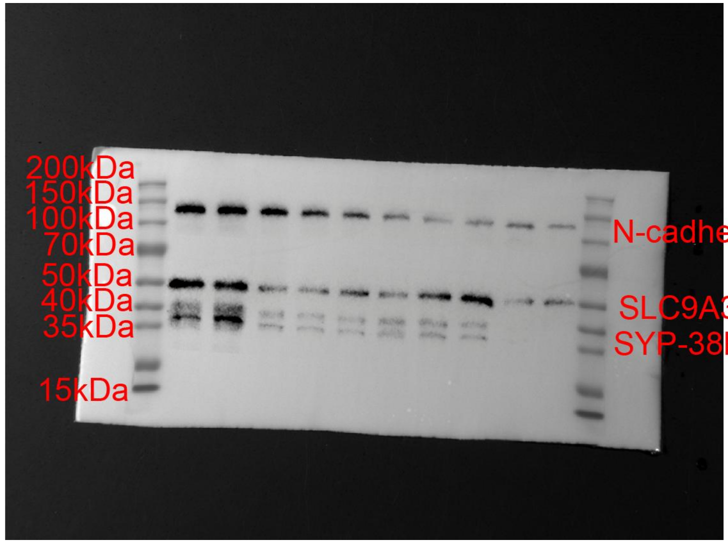

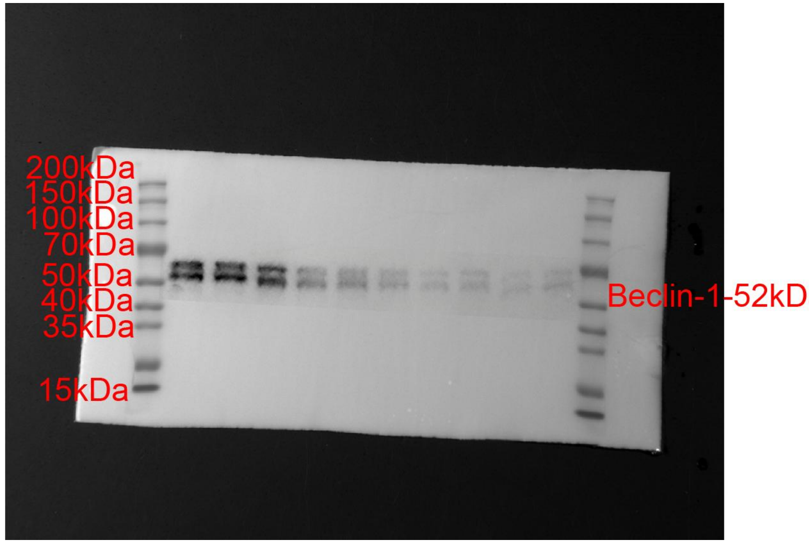

Figure S2D

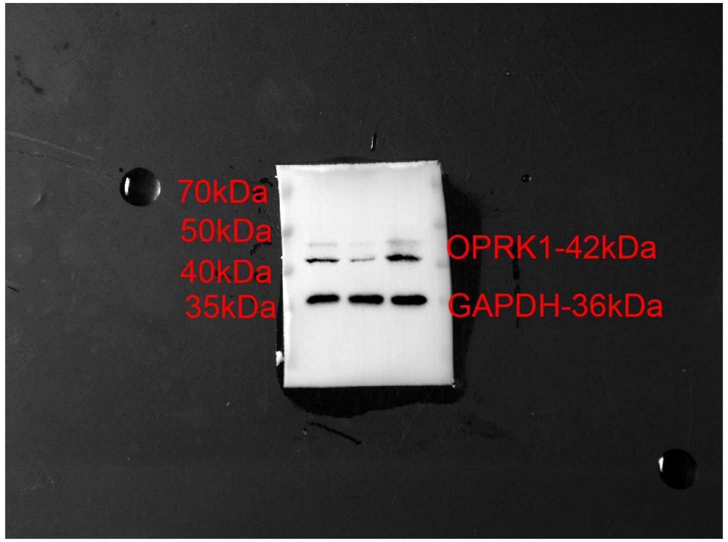

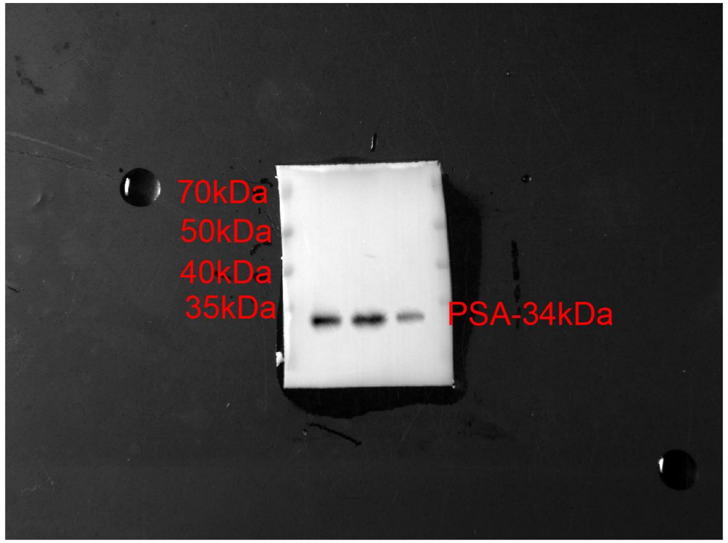

Figure S2F

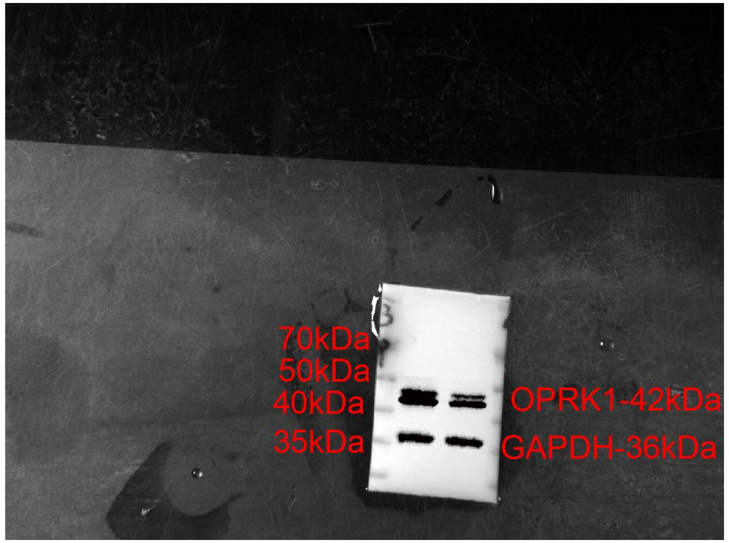

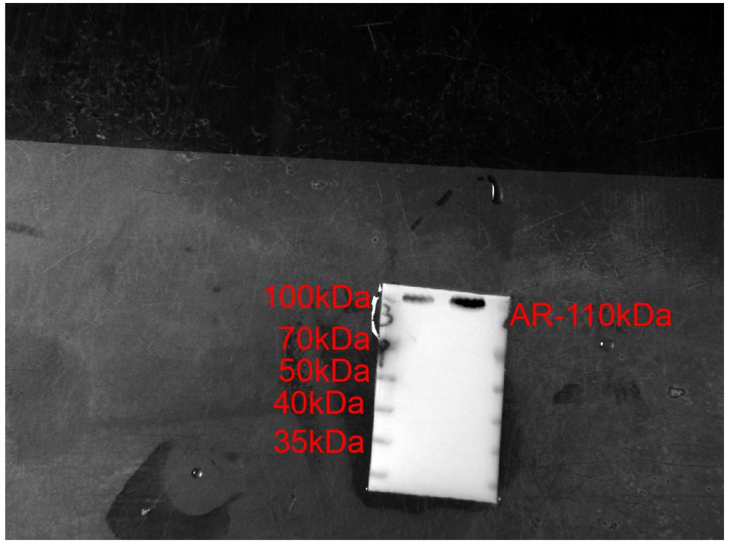

Figure S2H

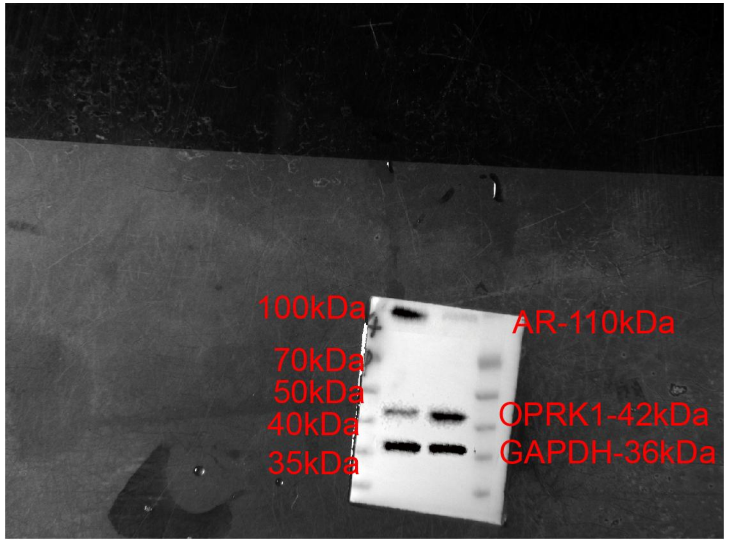

Figure S3B

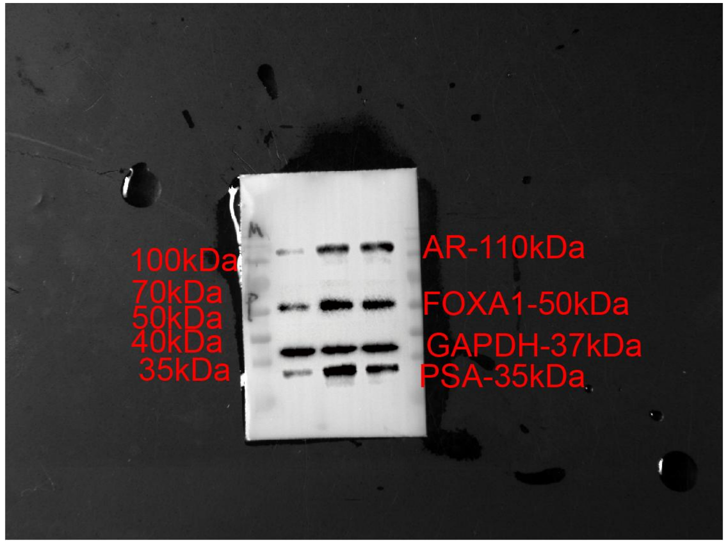

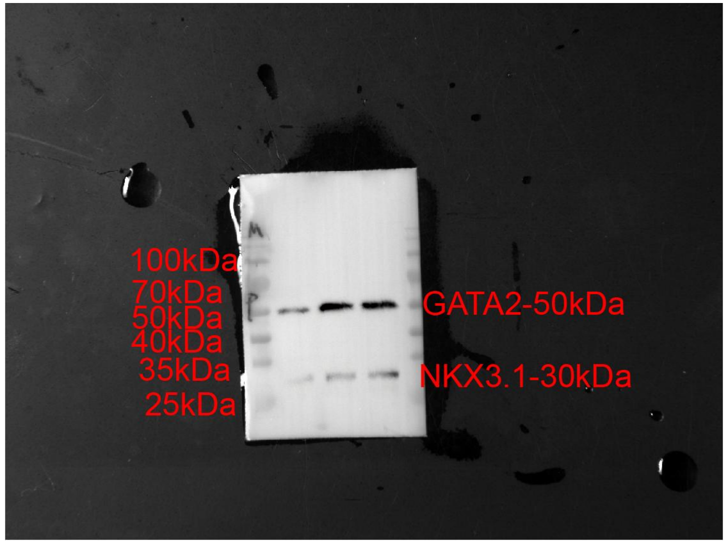

Figure S4A

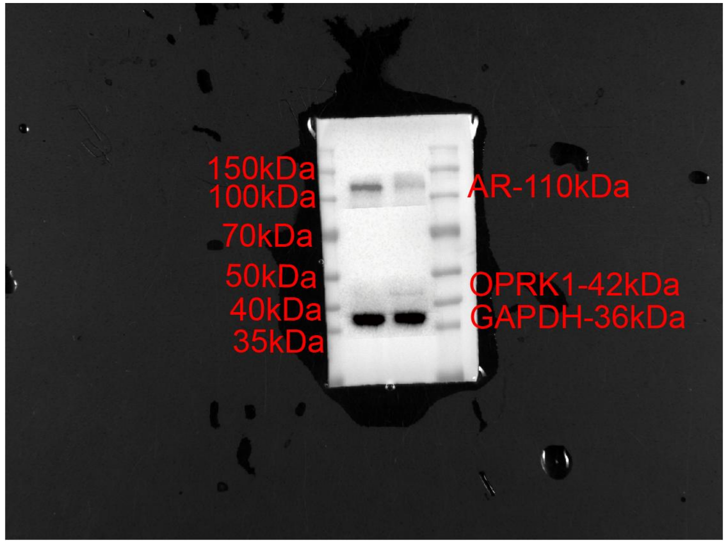

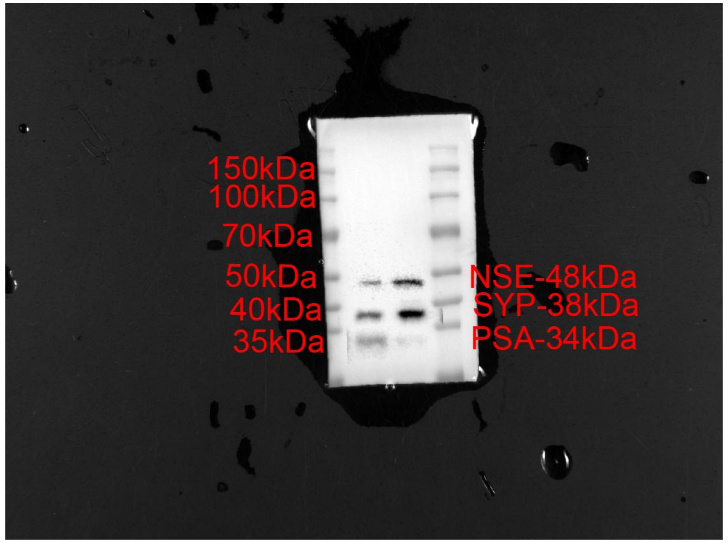

Figure S6G

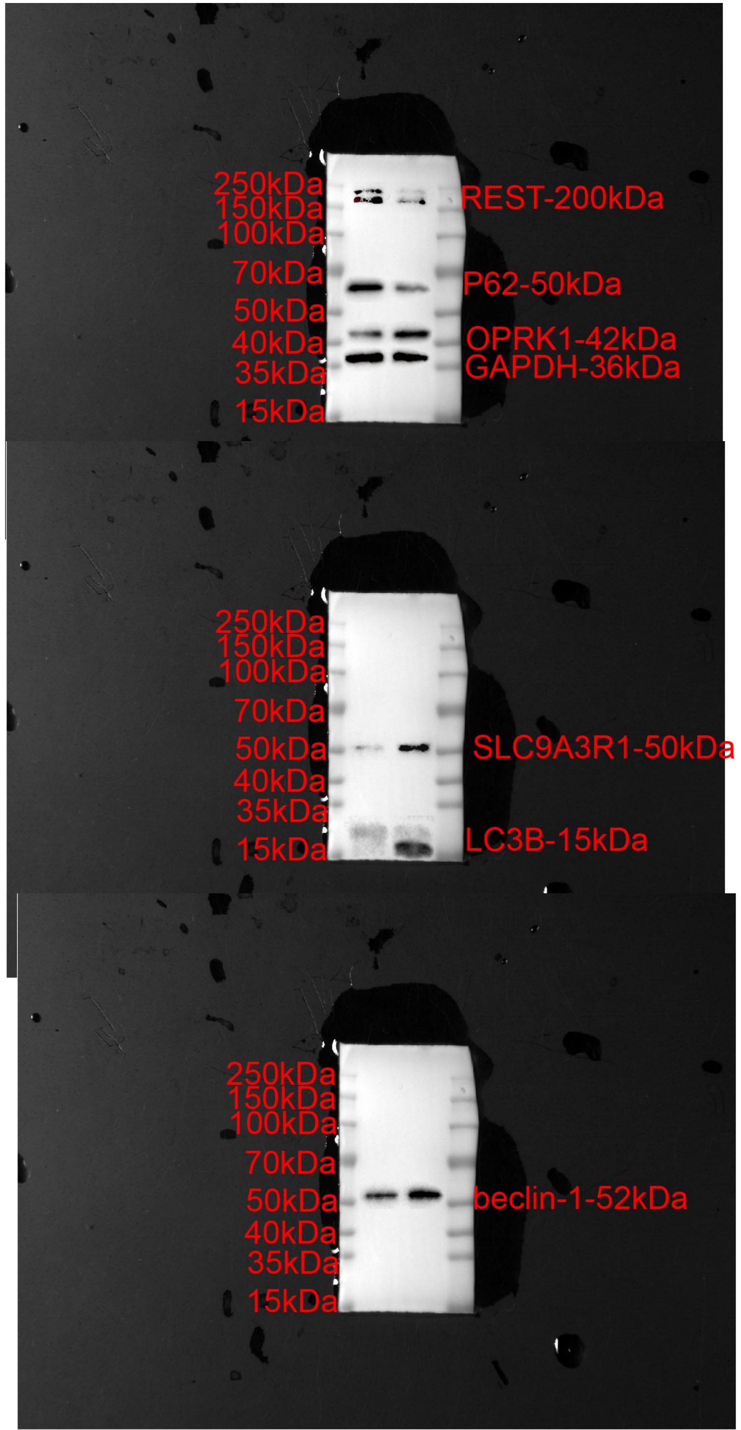

Figure S6H

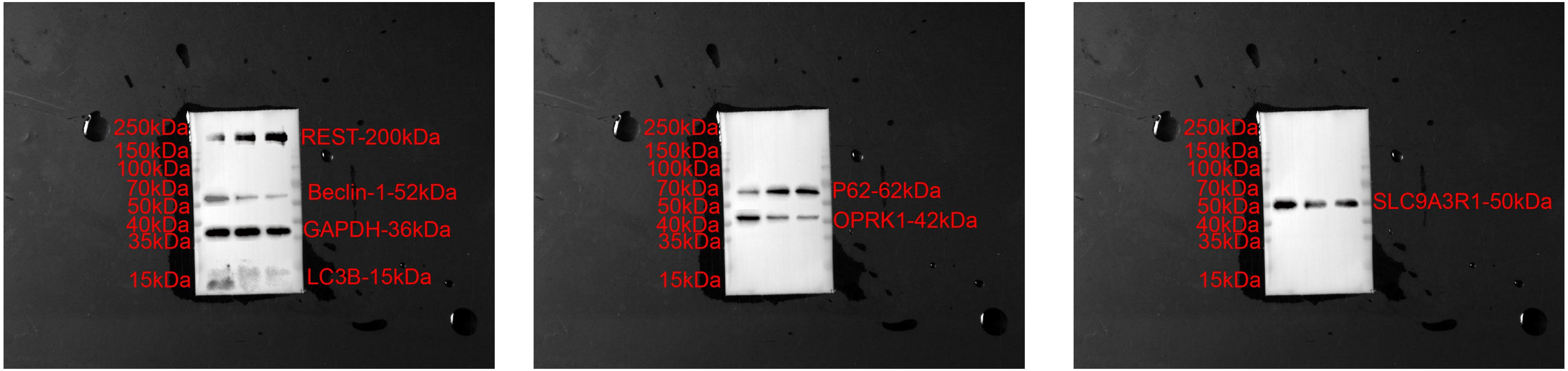

Figure S6L

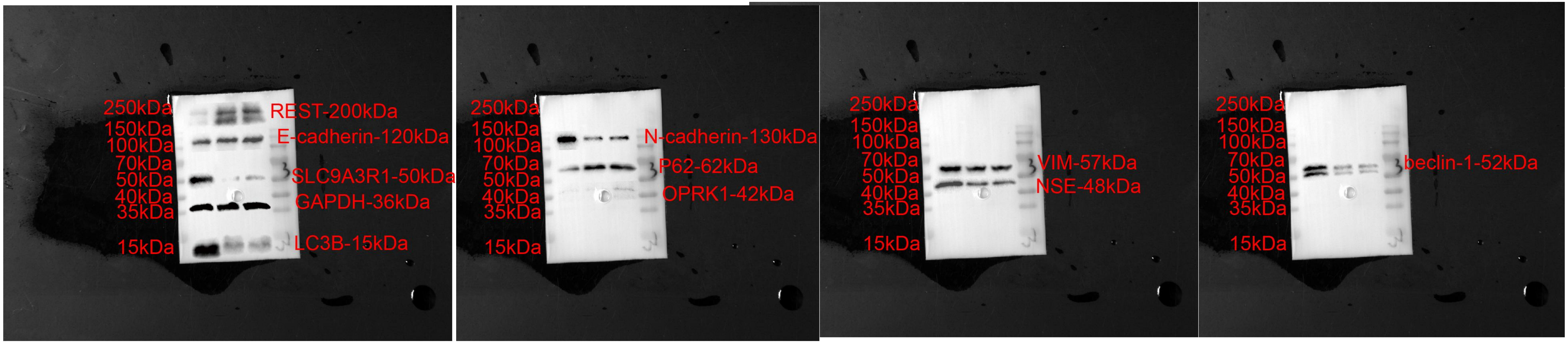

Figure S6O

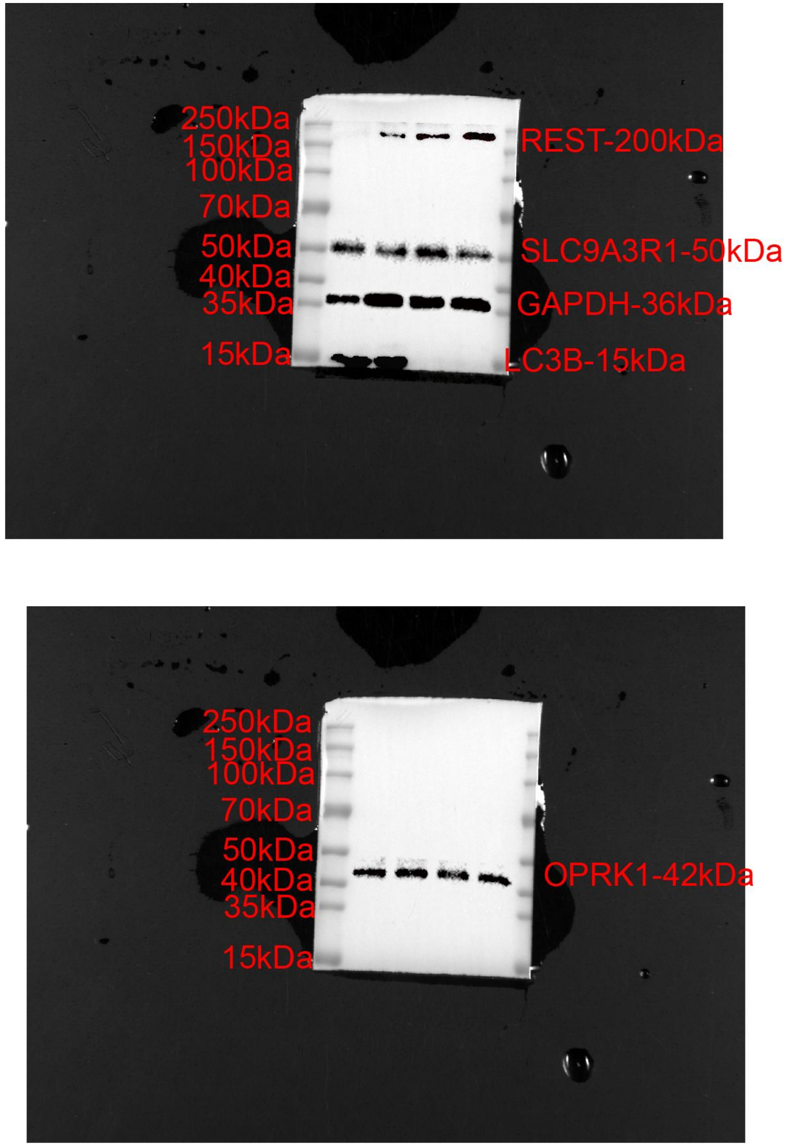

Figure S7C

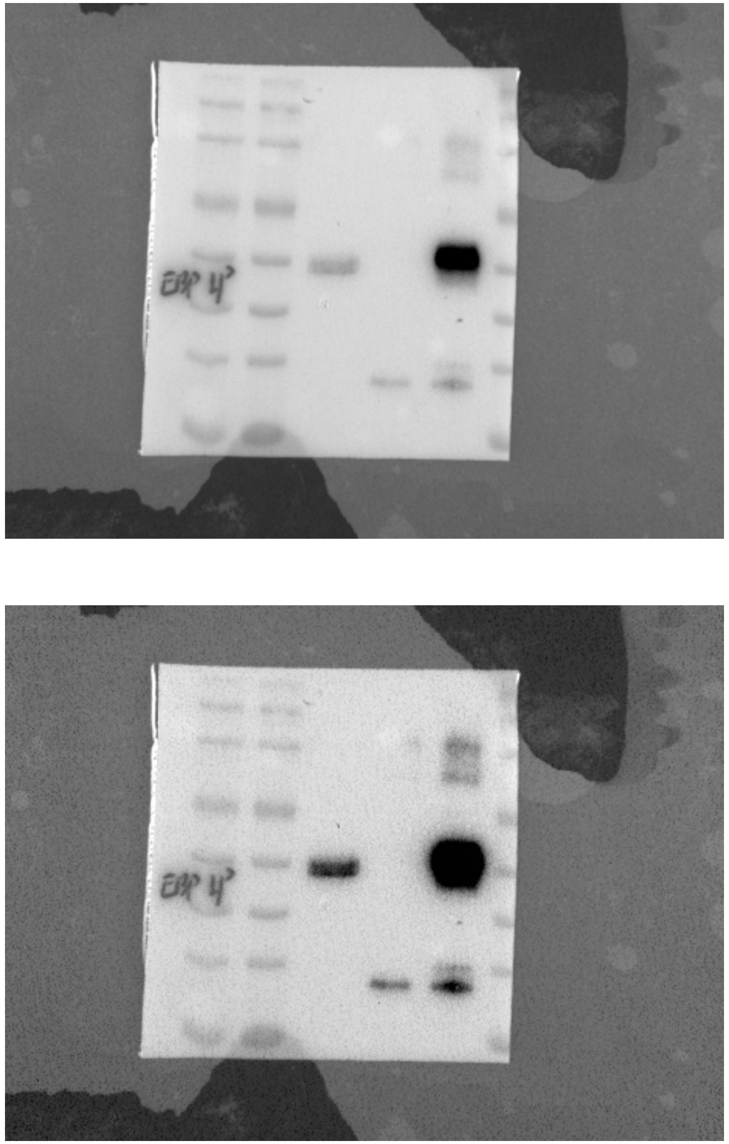

Figure S7D

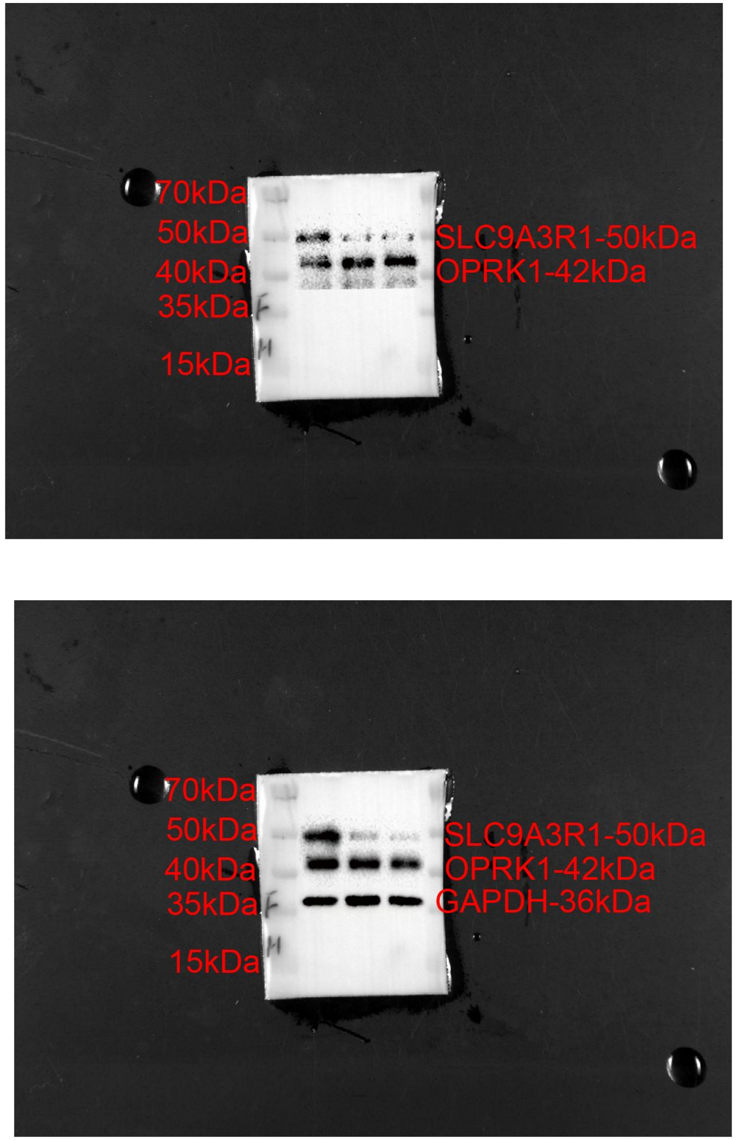

Figure S7E

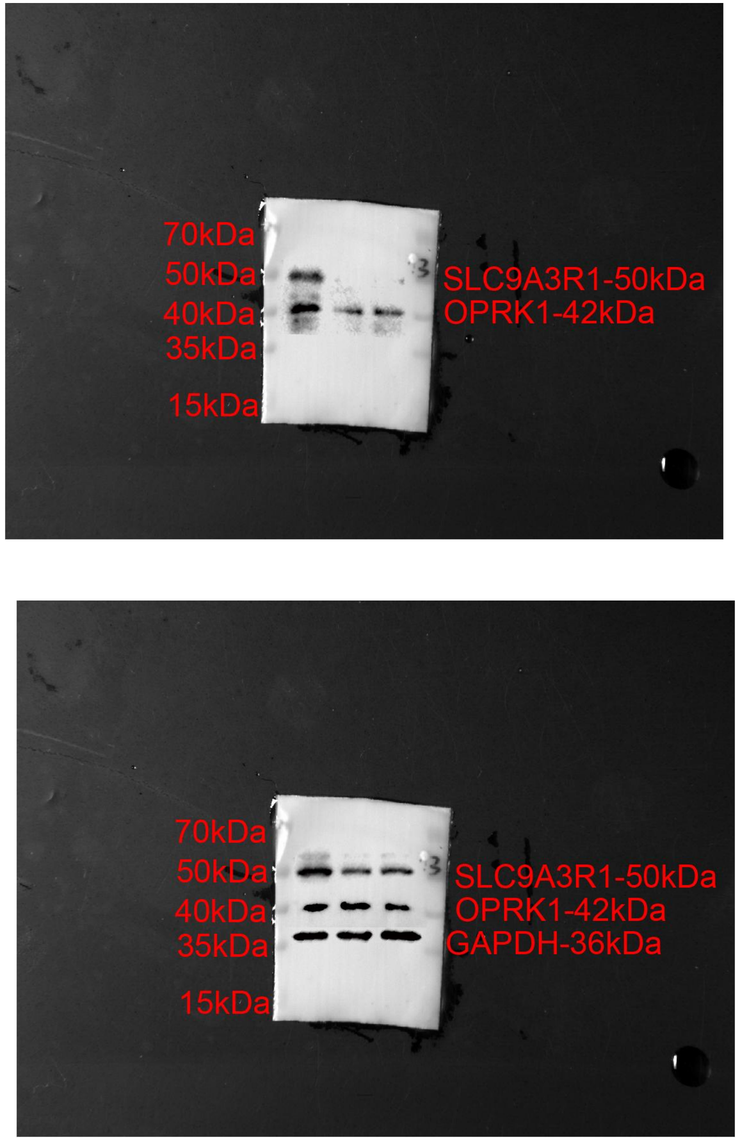

Figure S7F

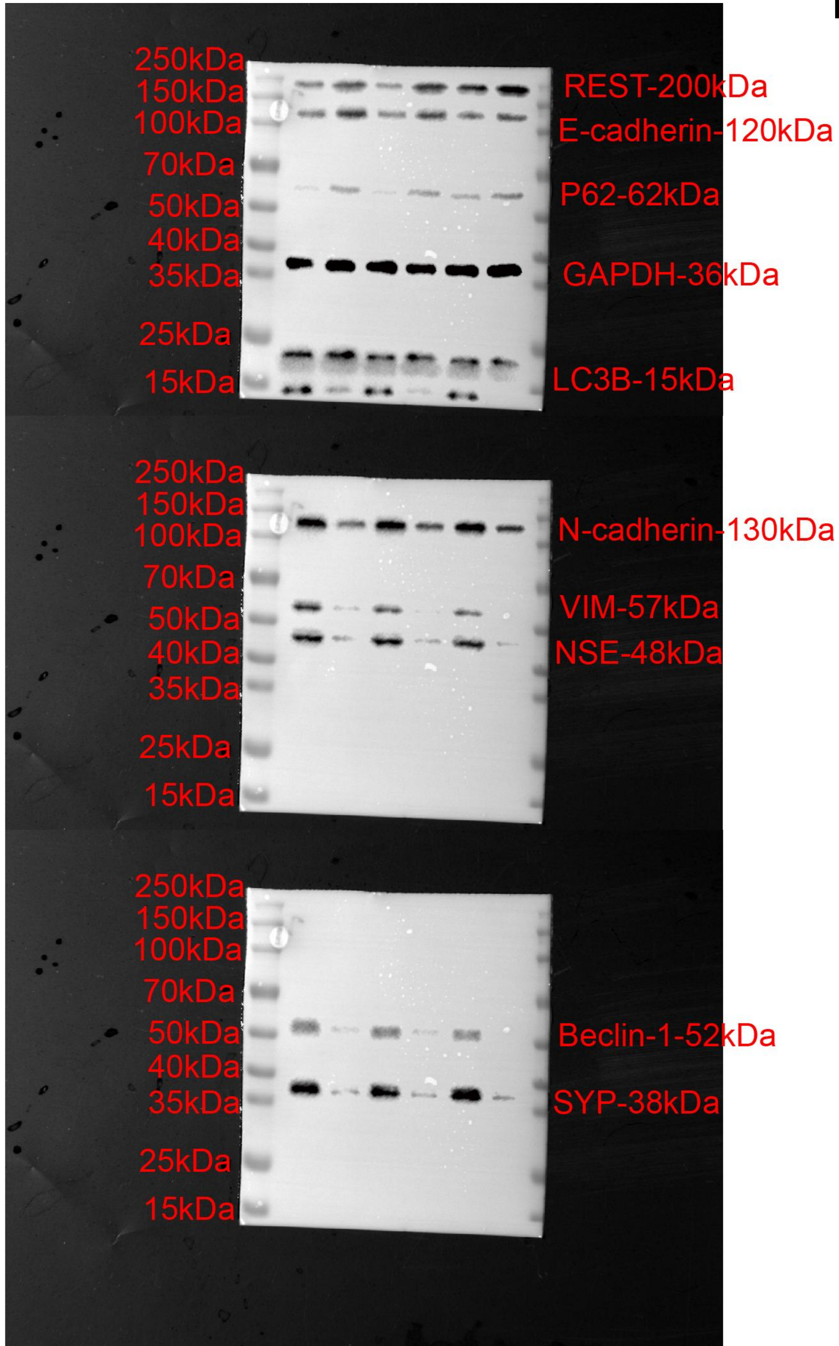

Figure S7H

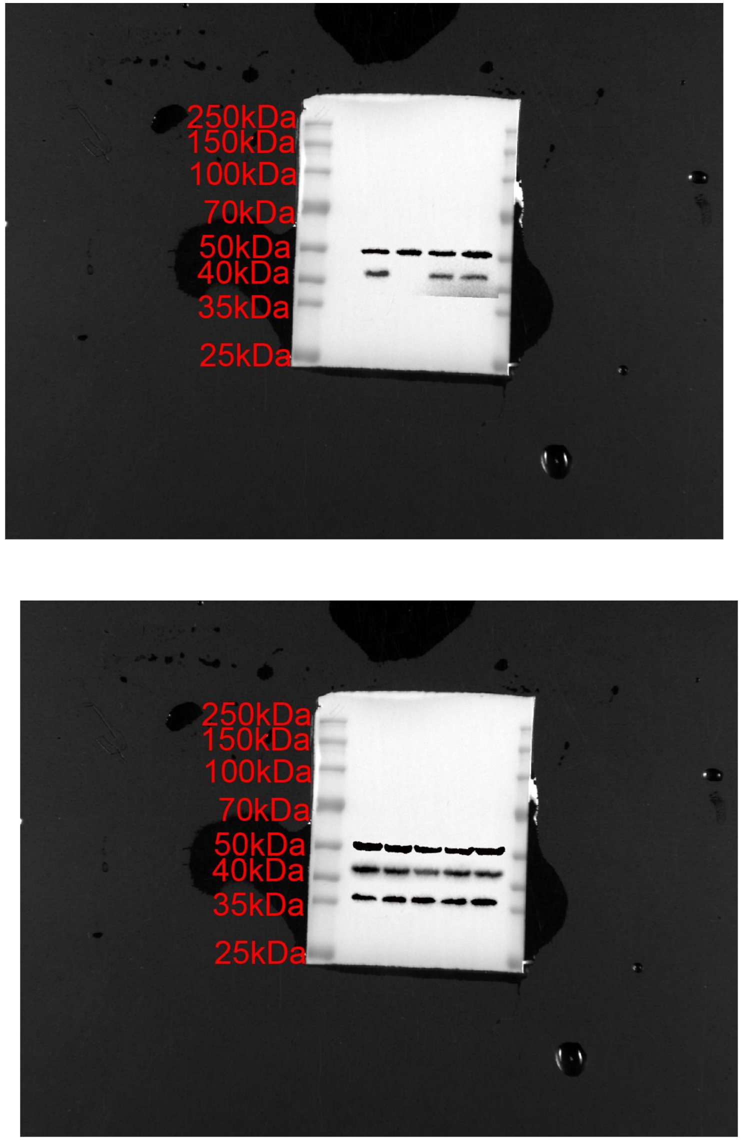

Figure S7I

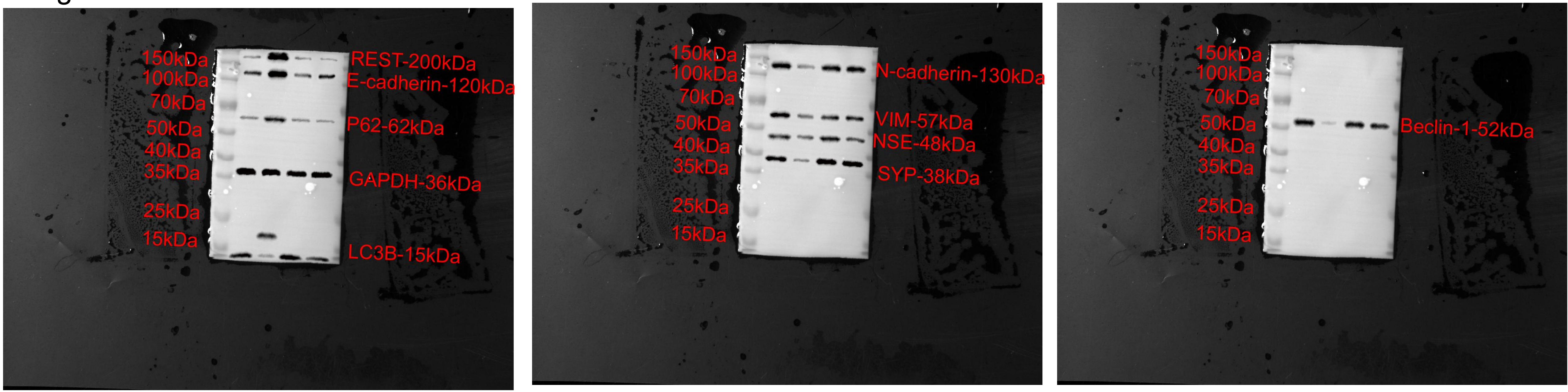

Figure S8A

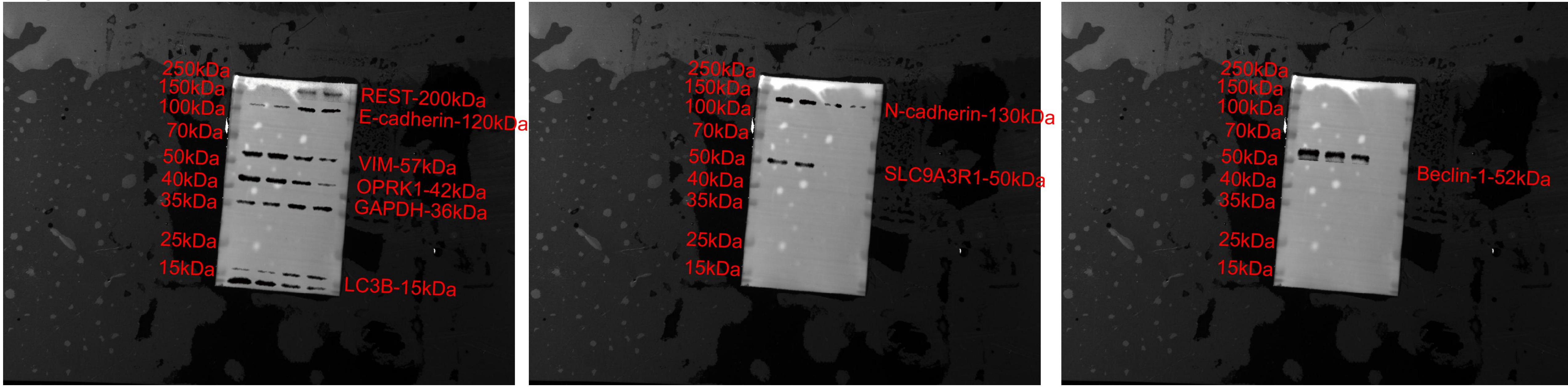

Figure S8D

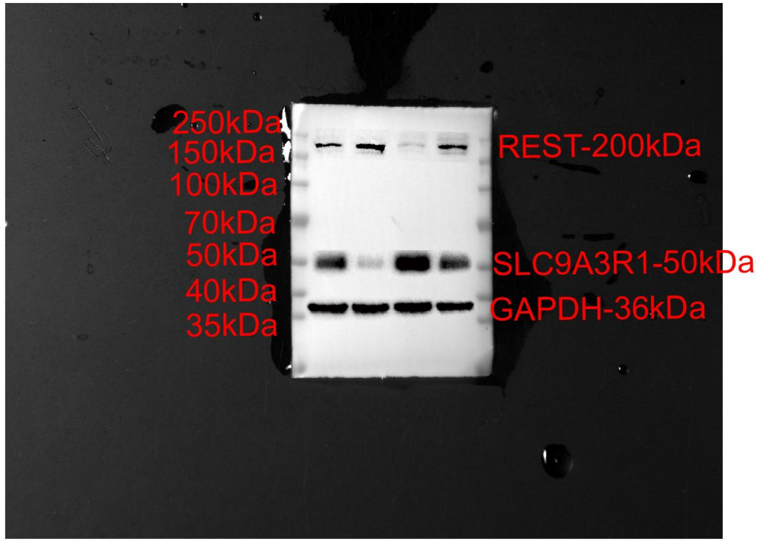
250kDa
150kDa
100kDa
70kDa
50kDa
40kDa
35kDa
REST-200kDa
SLC9A3R1-50kDa
GAPDH-36kDa

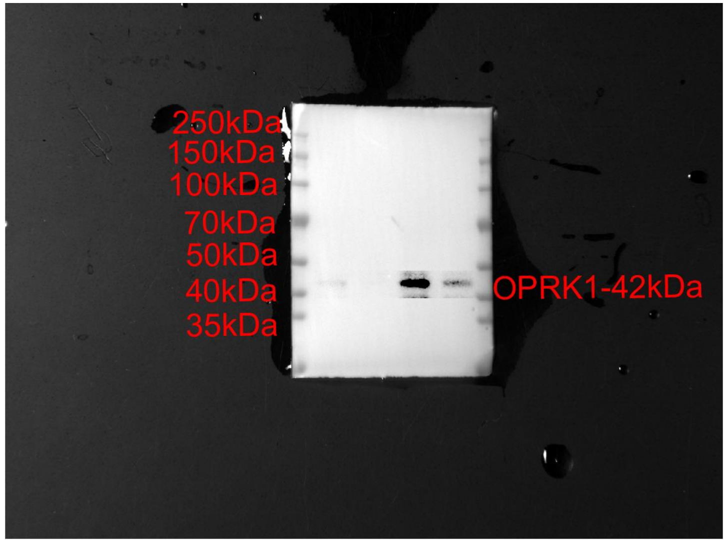
250kDa
150kDa
100kDa
70kDa
50kDa
40kDa
35kDa
OPRK1-42kDa
